# Supplementary figures and images for: Protocol for the PLAY Study: a randomised controlled trial of an intervention to improve infant development by encouraging maternal self-efficacy using behavioural feedback
Source: BMJ Open. 2023 Mar 7;13(3):e064976. doi: 10.1136/bmjopen-2022-064976 (PMC10008478; doi:10.1136/bmjopen-2022-064976)

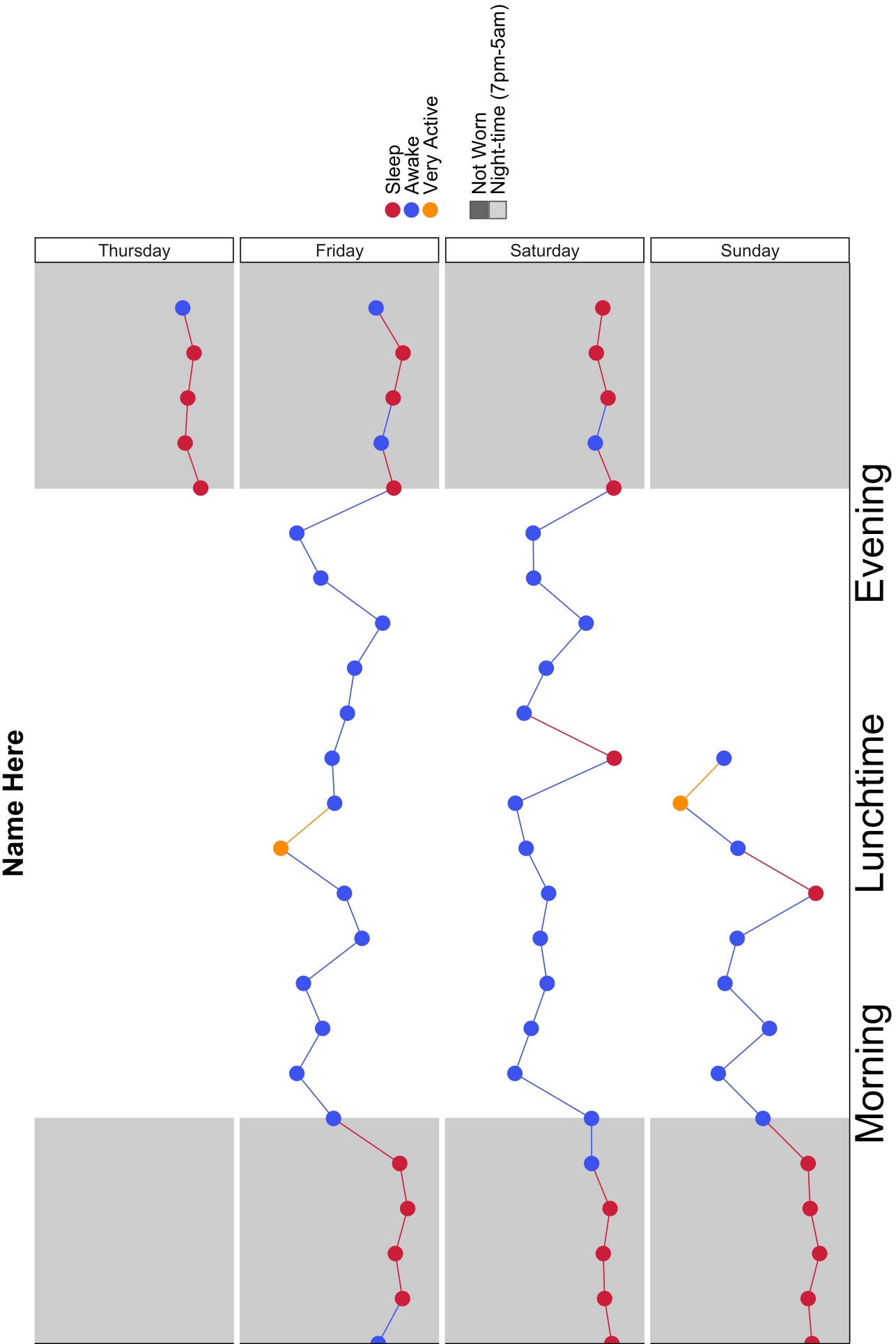

Supplement: Supplementary data [file bmjopen-2022-064976supp002.pdf]

**Supplementary Figure 2. Sleep, screen time, routines and daily activities**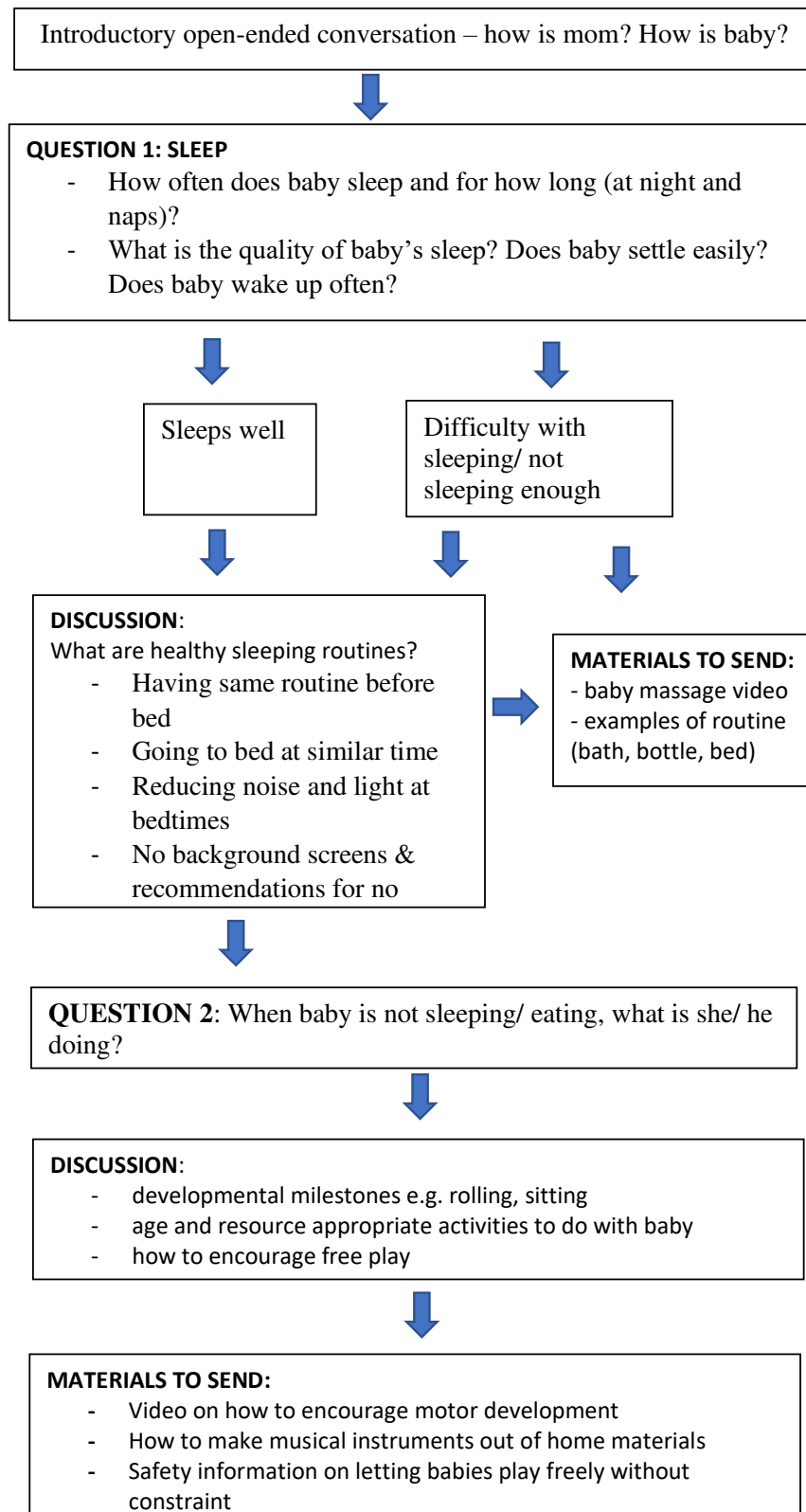

Supplement: Supplementary data [file bmjopen-2022-064976supp003.pdf]
