# Supplementary material for: Protocol for the PLAY Study: a randomised controlled trial of an intervention to improve infant development by encouraging maternal self-efficacy using behavioural feedback
Source: BMJ Open. 2023 Mar 7;13(3):e064976. doi: 10.1136/bmjopen-2022-064976 (PMC10008478; doi:10.1136/bmjopen-2022-064976)
Supplement: Supplementary data [file bmjopen-2022-064976supp001.pdf]

V2.1_20_10_2022

Play Love And You (PLAY) Study: Consent form

1. I have been given a Participant Information Sheet which explains the nature and processes involved in this study, which is attached hereto;
2. I was given time to read it, or had it read to me, in the language I best understand;
3. I was given time to ask any questions I wanted to and found any answers given to me to be reasonable and satisfactory;
4. I believe I fully understand why the study is being conducted and what the intended outcomes will be;
5. I understand that there will be no immediate benefit to me, should I agree to participate, nor will I receive any payment; conversely, participation will not cost me anything but my time;
6. I understand that, even if I initially consent to take part in the study, I may subsequently withdraw at any time and would not be required to give any reasons; if that happened, any data collected about me for the purposes of the study would immediately be destroyed, unless I give consent for it to be retained
7. I have been given a range of contact details, listed below. If I require further information or become concerned about any aspect of this study, I am free to speak to any of these contacts.

Contact details:

- Dr Alessandra Prioreschi, Principal Investigator, telephone no. 083 989 0070, or by e-mail at Alessandra.Prioreschi@wits.ac.za,
- Professor CB Penny, Chairperson of the Human Research Ethics Committee (Medical) at the University of Witwatersrand, on telephone no. 011 717 2301, or by e-mail at Clement.Penny@wits.ac.za.
- Ms. Z Ndlovu or Mr Rhulani Mkansi, Committee Secretariat, telephone nos.: 011 717 2700 or 1234, or by e-mail at: Zanele.Ndlovu@wits.ac.za or Rhulani.Mkansi@wits.ac.za
- Mrs Sarah Cantrell, Psychologist, Telephone no 0824649381 or by e-mail at sarahcantrellphd@gmail.com

Name of Participant: ____
Date: ____
Place: ____
Signature or mark ____

Witnessed by:

Name of Witness: ____
Signature: ____
Date: ____

V2.1_20_10_2022

CONSENT FORM FOR VISUAL RECORDING OF STUDY PARTICIPATION

I have read and understood the project information sheet, and I understand that it is up to me whether or not my videos are viewed by the research team. I understand that if I do not wish for my videos to be seen, it will not in any way affect how the interviewer treats me. I understand that my data will be shared with researchers in the United Kingdom. I understand that I can ask the research team to delete my videos, or any part of my videos at any time. I understand that the information that I give will be treated in the strictest of confidence, and that my name will not be used when the videos are analysed.

I hereby consent to visual recording of my interactions with my baby using the Headcam devices.

I understand that:

- The recording will be stored in a secure location (a locked cupboard or password protected computer) with restricted access by the researcher and the research supervisor.
- The recording will be transcribed and any information that could identify me will be removed from the transcription.
- My face and voice, and my baby's face and voice will be visible and audible in the recordings.
- The recordings will normally be erased within either (a) two (2) years of the publication of the research findings, or (b) six (6) years, if no publications arise from this research, or five years after the study.
- The film, with all identifying information directly linked to me removed, will be stored permanently and may be used for future research.
- Anyone wishing to access this information in the future will first have to obtain the approval of the Human Research Ethics Committee (Medical) of the University of the Witwatersrand, Johannesburg.

Name of Participant: ________________________________
Date: ________________________________
Place: ________________________________
Signature or mark ________________________________

Witnessed by:
Name of Witness: ________________________________
Signature: ________________________________
Date: ________________________________
