# Supplementary material for: Protocol for the PLAY Study: a randomised controlled trial of an intervention to improve infant development by encouraging maternal self-efficacy using behavioural feedback
Source: BMJ Open. 2023 Mar 7;13(3):e064976. doi: 10.1136/bmjopen-2022-064976 (PMC10008478; doi:10.1136/bmjopen-2022-064976)
Supplement: Author's manuscript [file bmjopen-2022-064976.draft_revisions.pdf]

BMJ Open is committed to open peer review. As part of this commitment we make the peer review history of every article we publish publicly available.

When an article is published we post the peer reviewers' comments and the authors' responses online. We also post the versions of the paper that were used during peer review. These are the versions that the peer review comments apply to.

The versions of the paper that follow are the versions that were submitted during the peer review process. They are not the versions of record or the final published versions. They should not be cited or distributed as the published version of this manuscript.

BMJ Open is an open access journal and the full, final, typeset and author-corrected version of record of the manuscript is available on our site with no access controls, subscription charges or pay-per-view fees (http://bmjopen.bmj.com).

If you have any questions on BMJ Open's open peer review process please email
info.bmjopen@bmj.com

BMJ Open

The PLAY study: a randomised controlled trail to improve infant neurodevelopment and growth by encouraging responsive caregiving through interactive play and biobehavioural feedback

Journal:	*BMJ Open*
Manuscript ID	bmjopen-2022-064976
Article Type:	Protocol
Date Submitted by the Author:	31-May-2022
Complete List of Authors:	Prioreschi, Alessandra; University of the Witwatersrand, SAMRC/Wits Developmental Pathways for Health Research Unit<br>Pearson, Rebecca; University of Bristol, Centre for Academic Mental Health, Addiction and Suicide Research, School of Social & Community Medicine; University of the Witwatersrand, SAMRC/Wits Developmental Pathways for Health Research Unit<br>Richter, Linda; University of the Witwatersrand, DSI-NRF Centre of Excellence in Child Development<br>Bennin, Fiona; University of the Witwatersrand, SAMRC/Wits Developmental Pathways for Health Research Unit<br>Theunissen, Helene; University of the Witwatersrand, SAMRC/Wits Developmental Pathways for Health Research Unit<br>Cantrell, Sarah J; University of the Witwatersrand, SAMRC/Wits Developmental Pathways for Health Research Unit<br>Maduna, Dumsile; University of the Witwatersrand, SAMRC/Wits Developmental Pathways for Health Research Unit<br>Lawlor, Deborah; Department of Social Medicine, University of Bristol, MRC Integrative Epidemiology Unit<br>Norris, Shane; University of the Witwatersrand Faculty of Health Sciences, SAMRC/Wits Developmental Pathways for Health Research Unit
Keywords:	Community child health < PAEDIATRICS, PUBLIC HEALTH, EPIDEMIOLOGY

SCHOLARONE™
Manuscripts

The PLAY study: a randomised controlled trail to improve infant neurodevelopment and growth by encouraging responsive caregiving through interactive play and biobehavioural feedback

Alessandra Prioreschi[1*], Rebecca Pearson[1,2], Linda Richter[3], Fiona Bennin[1], Helene Theunissen[1], Sarah J Cantrell[1], Dumsile Maduna[1], Deborah A Lawlor[4], Shane A Norris[1,5]

1. SAMRC/Wits Developmental Pathways for Health Research Unit, Department of Paediatrics and Child Health, Faculty of Health Sciences, University of the Witwatersrand, Johannesburg, South Africa
2. Centre for Academic Mental Health, School of Social and Community Medicine, University of Bristol, Bristol, UK.
3. DSI-NRF Centre of Excellence in Child Development, University of the Witwatersrand
4. MRC Integrative Epidemiology Unit at the University of Bristol, UK
5. School of Human Development and Health and NIHR Southampton Biomedical Research Centre, Global Health Research Institute, University of Southampton, Southampton, UK

* Corresponding Author:
Alessandra Prioreschi
Alessandra.prioreschi@wits.ac.za
SAMRC/Wits Developmental Pathways for Health Research Unit, Department of Paediatrics and Child Health, Faculty of Health Sciences, University of the Witwatersrand, Johannesburg, South Africa

For peer review only

For peer review only - http://bmjopen.bmj.com/site/about/guidelines.xhtml

Abstract

Introduction: The early infant caregiving environment is crucial in the formation of parent-child relationships, neurobehaviourial development, and thus child outcomes. This protocol describes the PLAY study, an intervention designed to promote infant neurodevelopment and growth through encouraging responsive parenting using interactive play, biobehavioral feedback, and supportive interventions.

Methods and analysis: 210 mother-infant pairs will be recruited at delivery from public health facilities in Soweto, South Africa, and individually randomised into two groups using computer generated block randomisation to ensure equal distribution between groups. The trial will consist of a standard of care arm and an intervention arm. The intervention will start at birth end at 24 months, and assessments will be made when the infants are 6-, 12- and 24-months of age. The intervention will be delivered by community health workers (CHWs) using an app, telephone calls, resource material and biobehavioural feedback with individualized support. Every 4-months mothers in the intervention group will receive rapid feedback via the app on their infant's movement behaviours, developmental milestones, and on their interaction styles with their infant. At recruitment, and again at 6 and 12 months, mothers will be screened for mental health and women who score in the high-risk category will receive an individual counselling session from a licensed psychologist, followed by referral and continued support as necessary. The primary outcome is infant neurodevelopment assessed using the Intergrowth-21st Neurodevelopment assessment package.

Ethics and dissemination: The PLAY study has ethical approval from the Human Research Ethics Committee of the University of the Witwatersrand (M220217)

Trial Registration: This trial was registered with the Pan African Clinical Trials Registry (https://pactr.samrc.ac.za) on 10/02/2022 (identifier: PACTR202202747620052)

Protocol version: 10/05/2022 (version #1.7)

Key words: Biobehavioural feedback, play, early child development, responsive caregiving

Strengths and limitations of the study:

- The PLAY study focuses specifically and intensively on key guidelines recommended by the WHO for promoting early child development: responsive caregiving, promotion of early learning, integration of nutrition and caregiving interventions, and supporting maternal mental health.

For peer review only - http://bmjopen.bmj.com/site/about/guidelines.xhtml

- The PLAY study uses biobehavioural feedback linked with personalised guidance to improve and individualise intervention content and implementation
- The PLAY study will be guided by an advisory group of community members (CAG) consisting of women with infants and their mothers/influential family matriarchs, and consultation with this advisory group will happen before and during the intervention to ensure we remain contextually aware in creating and delivering our study content
- The PLAY study relies to some extent on the availability of cell phone or computer access for the delivery of intervention content, which may be limiting in the Soweto setting. However, we will attempt to mitigate this by making the app data free, by making the app available on multiple platforms (web, mobile, android, Apple etc), and by continuously consulting with the CAG to ensure we come up with appropriate solutions if we encounter lack of access
- The COVID pandemic may interfere with our recruitment and intervention strategies, and we have made the study less reliant on face-to-face interactions to accommodate any further lockdown restrictions in South Africa. We will continue to consult with the CAG if COVID restrictions interfere with intervention delivery
- The PLAY study uses advanced and robust measurement tools, which in some cases are novel in South Africa and/or globally, thus providing reliable evidence which can inform future research.

For peer review only

Background

Over the last decade, emphasis globally is being placed on improving early childhood development (ECD) [1]. Estimates are that 250 million children under the age of five living in low-and-middle-income countries (LMICs) (43%) are at risk of not meeting their developmental potential [2]. Furthermore, a double burden of malnutrition persists in LMICs, with wasting present in 6.4% and overweight present in 6% of children under age 5 [3]. While optimising growth and development requires a life course approach, the first two years are key in optimising developmental potential [1 4]. This highly sensitive period (the first 1000 days of life) is an experience-dependent and –expectant process which is plastic and malleable [5]. The World Health Organisation (WHO) has recently published four key guidelines for improving ECD [6]: responsive caregiving, promotion of early learning, integration of nutrition and caregiving interventions, and supporting maternal mental health.

The importance of movement during infancy

Infant movement behaviours have been identified as important for early childhood growth and development [7]. Three categories of movement behaviours are of key importance: physical activity, sleep, and sedentary behaviours. Guidelines for these behaviours in the first few years of life encourage tummy time and active play, sufficient and regular sleep, and minimisation of activities that require infants to be restrained (eg: car seats, strollers, walkers) [7-9]. Play is defined as intrinsically motivated activities done for recreational purposes and enjoyment. Play is how children learn, explore, engage in physical activity and motor development, develop socially, emotionally and cognitively, and bond with caregivers [10 11]. Outdoor play is particularly encouraged due to its beneficial effects on both physical activity and development [12]. Additionally, interactive and stimulating play (such as reading, singing, and playing with other adults or children) is encouraged to promote development [13], and also supports early learning [6].

Formative research in South Africa has determined that infant boys spent more time doing higher intensity movement than girls from as early as 3-months [14]. Furthermore, higher intensity movement was associated with earlier attainment of developmental milestones [14], and with taller stature and lower abdominal adiposity [15]. However, the majority of infants in South Africa were not meeting recommended 24-hour movement guidelines [16]. When using synchronous 24-hour accelerometery worn by mothers and infants, we showed that increased physical activity among mothers while caring for their infants was associated with increased infant movement during the same period of time [17]. This highlights the important role of the mother in infant movement behaviours, and thus growth and development. Interactive play is particularly important for promotion of parent-child bonding, offering an ideal opportunity for parents to engage with their children through stimulating activities which encourage movement, and in turn promote cognitive, emotional and social development. Stimulation interventions in LMICs have been shown to improve home caregiving environments, mother-child interactions, and maternal health literacy around ECD [18].

Qualitative research examining maternal perceptions of the importance of play in infancy reported that mothers in South

For peer review only - http://bmjopen.bmj.com/site/about/guidelines.xhtml

Africa perceive developmental attainment as a key child outcome, indicating that interventions in this context should focus on promoting child development [19]. Mothers also reported significant barriers to encouraging play; including financial constraints, safety concerns and lack of certainty about how to promote play. Importantly, they did not perceive infant movement behaviours as behaviours that could be, or needed to be changed [19]. Additionally and similar to findings from national reports, mothers reported not reading to their children, and most children in this context did not attend creche, indicating the importance of intervening in the home environment to improve opportunities for early learning [19 20].

Supporting parenting behaviours for early childhood development

The early infant caregiving environment is crucial in the formation of parent-child relationships, mother-infant synchronicity and contingency, behaviour development, and thus child outcomes [13 21 22]. Mothers who are supported in their transition to parenthood have higher self-efficacy, and are less likely to develop past-partum depression [23 24]. Infants who have confident and healthy mothers are more likely to develop resilience and emotional and cognitive competencies, to have healthy growth trajectories, and to develop healthy behavioural patterns [25]. A recent systematic review and meta-analysis of parenting interventions to improve ECD in children under the age of three has shown that interventions with responsive caregiving components had greater effects on child cognitive development, as well as on parenting practices and parent-child interactions [23]. The authors recommended that future interventions should aim to directly support parent responsiveness and sensitivity.

Maternal self-efficacy is a likely contributor to responsiveness and sensitivity, and is determined by a mother's belief in her ability to care for her child, her emotional and psychological health, her child's temperament and health, and various environmental factors. Strategies to improve self-efficacy in parenthood include improving health literacy, reminding mothers of their natural ability to care for their child, social support, and promotion of mental health. Interventions that are targeted and focus specifically on changing maternal behaviour are most effective [26]. Maternal mental health should be supported through integration into ECD interventions and programmes, yet a lack of studies on maternal mental health that report on child health and development outcomes, in addition to mental health outcomes, has been identified as a critical gap [6]. Other gaps include identifying the most effective responsive caregiving interventions in LMICs, combining nutrition and caregiving strategies [6], and identifying ways to improve parenting behaviours without undermining self-efficacy. Qualitative findings from urban South Africa reported that infant nutrition interventions in this context should focus on: fostering maternal self-efficacy around exclusive breast-feeding; challenging mixed feeding practices and encouraging more responsive feeding approaches; and engaging family members to promote supportive household and community structures around infant feeding [27].

In the first 3-months postpartum, tools to sensitise mothers to infant's competencies and to promote positive and nurturing interactions between caregivers and infants are successful in reducing postpartum depression, and increasing self-efficacy

For peer review only - http://bmjopen.bmj.com/site/about/guidelines.xhtml

in both parents and health care providers [16 28]. Following early supportive intervention and sensitisation to infant behavioural cues, mothers benefit from continued support around breastfeeding, and promotion of healthy growth and development [16]. This can include provision of content to improve health literacy, practical tools and activities that promote infant development, continued sensitisation to infant behaviours and mother-infant interaction styles, and mental health promotion. Feedback is an important and effective component of parenting interventions, which can help to enhance parenting skills and thus child outcomes [28 29]. Feedback on parenting behaviours can be directive and/or responsive to behaviours in real-time, or following review of video recorded interactions [29 30]; and encouraging parents to practice skills following feedback is one of the strongest and most consistent predictors of improved parenting outcomes in meta-analyses [26 28]. Additionally, tailoring biobehavioural feedback is particularly effective for behaviour change in general [31].

Mother-infant interactions have primarily been measured using questionnaires or third party observation, yet these measures are limited by their subjectivity and intrusiveness. First-person observation using *headcams* - small cameras which are attached to headbands on both infants and mothers for a period of time while interacting at home - provide a first person view of the interaction from both infant and mother perspectives with more naturalistic and detailed measures. These *headcams* have been piloted in South Africa, and were found to be largely acceptable and feasible in this context [32].

Hypothesis

Using interactive play and biobehavioural feedback to support mothers in their ability to be responsive, promote early learning in the home, understand and engage with their infants' health behaviours (specifically movement behaviours and feeding), in conjunction with supporting mothers' mental health, will result in better infant growth and neurodevelopment in the first two years of life.

Aim

To test the efficacy of an intervention designed to promote neurodevelopment and growth through encouraging responsive parenting using interactive play and biobehavioral feedback.

Methods

The Play Love And You (PLAY) study was developed with the overriding theme that all an infant really needs are opportunities for movement and development (play), responsive and interactive caregiving (love), and the mother herself (you).

Study design

The PLAY study was designed following the Standard Protocol Items (Recommendations for Interventional Trials (SPIRIT) 2013 statement) [33]. Mother-infant pairs will be recruited in the third quarter of 2022, and individually randomised into two

For peer review only - http://bmjopen.bmj.com/site/about/guidelines.xhtml

groups (n=75 each) using computer generated block randomisation to ensure equal distribution between groups. The intervention will last from birth to 24 months, and assessments will be made when the infants are 6, 12- and 24-months of age. The trial will consist of a standard of care arm and an intervention arm – see Figure 1, and Supplementary Table 1. Data collection teams will be blinded to randomisation. Intervention delivery teams will work separately and independently of each other. Blinding will be maintained by the data manager and broken only for *a priori* defined analyses (Figure 2). Randomisation will be generated by the data manager at recruitment and intervention delivery teams will be assigned accordingly.

Participants

We will recruit 210 mother-infant pairs within 3 days following delivery at a community clinic in Soweto. Soweto is an urban-poor area in the City of Johannesburg covering 200 km^2 with over 1.3 million people (6400/km^2). Mother-infant pairs will be eligible to participate if:

- Mothers agree to participate for the duration of the study
- They reside in Soweto and plan to remain in Soweto for the study duration
- The mother is aged >=18 years
- The mother is the primary caregiver

Trial Intervention

Standard of care arm

Mothers assigned to the standard of care arm will receive the Road to Health booklet as well as the Side-by-Side services provided by the National Department of Health as routine postpartum care. This content is divided into 5 knowledge pillars (nutrition, love, protection, healthcare, and extra care), and functions to record child's growth and health interventions, provide information for caregivers, and encourage collaboration between healthcare workers and caregivers (https://sidebyside.co.za).

Additionally, they will be contacted telephonically every 2 months to check in, and to provide referrals to health care facilities if necessary

The standard of care arm will therefore include:

- Road to Health booklet as well as the Side-by-Side services provided as routine care postpartum at delivery
- Telephonic check in every 2 months, and referrals to health care facilities if necessary.

Intervention arm

For peer review only - http://bmjopen.bmj.com/site/about/guidelines.xhtml

Mothers assigned to the intervention arm will receive everything as per the standard of care arm. Additionally, they will receive interventions according to the following key intervention themes: 1) exclusive breastfeeding for 6-months, 2) interactive play and promotion of early learning, 3) responsive caregiving, and 4) maternal mental health. Each intervention theme will be prioritized during different phases of the trial and supported by components from the other themes. Figure 2 details when each theme will be prioritized and which supporting interventions will be included. Interventions will be delivered telephonically, and via a mobile App created for the purposes of this trial. Interventions are detailed below:

Biobehavioral feedback

Every 4-months mothers will receive rapid feedback via the app on their infant's movement behaviours, developmental milestones, and on their interaction styles with their infant. Feedback will be based on maternal reported milestone attainment in conjunction with objective measurement of infant movement behaviours and *headcam* video footage of interactive play in the home environment conducted every 3 months. This feedback will be provided along with personalised guidance related to play and interaction activities at home; and the app will allow mothers to observe changes in their infant's behaviour and development over the trial period.

Mental health screening and support

At recruitment, 6 months and 12 months, mothers will complete the Edinburgh Postnatal Depression Screening questionnaire (EPDS), the Post-traumatic stress syndrome Checklist 5 (PCL-5), the Adverse Childhood Experiences Scale (ACES), and the Antenatal Stress questionnaire (ASQ). Women who score in the high-risk category on one or more of these questionnaires will receive an individual counselling session from a licensed psychologist, followed by referral and continued support as necessary. Women who score at medium risk on one or more of these questionnaires will receive referrals from the licensed psychologist and continued support as necessary, and uptake of this support will be monitored.

Micro interventions

At recruitment, mothers will have the Newborn Behavioural Assessment (NBO)[34] administered by trained research staff. The NBO consists of 18 neurobehavioral observations along four dimensions — autonomic, motor, state organization and attentional-interactional - ultimately yielding a profile of the infant's behavioural repertoire. Importantly, the NBO allows for provision of infant specific anticipatory guidance, thus engaging parents in discussions around nurturing care, mother-infant interaction, play and development, and breastfeeding.

Thereafter, micro interventions will be delivered telephonically, and via the App. These micro interventions will consist of health literacy content in the format of quick and easy tips, short videos, infographics, or ideas for home-based activities. Telephonic conversations will comprise a scheduled series of topics as per the key intervention themes; but will allow mothers to guide the conversation by using open questions and providing anticipatory guidance. Content will be guided by a broad

For peer review only - http://bmjopen.bmj.com/site/about/guidelines.xhtml

range of pre-existing content such as UNICEF resources (https://www.unicef.org), and WHO recommendations [6], and other evidence based online resources. Contextually relevant material will also be co-developed with a community advisory group (CAG) for encouraging interactive play in the Soweto setting, and for encouraging support for breastfeeding from influential family matriarchs. Mothers will have continuous access to the App with all relevant resources, activities, and tools to support their interactions with their infant.

The intervention arm will thus include the standard of care arm and:

- Rapid biobehavioral feedback with individualized guidance on infant's development and movement behaviours, and mother-infant interaction styles
- Mental health screening with referrals and individual support as needed for high-risk mothers
- Micro interventions
    - NBO administered at recruitment
    - Telephonic conversations with discussions around key themes
    - Mobile App with health literacy resources and activities around key themes

Intervention Delivery

The intervention has been informed by the UK MRC Guidelines for Complex Interventions, and we used the TIDieR (Template for Intervention Description and Replication) Checklist, and is grounded in behaviour change techniques developed by Abraham and Michie [35]. Figure 3 details the logic model to illustrate the pathways to impact that guided the intervention approach. The agents of change are community health workers (CHWs), who do not need to have any specific qualification or expertise in health promotion, but will be trained in community health promotion, as well as relevant intervention methodology. The CHWs are supported by a supervisor with access to nurses and a referral network.

The intervention will be delivered by a combination of phone calls, app-based personalised biobehavioural feedback and guidance; as well as individual counselling sessions for high-risk mothers. The content delivery will be guided by a schedule following key themes according to infant age and developmental stage. Phone calls will be guided by a conversation flow chart, which will start with CHWs asking mothers how they are doing and gently introducing the theme for discussion using open-ended questions. The flow chart will be designed to allow CHWs to either ask follow up questions, or to provide anticipatory guidance based on mothers' individual responses (example provided in Supplementary Figure 1). This will allow mothers to guide the conversation, while still following key themes for discussion. CHWs will refer mothers as necessary if any concerns are raised during these conversations.

Outcome measures

For peer review only - http://bmjopen.bmj.com/site/about/guidelines.xhtml

Data collection will be conducted by independent, trained research assistants. The primary outcome measure is infant neurodevelopment assessed at 24-months using the Intergrowth-21st Neurodevelopment assessment package compared between control and intervention participants. Additionally, interim assessments at 6 months and 12 months will measure secondary outcomes of interest according to Figure 2.

Primary outcome:

At the final visit, infant development will be assessed using the Intergrowth-21st Neurodevelopment assessment package[7]. This package assesses toddler neurodevelopment objectively in various domains including cognitive development, executive function, attention, fine motor skills, gross motor skills, vision, language, behaviour, emotional reactivity, and positive affect. This tool has been successfully utilised in various LMICs, including Soweto, South Africa, and was designed to be used across a variety of settings by non-specialist assessors while not being affected by cultural influences[8].

Secondary outcomes at 24 months:

- Individual domains of the Intergrowth-21st Neurodevelopment assessment package compared between intervention and control groups
- Change in infant weight to length ratio in the first 24 months of life compared between intervention and control groups
- Infant fat mass index at 24-months (assessed using DXA fat mass divided by length) compared between intervention and control groups

Secondary outcomes at interim assessments:

- Breastfeeding self-efficacy from 0-6 months compared between intervention and control groups
- Health literacy from 0-12 months compared between intervention and control groups

Patient and Public Involvement

Prior to trial implementation, a community advisory group (CAG) (n=16) of mothers from Soweto with infants aged 0-3 months will be established to serve as experts on the lived experience of being a mother to an infant in Soweto. For about half of these women, family matriarchs will be recruited to form a separate CAG (n=8). Research from this context has indicated that family matriarchs play an important role in the choices that mothers make around infant feeding and nutrition, and as such it is pertinent they be included in interventions targeting breastfeeding and other child care practices.

Intervention content and activities will be tested and/or co-developed with the CAG, using focus group discussions and piloting of intervention content. Thus, acceptability and feasibility of the intervention content will be tested by the CAG in order to understand any changes required and thus refine the intervention accordingly.

For peer review only - http://bmjopen.bmj.com/site/about/guidelines.xhtml

The CAG will continue to advise throughout the duration of the intervention, via regular (every 4 months) focus group discussions (FGDs) where the research team will raise queries about intervention content, delivery and uptake, retention strategies, and feedback mechanisms. Additionally, the CAG will provide important insight into their lived experience of being a mother in Soweto over time and as their infant grows. This will provide useful insight to guide intervention conversations, and to better understand the context within which the intervention is being delivered.

Process evaluation

We will follow the MRC guidance on process evaluation of complex interventions (https://www.mrc.ac.uk/documents/pdf/mrc-phsrn-process-evaluation-guidance-final/). Evaluation will focus on implementation, mechanisms of impact, and context.

- Compliance will be assessed by counts (e.g.: login frequency on app, delivery of messages, and monitoring of phone calls using a log), and by discussions with the CHWs.
- Intervention dose will be monitored by recording the frequency and duration of contact between participants and CHWs using the logs.
- Infant adherence to movement guidelines will be assessed in the intervention group every 4 months using accelerometery. Seven-day, 24-hour wrist worn accelerometery data (Axivity-AX3 worn on both mother and infant) will be collected, downloaded (omgui, Open Movement, UK), auto-calibrated, and processed. Data is summarised to generate average acceleration (mg) per 5-second interval, as well as overall time (hourly and weekly) spent in varying intensities of activity, which will allow for determination of adherence to movement guidelines, and changes in activity levels over the intervention period.
- Mother-infant interactions will be measured in the intervention group during play using first person observation and behavioural analysis every 4 months. *Headcam* measures will be collected during at least five, 3-5 minute play interactions over the course of a week. *Headcam* observations will be coded using Noldus Observer XT software. An event-based coding framework will be used to categorise second-by-second key maternal and infant behaviours, i.e.: touch, play, vocalisations, and gaze. This allows for determination of the probability of certain behaviour preceding or following infant play. By combining measures of movement behaviours and mother-infant interactions; intensity and frequency of movement during interactive play, as well as interaction styles during play will be determined for feedback to mothers.
- To understand the mechanisms through which the intervention brings about change in specified outcomes, the acceptability of the intervention, and women's experiences of contact with CHWs will be assessed using interviews and focus groups at the close of the intervention.
- In considering the context, identification of factors that might act as barriers or facilitators to intervention implementation or effects will be assessed. This will include local and national policy, local service configuration and

For peer review only - http://bmjopen.bmj.com/site/about/guidelines.xhtml

provision, and socio-demographic and environmental factors of the participants. Our approach to assessing context will be through a combination of policy mapping, quantitative data collection, and longitudinal consultation with the CAG.

Monitoring

Monitoring of intervention activities will be done throughout the trial. Each contact will be recorded in order to be able to monitor intervention intensity and frequency. Engagement with the app and biobehavioural feedback content (number of page visits and duration of time spent watching videos) will be monitored via the app.

Adherence will be assessed every 4 months, and strategies to improve adherence will be discussed with the CAG as necessary. Additionally, the research team will meet with CHWs monthly to determine any concerns or issues with compliance or retention, and strategies will be discussed accordingly. Any adverse events will be recorded and reported as necessary according to standard operating procedures.

Sample size

Given expected attrition (30%), recruiting 210 mother-infant pairs should yield a sample size of 150 mother-infant pairs divided into two groups (n=75 each), which will provide 80% power to detect a developmentally important centile crossing difference (0.3SD) in the cognitive subscale of the INTER-NDA neurodevelopment assessment[36] between the intervention and standard care arms based on an expected mean (SD) of 36.8(6.5) at 24 months in the control arm, with a two-sided alpha of 0.05.

Attrition will be minimised by maintaining regular contact with both groups, by recruiting participants who plan on remaining in Soweto, and by using a flexible and versatile delivery approach including in-person group sessions, app based content, telephonic sessions, mobile messaging, and site visits.

Data analysis plan

The efficacy of the intervention will be assessed using intention to treat analysis. Primary and secondary outcome data will be compared between the intervention and standard of care arms using Student's unpaired t-test. Focus will be placed on the primary outcome due to limited power for secondary outcomes, which will therefore be treated with caution and presented using point estimates and precision (95% CI). Sensitivity analyses will include comparing baseline characteristics between intervention and standard of care arms, examining the effect of intervention dose on outcome data, and stratifying analyses by sex.

Data management

For peer review only - http://bmjopen.bmj.com/site/about/guidelines.xhtml

REDCap will be used for data management (https://www.project-redcap.org). Secure Sockets Layer (SSL) provides the needed security for the REDCap similar to what a VPN will provide. The Wits University server hosting REDCap is resilient and hardened against hacking and other forms of attacks. All data traffic is encrypted, and an installed network firewall provides secure network access. The REDCap system provides inherent application-level security including access controls and comprehensive audit trails; and all incoming requests get intentionally filtered, sanitized and escaped to prevent against attacks.

Data files and metadata will be uploaded and stored on REDCap by the project data manager, on a password protected Wits server for the duration of the project to enable team access. REDCap is used for data and metadata storage, as well as for data collection. Data will be inputted online with secure web authentication, data logging and SSL encryption. Accelerometery files and video files, as well as focus group discussion notes and transcripts will be stored on a secure and encrypted laptop, as well as on an external hard drive, and backed up as meta-data to REDCap. Regular back-up copies will be made by the project data manager on password protect file space. When the project is completed, the full database will be stored on both DPHRU and University servers. The items and metadata on the institutional repository are backed up on a daily basis and these backups are kept on tape. All data collection devices will be encrypted.

Data from this study will be stored for a minimum of 10 years from the completion of the study in order to allow for any verification of data. Long-term data storage will be in .csv files using a standard (simple) ASCII coding scheme in line with UK Data Service recommendations:
(https://www.ukdataservice.ac.uk/manage-data/format/recommended-formats).

Acknowledgements

The authors would like to thank the Wellcome Trust and the NIHR for their financial support which made this study possible.

Contributors

AP is the principle investigator for the PLAY study. AP, SAN, RP, LR and DAL conceptualised the study design. AP wrote the initial draft of the protocol. SAN, RP, LR, DAL, DM, FB, SJC, and HT read and contributed to the final version. All authors provided edits and critiqued the manuscript for intellectual content.

Funding

For peer review only - http://bmjopen.bmj.com/site/about/guidelines.xhtml

This work was supported by the National Institute for Health Research (NIHR) (using the UK's Official Development Assistance (ODA) Funding) and Wellcome [222007/Z/20/Z] under the NIHR-Wellcome Partnership for Global Health Research. The views expressed are those of the authors and not necessarily those of Wellcome, the NIHR or the Department of Health and Social Care.

Competing interests

The authors have no conflicts of interest to report

Patient and public involvement

Participants and the public are involved in the design, or conduct, or reporting, or dissemination plans of this research. Refer to the Methods section for further details.

Patient consent for publication

Not required.

Ethical approval

Ethical approval has been granted for the establishment of the community advisory group (M210846), and for the trial (M220217) from the Human Ethics Research Committee of the University of the Witwatersrand, Johannesburg, South Africa. Additional approval has been granted by the Research Committee of Johannesburg Health District (GP_202202_021).

Provenance and peer review

Not commissioned; peer-reviewed for ethical and funding approval prior to submission.

For peer review only

References

1. Black MM, Walker SP, Fernald LCH, et al. Early childhood development coming of age: science through the life course. *The Lancet* 2017;389(10064):77-90. doi: 10.1016/s0140-6736(16)31389-7
2. Lu C, Black MM, Richter LM. Risk of poor development in young children in low-income and middle-income countries: an estimation and analysis at the global, regional, and country level. *The Lancet Global Health* 2016;4(12):e916-e22. doi: 10.1016/s2214-109x(16)30266-2
3. Collaborators LBDDBoM. Mapping local patterns of childhood overweight and wasting in low- and middle-income countries between 2000 and 2017. *Nat Med* 2020;26(5):750-59. doi: 10.1038/s41591-020-0807-6 [published Online First: 2020/04/22]
4. Aboud FE, Yousafzai AK. Global health and development in early childhood. *Annu Rev Psychol* 2015;66:433-57. doi: 10.1146/annurev-psych-010814-015128 [published Online First: 2014/09/10]
5. Cantor P, Osher D, Berg J, et al. Malleability, plasticity, and individuality: How children learn and develop in context1. *Applied Developmental Science* 2018;23(4):307-37. doi: 10.1080/10888691.2017.1398649
6. World Health Organization. Improving early childhood development: WHO guideline, 2020.
7. World Health Organization. Guidelines on physical activity, sedentary behaviour and sleep for children under 5 years of age. *Geneva: Licence: CC BY-NC-SA 30 IGO* 2019
8. Draper CE, Tomaz SA, Biersteker L, et al. The South African 24-Hour Movement Guidelines for Birth to 5 Years: An Integration of Physical Activity, Sitting Behavior, Screen Time, and Sleep. *J Phys Act Health* 2020;17(1):109-19. doi: 10.1123/jpah.2019-0187 [published Online First: 2019/12/27]
9. Tremblay M, Chaput J, Adamo K, et al. Canadian 24-Hour Movement Guidelines for the Early Years (0–4 years): An Integration of Physical Activity, Sedentary Behaviour, and Sleep. *BMC Public Health* 2017;17 (Suppl 5):874. doi: 10.1186/s12889-017-4859-6
10. Ginsburg KR, American Academy of Pediatrics Committee on C, American Academy of Pediatrics Committee on Psychosocial Aspects of C, et al. The importance of play in promoting healthy child development and maintaining strong parent-child bonds. *Pediatrics* 2007;119(1):182-91. doi: 10.1542/peds.2006-2697 [published Online First: 2007/01/04]
11. Markova G. The Games Infants Play: Social Games During Early Mother-Infant Interactions and Their Relationship With Oxytocin. *Front Psychol* 2018;9:1041. doi: 10.3389/fpsyg.2018.01041 [published Online First: 2018/07/11]
12. Tremblay MS, Gray C, Babcock S, et al. Position Statement on Active Outdoor Play. *Int J Environ Res Public Health* 2015;12(6):6475-505. doi: 10.3390/ijerph120606475 [published Online First: 2015/06/11]
13. World Health Organization, United Nations Children's Fund, Group. WB. Nurturing care for early childhood development: a framework for helping children survive and thrive to transform health and human potential. Geneva: World Health Organization, 2018.
14. Prioreschi A, Brage S, Hesketh KD, et al. Describing objectively measured physical activity levels, patterns, and correlates in a cross sectional sample of infants and toddlers from

For peer review only

South Africa. *Int J Behav Nutr Phys Act* 2017;14(1):176. doi: 10.1186/s12966-017-0633-5 [published Online First: 2017/12/24]
15. Prioreschi A, KK O, E DLR, et al. Associations between abdominal adiposity, body size and objectively measured physical activity in infants from Soweto, South Africa. *Maternal and Child Health* 2022;In press doi: DOI: 10.1007/s10995-022-03406-5
16. Prioreschi A, Micklesfield LK. Compliance With Physical Activity and Sedentary Behavior Guidelines and Associations With Abdominal Adiposity in a Sample of Infants and Toddlers From Soweto, South Africa. *J Phys Act Health* 2019;16(12):872-79. doi: 10.1123/jpah.2018-0182 [published Online First: 2019/07/19]
17. Prioreschi A, Brage S, Westgate K, et al. Describing the diurnal relationships between objectively measured mother and infant physical activity. *Int J Behav Nutr Phys Act* 2018;15(1):59. doi: 10.1186/s12966-018-0692-2 [published Online First: 2018/06/27]
18. Jeong J, Pitchik HO, Yousafzai AK. Stimulation Interventions and Parenting in Low- and Middle-Income Countries: A Meta-analysis. *Pediatrics* 2018;141(4) doi: 10.1542/peds.2017-3510 [published Online First: 2018/03/04]
19. Prioreschi A, Wrottesley SV, Slemming W, et al. A qualitative study reporting maternal perceptions of the importance of play for healthy growth and development in the first two years of life. *BMC Pediatr* 2020;20(1):428. doi: 10.1186/s12887-020-02321-4 [published Online First: 2020/09/11]
20. Hall K, Richter L, Mokomane Z, et al. South African Child Gauge 2018. *Cape Town: Children's Institute, University of Cape Town ISBN: 978-0-620-81934-3* 2018
21. Richter L. The importance of caregiver-child interactions for the survival and healthy development of young children: a review. *World Health Organisation* 2004
22. Bernard K, Meade EB, Dozier M. Parental synchrony and nurturance as targets in an attachment based intervention: building upon Mary Ainsworth's insights about mother-infant interaction. *Attach Hum Dev* 2013;15(5-6):507-23. doi: 10.1080/14616734.2013.820920 [published Online First: 2013/12/05]
23. Jeong J, Franchett EE, Ramos de Oliveira CV, et al. Parenting interventions to promote early child development in the first three years of life: A global systematic review and meta-analysis. *PLoS Med* 2021;18(5):e1003602. doi: 10.1371/journal.pmed.1003602 [published Online First: 2021/05/11]
24. Brazeau N, Reisz S, Jacobvitz D, et al. Understanding the Connection between Attachment Trauma and Maternal Self-Efficacy in Depressed Mothers. *Infant Ment Health J* 2018;39(1):30-43. doi: 10.1002/imhj.21692 [published Online First: 2017/12/28]
25. Leerkes E, Crockenberg S. The Development of Maternal Self-Efficacy and Its Impact on Maternal Behavior. *Infancy* 2002;3(2):227-47.
26. Dozier M, Bernard K. Attachment and Biobehavioral Catch-up: Addressing the Needs of Infants and Toddlers Exposed to Inadequate or Problematic Caregiving. *Curr Opin Psychol* 2017;15:111-17. doi: 10.1016/j.copsyc.2017.03.003 [published Online First: 2017/06/27]
27. Wrottesley SV, Prioreschi A, Slemming W, et al. Maternal perspectives on infant feeding practices in Soweto, South Africa. *Public Health Nutr* 2020:1-13. doi: 10.1017/S1368980020002451 [published Online First: 2020/08/11]
28. Kaminski JW, Valle LA, Filene JH, et al. A meta-analytic review of components associated with parent training program effectiveness. *J Abnorm Child Psychol* 2008;36(4):567-89. doi: 10.1007/s10802-007-9201-9 [published Online First: 2008/01/22]
29. Caron EB, Bernard K, Dozier M. In Vivo Feedback Predicts Parent Behavior Change in the Attachment and Biobehavioral Catch-up Intervention. *J Clin Child Adolesc Psychol*

For peer review only - http://bmjopen.bmj.com/site/about/guidelines.xhtml

2018;47(sup1):S35-S46. doi: 10.1080/15374416.2016.1141359 [published Online First: 2016/04/05]
30. Van Zeijl J, Mesman J, Van IMH, et al. Attachment-based intervention for enhancing sensitive discipline in mothers of 1- to 3-year-old children at risk for externalizing behavior problems: a randomized controlled trial. *J Consult Clin Psychol* 2006;74(6):994-1005. doi: 10.1037/0022-006X.74.6.994 [published Online First: 2006/12/13]
31. Richards K, Enderlin C, Beck C, et al. Tailored Biobehavioral Interventions: A Literature Review and Synthesis. *Research and Theory for Nursing Practice* 2007;21(4):271-85.
32. Wrottesley S, Pearson RM, Prioreschi A. The headcam caregiver-infant interaction assessment tool: testing the feasibility and acceptability in Soweto, South Africa using participatory engagement. *In review* 2020
33. Chan AW, Tetzlaff JM, Gotzsche PC, et al. SPIRIT 2013 explanation and elaboration: guidance for protocols of clinical trials. *BMJ* 2013;346:e7586. doi: 10.1136/bmj.e7586 [published Online First: 2013/01/11]
34. Nugent JK, Bartlett JD, Von Ende A, et al. The Effects of the Newborn Behavioral Observations (NBO) System on Sensitivity in Mother–Infant Interactions. *Infants & Young Children* 2017;30(4):257-68. doi: 10.1097/iyc.0000000000000103
35. Michie S, van Stralen MM, West R. The behaviour change wheel: a new method for characterising and designing behaviour change interventions. *Implement Sci* 2011;6:42. doi: 10.1186/1748-5908-6-42 [published Online First: 2011/04/26]
36. Fernandes M, Villar J, Stein A, et al. INTERGROWTH-21st Project international INTER-NDA standards for child development at 2 years of age: an international prospective population-based study. *BMJ Open* 2020;10(6):e035258. doi: 10.1136/bmjopen-2019-035258 [published Online First: 2020/06/10]

For peer review only

Figures

Figure 1. Participant flow chart

Figure 2. Intervention themes and assessments

Figure 3. PLAY Study logic model

For peer review only

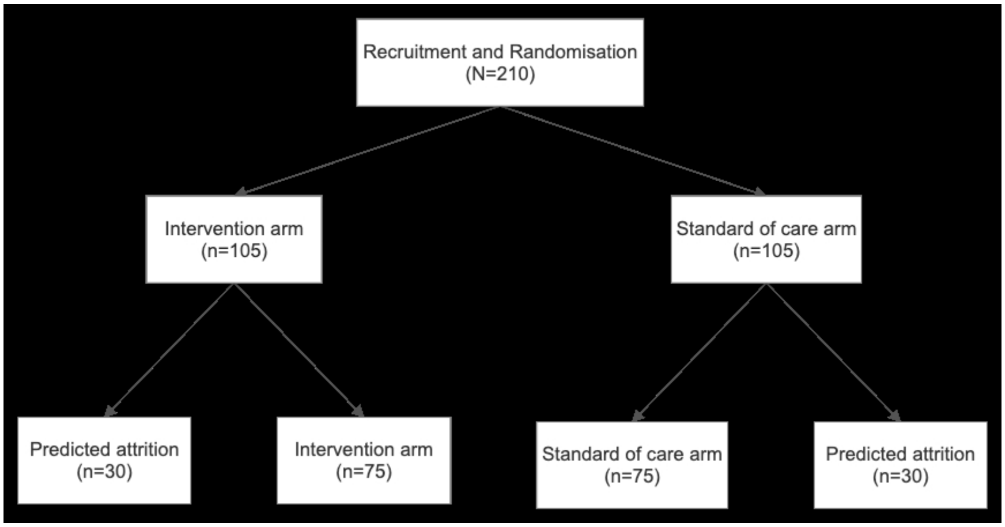

Figure 1. Participant flow chart

305x158mm (72 x 72 DPI)

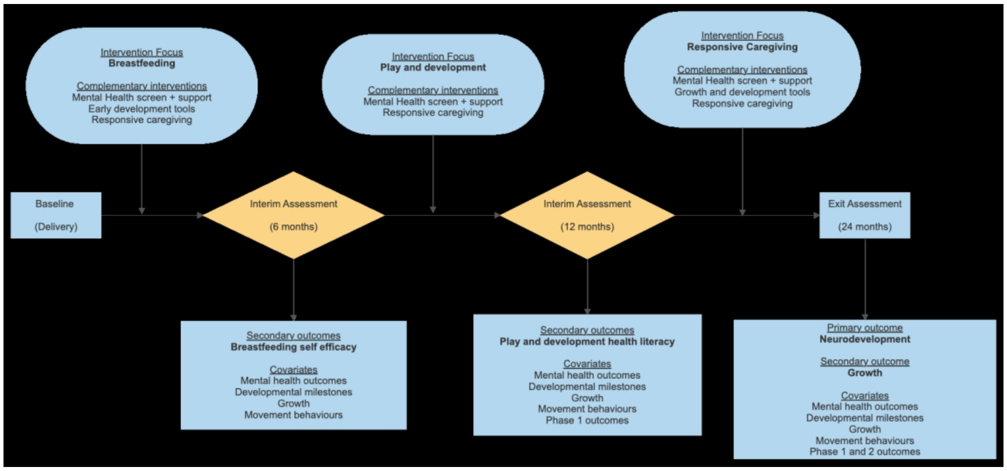

Figure 2. Intervention themes and assessments

587x271mm (72 x 72 DPI)

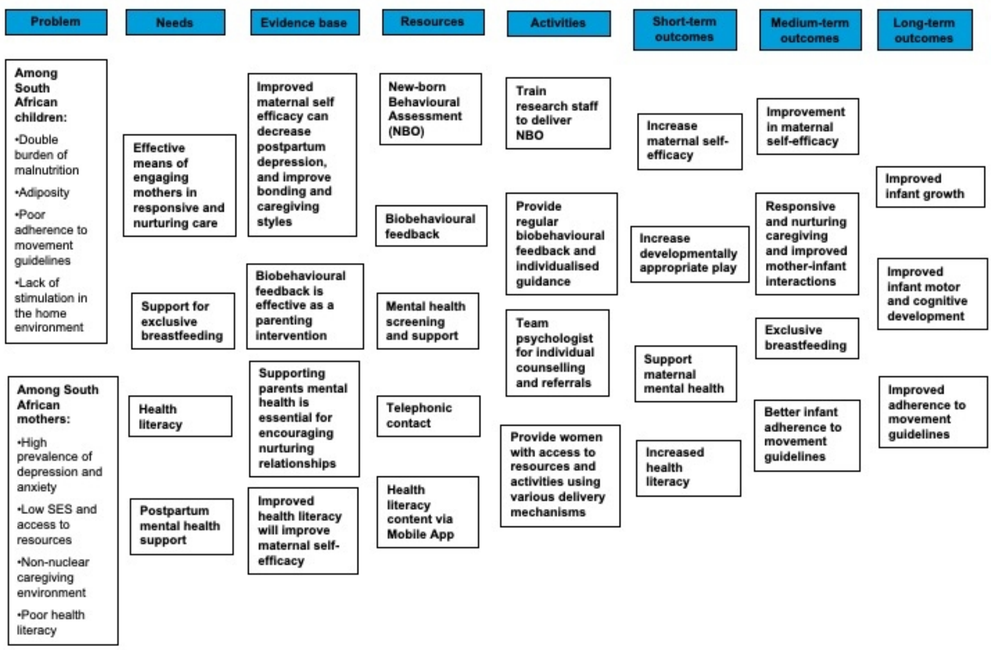

Figure 3. PLAY Study logic model

254x190mm (72 x 72 DPI)

Supplementary Figure 1. Sleep, screen time, routines and daily activities

Introductory open-ended conversation – how is mom? How is baby?

QUESTION 1: SLEEP

- How often does baby sleep and for how long (at night and naps)?
- What is the quality of baby's sleep? Does baby settle easily? Does baby wake up often?

Sleeps well

Difficulty with sleeping/ not sleeping enough

DISCUSSION:
What are healthy sleeping routines?

- Having same routine before bed
- Going to bed at similar time
- Reducing noise and light at bedtimes
- No background screens & recommendations for no

MATERIALS TO SEND:
- baby massage video
- examples of routine (bath, bottle, bed)

QUESTION 2: When baby isn't sleeping/ eating, what is she/ he doing?

DISCUSSION:

- developmental milestones e.g. rolling, sitting
- age and resource appropriate activities to do with baby
- how to encourage free play

MATERIALS TO SEND:

- Video on how to encourage motor development
- How to make musical instruments out of home materials
- Safety information on letting babies play freely without constraint

For peer review only - http://bmjopen.bmj.com/site/about/guidelines.xhtml

Supplementary Table 1. Data collection schedule for intervention group (green) and whole sample (blue)

**Timepoint (months)**	**0**	**4**	**6**	**8**	**12**	**16**	**20**	**24**
Intervention Activities								
Road to Health and Side by Side	✓							
New-born Behavioural Observation	✓							
Mental Health Screening	✓							
Biobehavioural Feedback		✓		✓	✓	✓	✓	✓
Accelerometery		✓		✓	✓	✓	✓	✓
Headcam		✓		✓	✓	✓	✓	✓
Ages and Stages		✓		✓	✓	✓	✓	✓
Screen time		✓		✓	✓	✓	✓	✓
Telephonic contact	✓	✓	✓	✓	✓✓	✓✓	✓✓	✓
Health literacy content	✓	✓	✓	✓	✓	✓	✓	✓
Infant measures								
Date of birth	✓							
Gestational Age	✓							
Sex	✓							
Weight and length	✓		✓		✓			✓
Head and mid-upper arm circumference	✓		✓		✓			✓
Health records	✓				✓			✓
Breastfeeding practices	✓	✓	✓	✓	✓			
Home Environment	✓				✓			✓
Accelerometery			✓		✓			✓
Headcam			✓		✓			✓
Ages and Stages			✓		✓			✓
Screen time			✓		✓			✓
Neurodevelopment (Intergrowth 21st package)								✓
Body composition (DXA)								✓
Maternal measures								
Socioeconomic status	✓				✓			✓
Mental Health	✓		✓		✓			✓
Social Support	✓		✓		✓			✓
Self-Efficacy	✓		✓		✓			✓
Health Literacy	✓		✓		✓			✓
Food Security	✓				✓			✓
Sleep and Activity	✓							

For peer review only

Supplementary Table 1. Data collection schedule for intervention group (green) and whole sample (blue)

General health	✓	✓	✓
Anthropometry	✓	✓	✓
Accelerometery		✓	✓
Headcam		✓	✓
Body composition (DXA)			✓

For peer review only

Professor and Head of School of Computer Science,

Electrical and

University of [illegible]

STANDARD PROTOCOL ITEMS: RECOMMENDATIONS FOR INTERVENTIONAL TRIALS

SPIRIT 2013 Checklist: Recommended items to address in a clinical trial protocol and related documents*

Section/item	Item No	Description	Page number
**Administrative information**			
Title	1	Descriptive title identifying the study design, population, interventions, and, if applicable, trial acronym	Title page
Trial registration	2a	Trial identifier and registry name. If not yet registered, name of intended registry	2
	2b	All items from the World Health Organization Trial Registration Data Set	
Protocol version	3	Date and version identifier	2
Funding	4	Sources and types of financial, material, and other support	14
Roles and responsibilities	5a	Names, affiliations, and roles of protocol contributors	1, 14
	5b	Name and contact information for the trial sponsor	
	5c	Role of study sponsor and funders, if any, in study design; collection, management, analysis, and interpretation of data; writing of the report; and the decision to submit the report for publication, including whether they will have ultimate authority over any of these activities	14

For peer review only

	5d	Composition, roles, and responsibilities of the coordinating centre, steering committee, endpoint adjudication committee, data management team, and other individuals or groups overseeing the trial, if applicable (see Item 21a for data monitoring committee)	
**Introduction**			
Background and rationale	6a	Description of research question and justification for undertaking the trial, including summary of relevant studies (published and unpublished) examining benefits and harms for each intervention	4-6
	6b	Explanation for choice of comparators	6-7
Objectives	7	Specific objectives or hypotheses	6-7
Trial design	8	Description of trial design including type of trial (eg, parallel group, crossover, factorial, single group), allocation ratio, and framework (eg, superiority, equivalence, noninferiority, exploratory)	7
**Methods: Participants, interventions, and outcomes**			
Study setting	9	Description of study settings (eg, community clinic, academic hospital) and list of countries where data will be collected. Reference to where list of study sites can be obtained	7
Eligibility criteria	10	Inclusion and exclusion criteria for participants. If applicable, eligibility criteria for study centres and individuals who will perform the interventions (eg, surgeons, psychotherapists)	7
Interventions	11a	Interventions for each group with sufficient detail to allow replication, including how and when they will be administered	7-10; Figure 1 and 2
	11b	Criteria for discontinuing or modifying allocated interventions for a given trial participant (eg, drug dose change in response to harms, participant request, or improving/worsening disease)	NA
	11c	Strategies to improve adherence to intervention protocols, and any procedures for monitoring adherence (eg, drug tablet return, laboratory tests)	11-12

For peer review only - http://bmjopen.bmj.com/site/about/guidelines.xhtml

	11d	Relevant concomitant care and interventions that are permitted or prohibited during the trial	7-10
Outcomes	12	Primary, secondary, and other outcomes, including the specific measurement variable (eg, systolic blood pressure), analysis metric (eg, change from baseline, final value, time to event), method of aggregation (eg, median, proportion), and time point for each outcome. Explanation of the clinical relevance of chosen efficacy and harm outcomes is strongly recommended	10-12; Figure 2
Participant timeline	13	Time schedule of enrolment, interventions (including any run-ins and washouts), assessments, and visits for participants. A schematic diagram is highly recommended (see Figure)	Table 1; Figure 2
Sample size	14	Estimated number of participants needed to achieve study objectives and how it was determined, including clinical and statistical assumptions supporting any sample size calculations	13
Recruitment	15	Strategies for achieving adequate participant enrolment to reach target sample size	7; 11; 13
**Methods: Assignment of interventions (for controlled trials)**			
Allocation:			
Sequence generation	16a	Method of generating the allocation sequence (eg, computer-generated random numbers), and list of any factors for stratification. To reduce predictability of a random sequence, details of any planned restriction (eg, blocking) should be provided in a separate document that is unavailable to those who enrol participants or assign interventions	7
Allocation concealment mechanism	16b	Mechanism of implementing the allocation sequence (eg, central telephone; sequentially numbered, opaque, sealed envelopes), describing any steps to conceal the sequence until interventions are assigned	7
Implementation	16c	Who will generate the allocation sequence, who will enrol participants, and who will assign participants to interventions	7

Blinding (masking)	17a	Who will be blinded after assignment to interventions (eg, trial participants, care providers, outcome assessors, data analysts), and how	7
	17b	If blinded, circumstances under which unblinding is permissible, and procedure for revealing a participant's allocated intervention during the trial	NA
**Methods: Data collection, management, and analysis**			
Data collection methods	18a	Plans for assessment and collection of outcome, baseline, and other trial data, including any related processes to promote data quality (eg, duplicate measurements, training of assessors) and a description of study instruments (eg, questionnaires, laboratory tests) along with their reliability and validity, if known. Reference to where data collection forms can be found, if not in the protocol	10-13
	18b	Plans to promote participant retention and complete follow-up, including list of any outcome data to be collected for participants who discontinue or deviate from intervention protocols	7; 11; 13
Data management	19	Plans for data entry, coding, security, and storage, including any related processes to promote data quality (eg, double data entry; range checks for data values). Reference to where details of data management procedures can be found, if not in the protocol	13-14
Statistical methods	20a	Statistical methods for analysing primary and secondary outcomes. Reference to where other details of the statistical analysis plan can be found, if not in the protocol	13; Figure 2
	20b	Methods for any additional analyses (eg, subgroup and adjusted analyses)	Figure 2
	20c	Definition of analysis population relating to protocol non-adherence (eg, as randomised analysis), and any statistical methods to handle missing data (eg, multiple imputation)	13
**Methods: Monitoring**			

Data monitoring	21a	Composition of data monitoring committee (DMC); summary of its role and reporting structure; statement of whether it is independent from the sponsor and competing interests; and reference to where further details about its charter can be found, if not in the protocol. Alternatively, an explanation of why a DMC is not needed	
	21b	Description of any interim analyses and stopping guidelines, including who will have access to these interim results and make the final decision to terminate the trial	Figure 2
Harms	22	Plans for collecting, assessing, reporting, and managing solicited and spontaneously reported adverse events and other unintended effects of trial interventions or trial conduct	11-12
Auditing	23	Frequency and procedures for auditing trial conduct, if any, and whether the process will be independent from investigators and the sponsor	
**Ethics and dissemination**			
Research ethics approval	24	Plans for seeking research ethics committee/institutional review board (REC/IRB) approval	20-21
Protocol amendments	25	Plans for communicating important protocol modifications (eg, changes to eligibility criteria, outcomes, analyses) to relevant parties (eg, investigators, REC/IRBs, trial participants, trial registries, journals, regulators)	2; 15
Consent or assent	26a	Who will obtain informed consent or assent from potential trial participants or authorised surrogates, and how (see Item 32)	14-15
	26b	Additional consent provisions for collection and use of participant data and biological specimens in ancillary studies, if applicable	
Confidentiality	27	How personal information about potential and enrolled participants will be collected, shared, and maintained in order to protect confidentiality before, during, and after the trial	7; 13-14

review only

For peer review only - http://bmjopen.bmj.com/site/about/guidelines.xhtml

Declaration of interests	28	Financial and other competing interests for principal investigators for the overall trial and each study site	14-15
Access to data	29	Statement of who will have access to the final trial dataset, and disclosure of contractual agreements that limit such access for investigators	14
Ancillary and post-trial care	30	Provisions, if any, for ancillary and post-trial care, and for compensation to those who suffer harm from trial participation	
Dissemination policy	31a	Plans for investigators and sponsor to communicate trial results to participants, healthcare professionals, the public, and other relevant groups (eg, via publication, reporting in results databases, or other data sharing arrangements), including any publication restrictions	
	31b	Authorship eligibility guidelines and any intended use of professional writers	14
	31c	Plans, if any, for granting public access to the full protocol, participant-level dataset, and statistical code	
**Appendices**			
Informed consent materials	32	Model consent form and other related documentation given to participants and authorised surrogates	Supplementary
Biological specimens	33	Plans for collection, laboratory evaluation, and storage of biological specimens for genetic or molecular analysis in the current trial and for future use in ancillary studies, if applicable	NA

*It is strongly recommended that this checklist be read in conjunction with the SPIRIT 2013 Explanation & Elaboration for important clarification on the items. Amendments to the protocol should be tracked and dated. The SPIRIT checklist is copyrighted by the SPIRIT Group under the Creative Commons "Attribution-NonCommercial-NoDerivs 3.0 Unported" license.

For peer review only - http://bmjopen.bmj.com/site/about/guidelines.xhtml

BMJ Open

The PLAY study: a randomised controlled trail to improve infant neurodevelopment by encouraging breastfeeding, play, and responsive caregiving using behavioural feedback

Journal:	*BMJ Open*
Manuscript ID	bmjopen-2022-064976.R1
Article Type:	Protocol
Date Submitted by the Author:	17-Nov-2022
Complete List of Authors:	Prioreschi, Alessandra; University of the Witwatersrand, SAMRC/Wits Developmental Pathways for Health Research Unit<br>Pearson, Rebecca; University of Bristol, Centre for Academic Mental Health, Addiction and Suicide Research, School of Social & Community Medicine; University of the Witwatersrand, SAMRC/Wits Developmental Pathways for Health Research Unit<br>Richter, Linda; University of the Witwatersrand, DSI-NRF Centre of Excellence in Child Development<br>Bennin, Fiona; University of the Witwatersrand, SAMRC/Wits Developmental Pathways for Health Research Unit<br>Theunissen, Helene; University of the Witwatersrand, SAMRC/Wits Developmental Pathways for Health Research Unit<br>Cantrell, Sarah J; University of the Witwatersrand, SAMRC/Wits Developmental Pathways for Health Research Unit<br>Maduna, Dumsile; University of the Witwatersrand, SAMRC/Wits Developmental Pathways for Health Research Unit<br>Lawlor, Deborah; Department of Social Medicine, University of Bristol, MRC Integrative Epidemiology Unit<br>Norris, Shane; University of the Witwatersrand Faculty of Health Sciences, SAMRC/Wits Developmental Pathways for Health Research Unit
**Primary Subject Heading**:	Public health
Secondary Subject Heading:	Global health, Mental health, Nutrition and metabolism, Paediatrics
Keywords:	Community child health < PAEDIATRICS, PUBLIC HEALTH, EPIDEMIOLOGY

SCHOLARONE™
Manuscripts

The PLAY study: a randomised controlled trail to improve infant neurodevelopment by encouraging breastfeeding, play, and responsive caregiving using behavioural feedback

Alessandra Prioreschi[1*], Rebecca Pearson[1,2], Linda Richter[3], Fiona Bennin[1], Helene Theunissen[1], Sarah J Cantrell[1], Dumsile Maduna[1], Deborah A Lawlor[4], Shane A Norris[1,5]

1. SAMRC/Wits Developmental Pathways for Health Research Unit, Department of Paediatrics and Child Health, Faculty of Health Sciences, University of the Witwatersrand, Johannesburg, South Africa
2. Centre for Academic Mental Health, School of Social and Community Medicine, University of Bristol, Bristol, UK.
3. DSI-NRF Centre of Excellence in Child Development, University of the Witwatersrand
4. MRC Integrative Epidemiology Unit at the University of Bristol, UK
5. School of Human Development and Health and NIHR Southampton Biomedical Research Centre, Global Health Research Institute, University of Southampton, Southampton, UK

* Corresponding Author:
Alessandra Prioreschi
Alessandra.prioreschi@wits.ac.za
SAMRC/Wits Developmental Pathways for Health Research Unit, Department of Paediatrics and Child Health, Faculty of Health Sciences, University of the Witwatersrand, Johannesburg, South Africa

For peer review only

Abstract

Introduction: The early infant caregiving environment is crucial in the formation of parent-child relationships, neurobehaviourial development, and thus child outcomes. This protocol describes the PLAY study, an intervention designed to promote infant neurodevelopment through encouraging responsive parenting using biobehavioral feedback, and supportive interventions.

Methods and analysis: 210 mother-infant pairs will be recruited at delivery from public health facilities in Soweto, South Africa, and individually randomised into two groups. The trial will consist of a standard of care arm and an intervention arm. The intervention will start at birth end at 12 months, and outcome assessments will be made when the infants are 0, 6, and 12-months of age. The intervention will be delivered by community health helpers (HHs) using an app with resource material, telephone calls, in person visits, and behavioural feedback with individualized support. Every 4-months mothers in the intervention group will receive rapid feedback via the app on their infant's movement behaviours, growth, developmental milestones, and on their interaction styles with their infant. At recruitment, and again at 6 months, mothers will be screened for mental health risk and women who score in the high-risk category will receive an individual counselling session from a licensed psychologist, followed by referral and continued support as necessary. The primary outcome is efficacy of the intervention in improving infant neurodevelopment at 12 months, and the secondary outcomes are feasibility and acceptability of each component of the intervention.

Ethics and dissemination: The PLAY study has ethical approval from the Human Research Ethics Committee of the University of the Witwatersrand (M220217)

Trial Registration: This trial was registered with the Pan African Clinical Trials Registry (https://pactr.samrc.ac.za) on 10/02/2022 (identifier: PACTR202202747620052)

Protocol version: 02/11/2022 (version #1.8)

Key words: behavioural feedback, play, early child development, responsive caregiving

Strengths and limitations of the study:

- The PLAY study uses behavioural feedback linked with personalised guidance to improve and individualise intervention content and implementation
- The PLAY study will be guided by an advisory group of community members (CAG) consisting of women with infants and their mothers/influential family matriarchs, and consultation with this advisory group will happen

For peer review only - http://bmjopen.bmj.com/site/about/guidelines.xhtml

before and during the intervention to ensure we remain contextually aware in creating and delivering our study content

- The PLAY study relies to some extent on the availability of cell phone or computer access for the delivery of intervention content, which may be limiting in the Soweto setting.
- The COVID pandemic may interfere with our recruitment and intervention strategies, and we have made the study less reliant on face-to-face interactions to accommodate any further lockdown restrictions in South Africa.
- The PLAY study uses advanced and robust measurement tools, which in some cases are novel in South Africa and/or globally, thus providing reliable evidence which can inform future research.

For peer review only

Background

Over the last decade, emphasis globally is being placed on improving early childhood development (ECD) [1]. Estimates are that 250 million children (43%) under the age of five living in low-and-middle-income countries (LMICs) are at risk of not meeting their developmental potential [2]. Furthermore, a double burden of malnutrition persists in LMICs, with wasting present in 6.4% and overweight present in 6% of children under age five [3]. While optimising growth and development requires a life course approach, the first two years are key in optimising developmental potential [1 4]. This highly sensitive period (the first 1000 days of life) is an experience-dependent and –expectant process which is plastic and malleable [5]. The World Health Organisation (WHO) has recently published four key guidelines for improving ECD [6]: integration of nutrition and caregiving interventions, promotion of early learning, supporting maternal mental health, and responsive caregiving.

Integrating nutrition into caregiving interventions

In the first 6 months of an infant's life, exclusive breastfeeding is regarded as the optimal and most nutritionally complete form of infant nutrition [7 8]. Research indicates that infants who are breastfed for longer may have higher cognitive function, and lower infectious morbidity and mortality. Breastfeeding may also protect against diabetes, cardiometabolic disease and overweight later in life [7]. It is estimated that improving global breastfeeding rates alone could potentially, prevent more than 800,000-child deaths annually [7]. Despite the widely established benefits of breastfeeding, in the South African context, roughly 67% of infants initiated breastfeeding within the first hour of birth in 2016, yet only 32% of children under the age of 6 months remain exclusively breastfed. The mean duration of exclusive breastfeeding was found to be only 2.9 months [9], with widespread introduction of solid foods and non-breastmilk liquids before 3 months [10 11]. Breastfeeding self-efficacy has been found to directly influence breastfeeding practices, and high breastfeeding self-efficacy was found to play a critical role in exclusive breastfeeding duration in the South African setting [12].

Qualitative findings from urban South Africa reported that infant nutrition interventions in this context should focus on: fostering maternal self-efficacy around exclusive breast-feeding; challenging mixed feeding practices and encouraging more responsive feeding approaches; and engaging family members to promote supportive household and community structures around infant feeding [13].

Promotion of early learning through play

Infant movement behaviours have been identified as important for early childhood growth and development [14]. Three categories of movement behaviours are of key importance: physical activity, sleep, and sedentary behaviours [14]. These three behaviours are contingent on each other to make up a 24-hour day, yet have independent health implications and thus

For peer review only - http://bmjopen.bmj.com/site/about/guidelines.xhtml

independent recommendations, which should all be met for optimal health. Guidelines for these behaviours in the first few years of life encourage tummy time and active play, sufficient and regular sleep, and minimisation of activities that require infants to be restrained (eg: car seats, strollers, walkers) [14-16]. Play is defined as intrinsically motivated activities done for recreational purposes and enjoyment. Play is how children learn, explore, engage in physical activity and motor development, develop socially, emotionally and cognitively, and bond with caregivers [17 18]. Outdoor play is particularly encouraged due to its beneficial effects on both physical activity and development [19]. Additionally, interactive and stimulating play (such as reading, singing, and playing with other adults or children) is encouraged to promote development [20], and also supports early learning [6].

Formative research in South Africa has determined that higher intensity movement was associated with earlier attainment of developmental milestones [21], and with taller stature and lower abdominal adiposity [22]. However, the majority of infants in South Africa were not meeting recommended 24-hour movement guidelines [23]. When using synchronous 24-hour accelerometery worn by mothers and infants, we showed that increased physical activity among mothers while caring for their infants was associated with increased infant movement during the same period of time [24]. This highlights the important role of the mother in infant movement behaviours, and thus growth and development. Interactive play is particularly important for promotion of parent-child bonding, offering an ideal opportunity for parents to engage with their children through stimulating activities which encourage movement, and in turn promote cognitive, emotional and social development. Stimulation interventions in LMICs have been shown to improve home caregiving environments, mother-child interactions, and maternal health literacy around ECD [25].

Qualitative research examining maternal perceptions of the importance of play in infancy reported that mothers in South Africa perceive developmental attainment as a key child outcome, indicating that interventions in this context should focus on promoting child development [26]. Mothers also reported significant barriers to encouraging play; including financial constraints, safety concerns and lack of certainty about how to promote play. Importantly, they did not perceive infant movement behaviours as behaviours that could be, or needed to be changed [26]. Additionally and similar to findings from national reports, mothers reported not reading to their children, and most children in this context did not attend creche, indicating the importance of intervening in the home environment to improve opportunities for early learning [26 27].

Supporting maternal mental health

In South Africa, up to 57% of mothers present with symptoms of postnatal depression [28]. Qualitative studies have shown that in Soweto, household centred issues impacted upon the amount of chronic anger, stress, depression and suicidal ideation as well as material and relational hardships experienced by women [29]. Relational stress is exacerbated by the fact that mothers and children in South Africa live in multiple-generational households (66%), while only 21% are said to live in

For peer review only - http://bmjopen.bmj.com/site/about/guidelines.xhtml

households that may be defined as nuclear [27]. Women in Soweto are also exposed to high unemployment rates, low salaries, rife domestic violence [28], and thus social vulnerability [30].

Infants of mothers with depressive symptoms have an increased chance of developmental delay in the first 12 months of life [31]. In South Africa, a significant increase in psychological challenges at two years of age was evident when maternal postpartum depression was present [31]. Additionally, within the context of high exposure to trauma in South Africa, post-traumatic stress disorder in mothers was significantly associated with poorer infant developmental outcomes in fine motor and adaptive behaviour, as well as emotional regulation [32 33]. Due to the high prevalence of poor postpartum mental health in South Africa, and lack of support available for these women, screening of mothers for postpartum depression, anxiety, and post-traumatic stress disorder is recommended [28].

Responsive caregiving

Responsive caregiving is essential in helping children to thrive. Responsive caregiving refers to the encouragement of caregivers to make eye contact, to smile, cuddle and praise infants. Responsive parenting urges caregivers to notice their children's cues of hunger, illness, emotional distress, and signs of pleasure; and to respond sensitively and appropriately [20], while also allowing the child to independently explore and self-regulate. Responsive caregiving is therefore important for ensuring better nutrition and feeding practices, health practices, and neurodevelopment. Responsive caregiving ultimately leads to improved bonding between mother and infant. In a well bonded relationship, the mother aims to meet her infant's needs and the infant responds to having its needs met. Bonding serves to generate the feeling of connectedness between the dyad [34], and is crucial for developing a secure infant attachment.

A recent systematic review and meta-analysis of parenting interventions to improve ECD in children under the age of three has shown that interventions with responsive caregiving components had greater effects on child cognitive development, as well as on parenting practices and parent-child interactions [35]. The authors recommended that future interventions should aim to directly support parent responsiveness and sensitivity.

Tools for supporting parents in promoting these behaviours

The early infant caregiving environment is thus crucial in the formation of parent-child relationships, mother-infant synchronicity and contingency, behaviour development, and therefore child outcomes [20 36 37]. Mothers who are supported in their transition to parenthood have higher self-efficacy, and are less likely to develop past-partum depression [35 38]. Infants

For peer review only - http://bmjopen.bmj.com/site/about/guidelines.xhtml

who have confident and healthy mothers are more likely to develop resilience and emotional and cognitive competencies, to have healthy growth trajectories, and to develop healthy behavioural patterns [39].

Maternal self-efficacy is a likely contributor to responsiveness and sensitivity, and is determined by a mother's belief in her ability to care for her child, her emotional and psychological health, her child's temperament and health, and various environmental factors. Strategies to improve self-efficacy in parenthood include improving health literacy, reminding mothers of their natural ability to care for their child, social support, and promotion of mental health. Interventions that are targeted and focus specifically on changing maternal behaviour are most effective [40]. Maternal mental health should be supported through integration into ECD interventions and programmes, yet a lack of studies on maternal mental health that report on child health and development outcomes, in addition to mental health outcomes, has been identified as a critical gap [6]. Other gaps include identifying the most effective responsive caregiving interventions in LMICs, combining nutrition and caregiving strategies [6], and identifying ways to improve parenting behaviours without undermining self-efficacy.

In the first 3-months postpartum, tools to sensitise mothers to infant's competencies and to promote positive and nurturing interactions between caregivers and infants are successful in reducing postpartum depression, and increasing self-efficacy in both parents and health care providers [16 41]. Following early supportive intervention and sensitisation to infant behavioural cues, mothers benefit from continued support around breastfeeding, and promotion of healthy growth and development [16]. This can include provision of content to improve health literacy, practical tools and activities that promote infant development, continued sensitisation to infant behaviours and mother-infant interaction styles, and mental health promotion.

Behavioural feedback is an important and effective component of parenting interventions and behaviour change interventions in general [42], which can help to enhance parenting skills and thus child outcomes [41 43]. Feedback consists of comparing individual behaviour to a standard, and is thought to change behaviour by changing the individual's locus of control and focusing attention on a particular behaviour thus regulating that behaviour [44-46]. Feedback on parenting behaviours can be directive and/or responsive to behaviours in real-time, or following review of video recorded interactions [43 47]; and encouraging parents to practice skills following feedback is one of the strongest and most consistent predictors of improved parenting outcomes in meta-analyses [40 41]. Additionally, tailoring behavioural feedback is particularly effective for behaviour change in general [48].

Aims

1. To test the efficacy, feasibility, and acceptability of an intervention designed to promote neurodevelopment through encouraging responsive parenting using behavioral feedback, content provision and mental health support.

For peer review only - http://bmjopen.bmj.com/site/about/guidelines.xhtml

2. To test the efficacy, feasibility, and acceptability of providing content, support, and referrals to promote breastfeeding self-efficacy
3. To test the efficacy, feasibility, and acceptability of providing content, and increasing opportunities for early learning through play to promote health literacy

Hypotheses

1. Using behavioural feedback and content provision to support mothers in their ability to be responsive, promote early learning in the home, understand and engage with their infants' health behaviours (specifically movement behaviours interactions), in conjunction with supporting mothers' mental health, will result in better infant neurodevelopment in the first two years of life.
2. Supporting mothers with breastfeeding content, guidance, and referrals to lactation specialists in the first 6 months postpartum will increase self-efficacy and thus improve breastfeeding exclusivity
3. Proving mothers with access to resources, content, and activities to support their infant's play and development will increase their health literacy and thus encourage play and promote development

Methods

The Play Love And You (PLAY) study was developed with the overriding theme that all an infant really needs are opportunities for movement and development (play), responsive and interactive caregiving (love), and the mother herself (you).

Study design

The PLAY study was designed following the Standard Protocol Items (Recommendations for Interventional Trials (SPIRIT) 2013 statement) [49]. Mother-infant pairs (n=210) will be recruited starting in the third quarter of 2022, and individually randomised into two groups (total n after expected attrition =150, n=75 in each group) using simple randomization generated by Stata V.17. The intervention will last from birth to 12 months, with main assessments made when the infants are 0-, 6-, and 12-months of age. The trial will consist of a standard of care arm and an intervention arm – see Figure 1. Data collection teams will be blinded to randomisation at baseline and during primary outcome assessments, and separate data collection teams will collect baseline compared to primary outcome data. Blinding will be maintained by the data manager and broken only for *a priori* defined analyses (Figure 2). Randomisation into either group will be generated by the data manager only after recruitment and baseline assessments are completed, whereafter participants will be assigned to the intervention delivery teams accordingly (intervention delivery teams will thus not be blinded). Intervention delivery teams will work separately and independently of each other, and of data collection teams.

Participants

For peer review only - http://bmjopen.bmj.com/site/about/guidelines.xhtml

We will recruit 210 mother-infant pairs within 3-10 days following delivery at community clinics in Soweto. Soweto is an urban-poor area in the City of Johannesburg covering 200 km^2 with over 1.3 million people (6400/km^2). Mother-infant pairs will be eligible to participate if:

- Mothers agree to participate for the duration of the study
- They reside in Soweto and plan to remain in Soweto for the study duration
- The mother is aged >=18 years
- The mother is the primary caregiver

Trial Intervention

Standard of care arm

Mothers assigned to the standard of care arm will receive the Road to Health booklet as well as the Side-by-Side services provided by the National Department of Health as routine postpartum care. This content is divided into 5 knowledge pillars (nutrition, love, protection, healthcare, and extra care), and functions to record child's growth and health interventions, provide information for caregivers, and encourage collaboration between healthcare workers and caregivers (https://sidebyside.co.za).

Additionally, they will be contacted telephonically or in person every 2 months to check in, and to provide referrals to health care facilities and community services if necessary; and will have anthropometric, development, movement and interaction measurements taken every 4 months.

The standard of care arm will therefore include:

- Road to Health booklet as well as the Side-by-Side services provided as routine care postpartum at delivery
- Telephonic or in person check in every 2 months, and referrals to health care facilities and community services if necessary
- Measurements taken every 4 months.

Intervention arm

Mothers assigned to the intervention arm will receive everything as per the standard of care arm. Additionally, they will receive various micro-interventions according to the following key intervention themes: 1) exclusive breastfeeding for 6-months, 2) interactive play and promotion of early learning, 3) responsive caregiving, and 4) maternal mental health. Each intervention theme will be prioritized during different phases of the trial and supported by components from the other themes. Figure 2 details when each theme will be prioritized and which supporting interventions will be included. Interventions will be delivered telephonically or in person, and via a mobile App created for the purposes of this trial. Interventions are detailed below:

For peer review only - http://bmjopen.bmj.com/site/about/guidelines.xhtml

Behavioral feedback

At recruitment, mothers will have the Newborn Behavioural Assessment (NBO)[50] administered by trained research staff. The NBO consists of 18 neurobehavioral observations along four dimensions — autonomic, motor, state organization and attentional-interactional - ultimately yielding a profile of the infant's behavioural repertoire. Importantly, the NBO allows for provision of infant specific anticipatory guidance, thus engaging parents in discussions around nurturing care, mother-infant interaction, play and development, and breastfeeding.

Every 4-months mothers will receive rapid feedback via the PLAY Study App on their infant's and their own movement behaviours, growth, developmental milestones, and on their interaction styles with their infant. Feedback will be based on maternal reported milestone attainment and anthropometric measures compared with age-appropriate standards, and objective measurement of infant movement behaviours and *headcam* video footage of interactive play in the home environment. Feedback on movement behaviours will consist of a graphical representation of the infant's/mother's movement behaviours over the previous week, with personalised information and advice about: sleep routines, nap times, daily activity routines, peaks of high activity vs lulls in activity, correlation of feeding routines with activity patterns, and correlations between sleep duration and the next day's activity. An example of the graphical feedback presented is included in Supplementary Figure 1. Feedback on mother-infant interactions during play is guided by the VIPP-SD[51] and will consist of a video segment from a peak interaction moment with mother and infant perspectives presented side-by-side, with personalised information and guidance on: positive interactions at 4 months (baby smiling, eye contact, soothing, directing attention, reinforcing mothers strength, communication and bonding); perspective taking at 8 months (describing and narrating the interaction, seeing baby as an individual, understanding baby cues), and recognising child feedback at 12 months (sensitivity and responsiveness, following cues, adjusting behaviour, soothing styles, interaction chains). The App will contain the graphical and video feedback, and will also allow mothers to observe changes in their infant's behaviour and development over the trial period.

Mental health screening and support

At recruitment, and at 6 months mothers will complete the Edinburgh Postnatal Depression Screening questionnaire (EPDS), the Post-traumatic stress syndrome Checklist 5 (PCL-5), the Adverse Childhood Experiences Scale (ACES), and the Antenatal Stress questionnaire (ASQ). Women who score in the high-risk category on one or more of these questionnaires will receive a phone call to assess any immediate risk, and an individual counselling session from a licensed psychologist, followed by referral and continued support as necessary. Women who score at medium risk on one or more of these questionnaires will receive referrals from the licensed psychologist and continued support as necessary, and uptake of this support will be monitored. All participants will receive a flyer on mental health and available services and support upon completion of the questionnaires.

For peer review only - http://bmjopen.bmj.com/site/about/guidelines.xhtml

Health Literacy content delivered via the PLAY Study mobile App

Every week, health theme specific literacy content will be delivered via the PLAY Study mobile App, which was designed for the purposes of this intervention. The App creates a password protected profile for each participant and houses a section to deliver the feedback when available, as well as the weekly (and historic) content. This content is loaded to the profile every Monday, and participants can choose to receive push notifications to alert them of new content. Participants can engage with the App at any time, for as long as they wish, and as many times as they wish during the intervention. The weekly content will appear in the format of quick and easy tips, short videos, infographics, or ideas for home-based activities. Mothers will have continuous access to the App with all relevant resources, activities, and tools to support their interactions with their infant.

Health Literacy content delivered telephonically or in person

Every two months theme focused content will be delivered telephonically or in person. Telephonic or in person visits will also follow the key intervention themes schedule; but will allow mothers to guide the conversation by using open questions and providing anticipatory guidance. Referrals to lactation specialists or psychologists or other services will be provided as necessary.

Health literacy content themes will be scheduled as follows and according to Figure 2.

- From 0-6 months content will be designed to increase breastfeeding self-efficacy and therefore promote exclusive breastfeeding
- From 6-12 months content will be designed to increase health literacy about play and development by providing content and activities to support and therefore improve these outcomes
- From 0-12 months there will be additional content to support maternal mental health, and encourage responsive caregiving and bonding.

All content will be guided by a broad range of pre-existing content such as UNICEF resources (https://www.unicef.org), and WHO recommendations [6], and other evidence based online resources. Contextually relevant material or adaptations to existing content will also be co-developed with a community advisory group (CAG).

The intervention arm will thus include the standard of care activities and:

- NBO administered at recruitment with feedback and anticipatory guidance
- Rapid behavioral feedback with individualized guidance on infant's development, growth and movement behaviours, and mother-infant interaction styles
- Mental health screening with referrals and individual support as needed for high-risk mothers

For peer review only - http://bmjopen.bmj.com/site/about/guidelines.xhtml

- Health literacy content
    - Weekly theme focused content delivered via the App
    - Telephonic or in person conversations with discussions around key themes every two months and referrals as necessary

Intervention Delivery

The intervention has been informed by the UK MRC Guidelines for Complex Interventions, and is grounded in behaviour change techniques developed by Abraham and Michie [42]. We used the TIDieR (Template for Intervention Description and Replication) Checklist. Figure 3 details the logic model to illustrate the pathways to impact that guided the intervention approach. The agents of change are community health helpers (HHs), who do not need to have any specific qualification or expertise in health promotion, but will be trained by the study team in community health promotion, as well as relevant intervention methodology. The HHs are supported by a supervisor with access to a referral network.

The intervention will be delivered by a combination of phone calls, in person visits, app-based health literacy content, personalised behavioural feedback and guidance; as well as individual counselling sessions for high-risk mothers. The content delivery will be guided by a schedule following key themes according to infant age and developmental stage. Phone calls and in person visits will be guided by a conversation flow chart, which will start with HHs asking mothers how they are doing and gently introducing the theme for discussion using open-ended questions. The flow chart will be designed to allow HHs to either ask follow up questions, or to provide anticipatory guidance and direct them to relevant resources in the App based on mothers' individual responses (example provided in Supplementary Figure 2). This will allow mothers to guide the conversation, while still following key themes for discussion. HHs will refer mothers as necessary if any concerns are raised during these conversations.

Outcome measures

Data collection will be conducted by independent, trained research assistants. The primary outcome measure is efficacy of the intervention in improving infant neurodevelopment. Secondary outcomes will assess feasibility and acceptability of the intervention at 6-, and 12-months, as well as interim outcomes (Figure 2).

<u>Primary outcome at 12 months:</u>

- Infant neurodevelopment compared between the intervention and control groups using the cognitive domain of the Bayley Scales of Infant and Toddler Development Third edition (Bayley-III)

<u>Secondary outcome at 6 months:</u>

- Feasibility and Acceptability of the breastfeeding intervention

For peer review only - http://bmjopen.bmj.com/site/about/guidelines.xhtml

- Breastfeeding self-efficacy from 0-6 months compared between intervention and control groups
- Exclusive breastfeeding from 0-6 months compared between intervention and control groups

Secondary outcomes at 12 months:

- Feasibility and Acceptability of the health literacy and behavioural feedback intervention
- Individual domains of infant neurodevelopment compared between intervention and control groups
- Change in infant weight to length ratio compared between intervention and control groups
- Infant fat mass index (assessed using DXA fat mass divided by length) compared between intervention and control groups
- Change in maternal health literacy from 6-12 months compared between intervention and control groups
- Change in maternal mental health from 0-12 months compared between the intervention and control groups
- Infant movement behaviours compared between intervention and control groups
- Mother-infant interaction styles compared between intervention and control groups

Data Collection

Primary outcome measure:

Infant neurodevelopment will be assessed at 12 months using the cognitive domain of the Bayley Scales of Infant and Toddler Development Third edition (Bayley-III). This package is a gold standard series of behavioural assessments used by clinicians and researchers to assess the developmental functioning of young children across various domains including cognitive, language, motor and socioemotional development, and adaptive behaviour [36]. This tool has been successfully utilised in various LMICs, including South Africa [37 38].

Secondary outcome measures:

Acceptability of each phase of the intervention will be assessed based on the Theoretical Framework of Acceptability [52]. This framework includes the following criteria: Affective Attitude, Burden, Ethicality, Intervention coherence, Opportunity costs, Perceived Effectiveness, and Self-Efficacy. These criteria will be assessed by means of focus group discussions (FGDs), and questionnaires specific to each intervention theme.

Feasibility of each phase of the intervention will be assessed by determining whether the developed intervention is implementable in this context, has potential to work, as well as how it could be improved for future use and in other settings.

For peer review only - http://bmjopen.bmj.com/site/about/guidelines.xhtml

We will follow the MRC guidance on process evaluation of complex interventions [53]. Evaluation will focus on implementation, mechanisms of impact, and context.

- Compliance will be assessed by counts (e.g.: login frequency on app, delivery of messages, and monitoring of phone calls using a log), and by discussions with the HHs.
- Intervention dose will be monitored by recording the frequency and duration of contact as well as the topics of conversation between participants and HHs or feedback given to participants using the logs.
- To understand the mechanisms through which the intervention brings about change in specified outcomes, women's experiences of contact with HHs and the App will be assessed using interviews and focus groups at the close of each intervention phase. We will also use the secondary outcomes (process data) to determine whether individual proposed pathways of impact are effective prior to analysing trial outcomes.
- In considering the context, identification of factors that might act as barriers or facilitators to intervention implementation or effects will be assessed. This will include local and national policy, local service configuration and provision, and socio-demographic and environmental factors of the participants. Our approach to assessing context will be through a combination of policy mapping, quantitative data collection, and longitudinal consultation with the community advisory group.

Maternal breastfeeding self-efficacy will be assessed every two months from 0-6 months using the breastfeeding self-efficacy short form (BSES-SF) [54]. This comprises of 14 questions, each with a five-point Likert scale response scale ranging from 1=not at all confident to 5=very confident. The higher the final aggregate score (which has a range of 14 to 70) the higher the level of breastfeeding self-efficacy.

Exclusive breastfeeding will be assessed every two months form 0-6 months by asking mothers whether they are currently breastfeeding and if not, at what infant age did they stop breastfeeding and why they stopped breastfeeding. They will also be asked if they are giving their infant anything other than breastmilk, and if so, why.

Weight and length will be measured by trained research staff at baseline and 12-months. Weight:length ratio (WLR; kg/m) will then be calculated to estimate anthropometric changes in body composition. At 12-months, Dual X-Ray Absorptiometry (DXA) scans will be performed by a trained technician. Whole-body measurements of fat mass and fat-free mass will be extracted for use in analyses. Fat mass index (FMI; kg/m^3) will be calculated from these estimates to describe adiposity.

Health literacy will be assessed at 6- and 12-months using the parent version of the Health Literacy Questionnaire (HLQ) [55], which covers nine areas of health literacy including functional health literacy and has been validated in high- and low-to-middle-income countries.

For peer review only - http://bmjopen.bmj.com/site/about/guidelines.xhtml

Various aspects of maternal mental health will be assessed at baseline, 6- and 12-months. The Post-traumatic Stress Disorder Checklist (PCL-5) [56] will be administered whereby rating scale descriptors include: "Not at all," "A little bit," Moderately," "Quite a bit," and "Extremely." A total symptom severity score is calculated by adding up the item scores which range from 0–80. The cut-off for PTSD that will be used in this study was 31. The Edinburgh Postnatal Depression screen (EPDS) [57] will be used to detect postnatal depression. Scores for each item range from 0 to 3 on a Likert scale, and are added together to obtain an overall total. Scores of 7-13 indicate mild depression, 14-18 moderate and 19-30 severe depression. The Adverse Childhood Experience questionnaire (ACE) [58] will assess childhood abuse, neglect, and household challenges. A score from 0- 10 is calculated based on how many types of events were experienced before 18 years old. The Antenatal Stress Questionnaire (ASQ) will be administered to assess stressful life events. An additional nine items about social support will be included. The Postpartum Bonding Questionnaire (PBQ) [59] will be used to detect early mother–infant bonding. It consists of 4 subscales "impaired mother/infant bonding", "rejection and anger", "anxiety about care", and the "risk of abuse." These questions are rated on a 6-point Likert scale, ranging from 0 (always) to 5 (never).

Infant adherence to movement guidelines will be assessed every 4 months using accelerometery. Seven-day, 24-hour wrist worn accelerometery data (Axivity-AX3 worn on both mother and infant) will be collected, downloaded (omgui, Open Movement, UK), auto-calibrated, and processed. Data is summarised to generate average acceleration (mg) per 5-second interval, as well as overall time (hourly and weekly) spent in varying intensities of activity, which will allow for determination of adherence to movement guidelines, and changes in activity levels over the intervention period.

Mother-infant interactions will be measured during play using first person observation and behavioural analysis every 4 months. Mother-infant interactions have primarily been measured using questionnaires or third party observation, yet these measures are limited by their subjectivity and intrusiveness. First-person observation using *headcams* – small cameras which are attached to headbands on both infants and mothers for a period of time while interacting at home – provide a first person view of the interaction from both infant and mother perspectives with more naturalistic and detailed measures. These *headcams* have been piloted in South Africa, and were found to be largely acceptable and feasible in this context [36]. *Headcam* measures will be collected during at least five, 3-5 minute play interactions over the course of a week. *Headcam* observations will be coded using Noldus Observer XT software. An event-based coding framework will be used to categorise second-by-second key maternal and infant behaviours, i.e.: touch, play, vocalisations, and gaze. This allows for determination of the probability of certain behaviour preceding or following infant play. By combining measures of movement behaviours and

For peer review only - http://bmjopen.bmj.com/site/about/guidelines.xhtml

mother-infant interactions; intensity and frequency of movement during interactive play, as well as interaction styles during play will be determined for feedback to mothers.

Patient and Public Involvement

Prior to trial implementation, a community advisory group (CAG) (n=16) of mothers from Soweto with infants aged 0-3 months will be established to serve as experts on the lived experience of being a mother to an infant in Soweto. For about half of these women, family matriarchs will be recruited to form a separate CAG (n=8). Research from this context has indicated that family matriarchs play an important role in the choices that mothers make around infant feeding and nutrition, and as such it is pertinent they be included in interventions targeting breastfeeding and other child care practices.

Intervention content and activities, and behavioural feedback will be tested and/or co-developed with the CAG, using focus group discussions and piloting of intervention content and feedback. Thus, acceptability and feasibility of the intervention content will be tested by the CAG in order to understand any changes required and thus refine the intervention accordingly.

The CAG will continue to advise throughout the duration of the intervention, via regular (every 4 months) focus group discussions (FGDs) where the research team will raise queries about intervention content, delivery and uptake, retention strategies, and feedback mechanisms. Additionally, the CAG will provide important insight into their lived experience of being a mother in Soweto over time and as their infant grows. This will provide useful Insight to guide intervention conversations, and to better understand the context within which the intervention is being delivered.

Monitoring

Monitoring of intervention activities will be done throughout the trial. The delivery of each contact (duration, date, content) will be recorded using a monitoring log in order to be able to monitor intervention intensity and frequency. Engagement with the App content and behavioural feedback (i.e.: number of page visits per day, and duration of time spent on the App) will be monitored via the App.

Adherence will be assessed every 4 months, and strategies to improve adherence will be discussed with the CAG as necessary. Additionally, the research team will meet with HHs weekly to determine any concerns or issues with compliance or retention, and strategies will be discussed accordingly. Any adverse events will be recorded and reported as necessary according to standard operating procedures.

Sample size

For peer review only - http://bmjopen.bmj.com/site/about/guidelines.xhtml

Given expected attrition of 30%, recruiting 210 mother-infant pairs should yield a final sample size of 150 mother-infant pairs, which will be divided into the two groups (n=75 each). A sample size of 150 mother-infant pairs divided into two groups (n=75 each; control and trial group) will allow for 80% power to detect efficacy of the intervention based on a 0.2SD change in the cognitive subscale of the Bayley Scale in each group with an alpha of 0.05 using published data from infants in South Africa (mean(SD) of 99.7(15.8)) [60].

Attrition will be minimised by maintaining regular contact with both groups, by recruiting participants who plan on remaining in Soweto, and by using a flexible and versatile delivery approach including in-person visits, app based content, telephonic sessions, mobile messaging, and site visits.

Data analysis plan

The efficacy of the intervention will be assessed using intention to treat analysis. Secondary outcome data will be compared between the intervention and standard of care arms using Student's unpaired t-tests. Focus will be placed on the primary efficacy outcome (neurodevelopment) due to limited power for secondary outcomes, which will therefore be treated with caution and presented using point estimates and precision (95% CI). Sensitivity analyses will include comparing baseline characteristics between intervention and standard of care arms, examining the effect of intervention dose on outcome data, and stratifying analyses by sex.

The feasibility and acceptability of the intervention at each phase will be assessed using qualitative and quantitative methods. FGDs will be transcribed and coded line by line, and data will be grouped according to common themes. Themes will be sorted into sub-themes and continually reviewed to identify an overarching narrative to identify key findings from each FGD.

Implications and future directions

If found to be feasible, acceptable and successful, these findings could lead to implementation of the intervention on a larger scale. Particularly, implementing intervention content within primary health care services, and linkage with existing digital solutions (like MomConnect in South Africa), would allow for dissemination of the health literacy components of this intervention nationally. Furthermore, the PLAY Study App has the potential to be developed further and made into an accessible App for all mothers. All of this content aligns with the Nurturing Care Framework and WHO guidelines and is therefore relevant at a national policy level, as well as internationally. Additionally, engagement with the community and dissemination of findings to participants and other stakeholders provides opportunities for knowledge mobilisation and therefore greater impact. There is potential to transfer the intervention to other LMICs and HICs, however since this content was designed with the community to be contextually relevant, adjustments would need to be made to ensure feasibility and

For peer review only

acceptability in other contexts. Certain aspects of the intervention, such as the accelerometry and headcam assessments and feedback are more complex and would require funding and training to implement on a larger scale, yet the concept of providing feedback using some of the other assessments such as development and growth can feasibly be incorporated into digital platforms or the PLAY Study App for scaling up.

Limitations

The PLAY study relies to some extent on the availability of cell phone or computer access for the delivery of intervention content, which may be limiting in the Soweto setting. However, we will attempt to mitigate this by making the app data free, by making the app available on multiple platforms (web, mobile, android, Apple etc), and by continuously consulting with the CAG to ensure we come up with appropriate solutions if we encounter lack of access. Additionally, the intervention content and delivery was designed with the Soweto community to be feasible and acceptable in a South African context, and adaptations would need to be made to ensure the same in other settings. Findings from this study are relevant to a socioeconomically disadvantaged group of mothers, and thus cannot be extrapolated to higher income settings or different population groups. This study does not specifically include fathers due to the low prevalence of caregiving fathers in this context. Future studies directed at both parents or at other caregivers would need to be adjusted for those population groups.

Data management

REDCap will be used for data management (https://www.project-redcap.org). Secure Sockets Layer (SSL) provides the needed security for the REDCap similar to what a VPN will provide. The Wits University server hosting REDCap is resilient and hardened against hacking and other forms of attacks. All data traffic is encrypted, and an installed network firewall provides secure network access. The REDCap system provides inherent application-level security including access controls and comprehensive audit trails; and all incoming requests get intentionally filtered, sanitized and escaped to prevent against attacks.

Data files and metadata will be uploaded and stored on REDCap by the project data manager, on a password protected Wits server for the duration of the project to enable team access. REDCap is used for data and metadata storage, as well as for data collection. Data will be inputted online with secure web authentication, data logging and SSL encryption. Accelerometery files and video files, as well as focus group discussion notes and transcripts will be stored on a secure and encrypted laptop, as well as on an external hard drive, and backed up as meta-data to REDCap. Regular back-up copies will be made by the project data manager on password protect file space. When the project is completed, the full database will be

For peer review only - http://bmjopen.bmj.com/site/about/guidelines.xhtml

stored on both DPHRU and University servers. The items and metadata on the institutional repository are backed up on a daily basis and these backups are kept on tape. All data collection devices will be encrypted.

Data from this study will be stored for a minimum of 10 years from the completion of the study in order to allow for any verification of data. Long-term data storage will be in .csv files using a standard (simple) ASCII coding scheme in line with UK Data Service recommendations:

(https://www.ukdataservice.ac.uk/manage-data/format/recommended-formats).

Acknowledgements

The authors would like to thank the Wellcome Trust and the NIHR for their financial support which made this study possible.

Contributors

AP is the principle investigator for the PLAY study. AP, SAN, RP, LR and DAL conceptualised the study design. AP wrote the initial draft of the protocol. SAN, RP, LR, DAL, DM, FB, SJC, and HT read and contributed to the final version. All authors provided edits and critiqued the manuscript for intellectual content.

Funding

This work was supported by the National Institute for Health Research (NIHR) (using the UK's Official Development Assistance (ODA) Funding) and Wellcome [222007/Z/20/Z] under the NIHR-Wellcome Partnership for Global Health Research. The views expressed are those of the authors and not necessarily those of Wellcome, the NIHR or the Department of Health and Social Care.

Competing interests

The authors have no conflicts of interest to report

Patient and public involvement

Participants and the public are involved in the design, or conduct, or reporting, or dissemination plans of this research. Refer to the Methods section for further details.

Patient consent for publication

Not required.

Ethical approval

For peer review only - http://bmjopen.bmj.com/site/about/guidelines.xhtml

Ethical approval has been granted for the establishment of the community advisory group (M210846), and for the trial (M220217) from the Human Ethics Research Committee of the University of the Witwatersrand, Johannesburg, South Africa. Additional approval has been granted by the Research Committee of Johannesburg Health District (GP_202202_021).

Provenance and peer review

Not commissioned; peer-reviewed for ethical and funding approval prior to submission.

For peer review only

References

1. Black MM, Walker SP, Fernald LCH, et al. Early childhood development coming of age: science through the life course. *The Lancet* 2017;389(10064):77-90. doi: 10.1016/s0140-6736(16)31389-7
2. Lu C, Black MM, Richter LM. Risk of poor development in young children in low-income and middle-income countries: an estimation and analysis at the global, regional, and country level. *The Lancet Global Health* 2016;4(12):e916-e22. doi: 10.1016/s2214-109x(16)30266-2
3. Collaborators LBDDBoM. Mapping local patterns of childhood overweight and wasting in low- and middle-income countries between 2000 and 2017. *Nat Med* 2020;26(5):750-59. doi: 10.1038/s41591-020-0807-6 [published Online First: 2020/04/22]
4. Aboud FE, Yousafzai AK. Global health and development in early childhood. *Annu Rev Psychol* 2015;66:433-57. doi: 10.1146/annurev-psych-010814-015128 [published Online First: 2014/09/10]
5. Cantor P, Osher D, Berg J, et al. Malleability, plasticity, and individuality: How children learn and develop in context1. *Applied Developmental Science* 2018;23(4):307-37. doi: 10.1080/10888691.2017.1398649
6. World Health Organization. Improving early childhood development: WHO guideline, 2020.
7. Victora CG, Bahl R, Barros AJD, et al. Breastfeeding in the 21st century: epidemiology, mechanisms, and lifelong effect. *The Lancet* 2016;387(10017):475-90. doi: 10.1016/s0140-6736(15)01024-7
8. World Health Organisation. World Health Organization (2003) Global strategy for infant and young child feeding. In: Organisation WH, ed. WHO Geneva, 2003.
9. Nieuwoudt S, Manderson L, Norris SA. Infant feeding practices in Soweto, South Africa: Implications for healthcare providers. *S Afr Med J* 2018;108(9):756-62. doi: 10.7196/SAMJ.2018.v108i9.13358 [published Online First: 2018/09/06]
10. Mushaphi LF, Mahopo TC, Nesamvuni CN, et al. Recommendations for Infant Feeding Policy and Programs in Dzimauli Region, South Africa: Results From the MAL-ED Birth Cohort. *Food Nutr Bull* 2017;38(3):428-40. doi: 10.1177/0379572117696662 [published Online First: 2017/09/30]
11. Goosen C, McLachlan M, Schübl C. Infant feeding practices during the first 6 months of life in a low-income area of the Western Cape Province. *South African Journal of Child Health* 2014;8(2) doi: 10.7196/sajch.675
12. Jama NA, Wilford A, Masango Z, et al. Enablers and barriers to success among mothers planning to exclusively breastfeed for six months: a qualitative prospective cohort study in KwaZulu-Natal, South Africa. *Int Breastfeed J* 2017;12:43. doi: 10.1186/s13006-017-0135-8 [published Online First: 2017/10/14]
13. Wrottesley SV, Prioreschi A, Slemming W, et al. Maternal perspectives on infant feeding practices in Soweto, South Africa. *Public Health Nutr* 2020:1-13. doi: 10.1017/S1368980020002451 [published Online First: 2020/08/11]
14. World Health Organization. Guidelines on physical activity, sedentary

For peer review only

behaviour and sleep for children under 5 years of age. *Geneva: Licence: CC BY-NC-SA 30 IGO* 2019
15. Draper CE, Tomaz SA, Biersteker L, et al. The South African 24-Hour Movement Guidelines for Birth to 5 Years: An Integration of Physical Activity, Sitting Behavior, Screen Time, and Sleep. *J Phys Act Health* 2020;17(1):109-19. doi: 10.1123/jpah.2019-0187 [published Online First: 2019/12/27]
16. Tremblay M, Chaput J, Adamo K, et al. Canadian 24-Hour Movement Guidelines for the Early Years (0–4 years): An Integration of Physical Activity, Sedentary Behaviour, and Sleep. *BMC Public Health* 2017;17 (Suppl 5):874. doi: 10.1186/s12889-017-4859-6
17. Ginsburg KR, American Academy of Pediatrics Committee on C, American Academy of Pediatrics Committee on Psychosocial Aspects of C, et al. The importance of play in promoting healthy child development and maintaining strong parent-child bonds. *Pediatrics* 2007;119(1):182-91. doi: 10.1542/peds.2006-2697 [published Online First: 2007/01/04]
18. Markova G. The Games Infants Play: Social Games During Early Mother-Infant Interactions and Their Relationship With Oxytocin. *Front Psychol* 2018;9:1041. doi: 10.3389/fpsyg.2018.01041 [published Online First: 2018/07/11]
19. Tremblay MS, Gray C, Babcock S, et al. Position Statement on Active Outdoor Play. *Int J Environ Res Public Health* 2015;12(6):6475-505. doi: 10.3390/ijerph120606475 [published Online First: 2015/06/11]
20. World Health Organization, United Nations Children's Fund, Group. WB. Nurturing care for early childhood development: a framework for helping children survive and thrive to transform health and human potential. Geneva: World Health Organization, 2018.
21. Prioreschi A, Brage S, Hesketh KD, et al. Describing objectively measured physical activity levels, patterns, and correlates in a cross sectional sample of infants and toddlers from South Africa. *Int J Behav Nutr Phys Act* 2017;14(1):176. doi: 10.1186/s12966-017-0633-5 [published Online First: 2017/12/24]
22. Prioreschi A, KK O, E DLR, et al. Associations between abdominal adiposity, body size and objectively measured physical activity in infants from Soweto, South Africa. *Maternal and Child Health* 2022;In press doi: DOI: 10.1007/s10995-022-03406-5
23. Prioreschi A, Micklesfield LK. Compliance With Physical Activity and Sedentary Behavior Guidelines and Associations With Abdominal Adiposity in a Sample of Infants and Toddlers From Soweto, South Africa. *J Phys Act Health* 2019;16(12):872-79. doi: 10.1123/jpah.2018-0182 [published Online First: 2019/07/19]
24. Prioreschi A, Brage S, Westgate K, et al. Describing the diurnal relationships between objectively measured mother and infant physical activity. *Int J Behav Nutr Phys Act* 2018;15(1):59. doi: 10.1186/s12966-018-0692-2 [published Online First: 2018/06/27]
25. Jeong J, Pitchik HO, Yousafzai AK. Stimulation Interventions and Parenting in Low- and Middle-Income Countries: A Meta-analysis. *Pediatrics* 2018;141(4) doi: 10.1542/peds.2017-3510 [published Online First: 2018/03/04]
26. Prioreschi A, Wrottesley SV, Slemming W, et al. A qualitative study reporting maternal perceptions of the importance of play for healthy growth and development in the first two years of life. *BMC Pediatr* 2020;20(1):428. doi: 10.1186/s12887-020-02321-4 [published Online First: 2020/09/11]

For peer review only - http://bmjopen.bmj.com/site/about/guidelines.xhtml

27. Hall K, Richter L, Mokomane Z, et al. South African Child Gauge 2018. *Cape Town: Children's Institute, University of Cape Town ISBN: 978-0-620-81934-3* 2018
28. Mokwena K, Masike I. The Need for Universal Screening for Postnatal Depression in South Africa: Confirmation from a Sub-District in Pretoria, South Africa. *Int J Environ Res Public Health* 2020;17(19) doi: 10.3390/ijerph17196980 [published Online First: 2020/09/30]
29. Cohen E, Ware L, Prioreschi A, et al. Material and relational difficulties: The Impact of the Household Environment on the Emotional Well-Being of Young Black Women Living in Soweto, South Africa. *Journal of Family Issues* 2019;41(8):1307-32.
30. Ware LJ, Kim AW, Prioreschi A, et al. Social vulnerability, parity and food insecurity in urban South African young women: the healthy life trajectories initiative (HeLTI) study. *J Public Health Policy* 2021;42(3):373-89. doi: 10.1057/s41271-021-00289-8 [published Online First: 2021/05/21]
31. Verkuijl NE, Richter L, Norris SA, et al. Postnatal depressive symptoms and child psychological development at 10 years: a prospective study of longitudinal data from the South African Birth to Twenty cohort. *The Lancet Psychiatry* 2014;1(6):454-60. doi: 10.1016/s2215-0366(14)70361-x
32. Koen N, Brittain K, Donald KA, et al. Maternal posttraumatic stress disorder and infant developmental outcomes in a South African birth cohort study. *Psychol Trauma* 2017;9(3):292-300. doi: 10.1037/tra0000234 [published Online First: 2017/05/02]
33. Bosquet Enlow M, Kitts RL, Blood E, et al. Maternal posttraumatic stress symptoms and infant emotional reactivity and emotion regulation. *Infant Behav Dev* 2011;34(4):487-503. doi: 10.1016/j.infbeh.2011.07.007 [published Online First: 2011/08/25]
34. Hill R, Flanagan J. The maternal-infant Bond: Clarifying the concept. *International Journal of Nursing Knowledge* 2020;31(1):14-18.
35. Jeong J, Franchett EE, Ramos de Oliveira CV, et al. Parenting interventions to promote early child development in the first three years of life: A global systematic review and meta-analysis. *PLoS Med* 2021;18(5):e1003602. doi: 10.1371/journal.pmed.1003602 [published Online First: 2021/05/11]
36. Richter L. The importance of caregiver-child interactions for the survival and healthy development of young children: a review. *World Health Organisation* 2004
37. Bernard K, Meade EB, Dozier M. Parental synchrony and nurturance as targets in an attachment based intervention: building upon Mary Ainsworth's insights about mother-infant interaction. *Attach Hum Dev* 2013;15(5-6):507-23. doi: 10.1080/14616734.2013.820920 [published Online First: 2013/12/05]
38. Brazeau N, Reisz S, Jacobvitz D, et al. Understanding the Connection between Attachment Trauma and Maternal Self-Efficacy in Depressed Mothers. *Infant Ment Health J* 2018;39(1):30-43. doi: 10.1002/imhj.21692 [published Online First: 2017/12/28]
39. Leerkes E, Crockenberg S. The Development of Maternal Self-Efficacy and Its Impact on Maternal Behavior. *Infancy* 2002;3(2):227-47.
40. Dozier M, Bernard K. Attachment and Biobehavioral Catch-up: Addressing the Needs of Infants and Toddlers Exposed to Inadequate or Problematic Caregiving. *Curr Opin Psychol* 2017;15:111-17. doi: 10.1016/j.copsyc.2017.03.003 [published Online First: 2017/06/27]

For peer review only

41. Kaminski JW, Valle LA, Filene JH, et al. A meta-analytic review of components associated with parent training program effectiveness. *J Abnorm Child Psychol* 2008;36(4):567-89. doi: 10.1007/s10802-007-9201-9 [published Online First: 2008/01/22]
42. Michie S, van Stralen MM, West R. The behaviour change wheel: a new method for characterising and designing behaviour change interventions. *Implement Sci* 2011;6:42. doi: 10.1186/1748-5908-6-42 [published Online First: 2011/04/26]
43. Caron EB, Bernard K, Dozier M. In Vivo Feedback Predicts Parent Behavior Change in the Attachment and Biobehavioral Catch-up Intervention. *J Clin Child Adolesc Psychol* 2018;47(sup1):S35-S46. doi: 10.1080/15374416.2016.1141359 [published Online First: 2016/04/05]
44. Carey RN, Connell LE, Johnston M, et al. Behavior Change Techniques and Their Mechanisms of Action: A Synthesis of Links Described in Published Intervention Literature. *Ann Behav Med* 2019;53(8):693-707. doi: 10.1093/abm/kay078 [published Online First: 2018/10/12]
45. Larson EL, Patel SJ, Evans D, et al. Feedback as a strategy to change behaviour: the devil is in the details. *J Eval Clin Pract* 2013;19(2):230-4. doi: 10.1111/j.1365-2753.2011.01801.x [published Online First: 2011/12/02]
46. Michie S, Wood CE, Johnston M, et al. Behaviour change techniques: the development and evaluation of a taxonomic method for reporting and describing behaviour change interventions (a suite of five studies involving consensus methods, randomised controlled trials and analysis of qualitative data). *Health Technol Assess* 2015;19(99):1-188. doi: 10.3310/hta19990 [published Online First: 2015/12/01]
47. Van Zeijl J, Mesman J, Van IMH, et al. Attachment-based intervention for enhancing sensitive discipline in mothers of 1- to 3-year-old children at risk for externalizing behavior problems: a randomized controlled trial. *J Consult Clin Psychol* 2006;74(6):994-1005. doi: 10.1037/0022-006X.74.6.994 [published Online First: 2006/12/13]
48. Richards K, Enderlin C, Beck C, et al. Tailored Biobehavioral Interventions: A Literature Review and Synthesis. *Research and Theory for Nursing Practice* 2007;21(4):271-85.
49. Chan AW, Tetzlaff JM, Gotzsche PC, et al. SPIRIT 2013 explanation and elaboration: guidance for protocols of clinical trials. *BMJ* 2013;346:e7586. doi: 10.1136/bmj.e7586 [published Online First: 2013/01/11]
50. Nugent JK, Bartlett JD, Von Ende A, et al. The Effects of the Newborn Behavioral Observations (NBO) System on Sensitivity in Mother–Infant Interactions. *Infants & Young Children* 2017;30(4):257-68. doi: 10.1097/iyc.0000000000000103
51. van IMH, Schuengel C, Wang Q, et al. Improving parenting, child attachment, and externalizing behaviors: Meta-analysis of the first 25 randomized controlled trials on the effects of Video-feedback Intervention to promote Positive Parenting and Sensitive Discipline. *Dev Psychopathol* 2022:1-16. doi: 10.1017/S0954579421001462 [published Online First: 2022/01/18]
52. Sekhon, M., M. Cartwright, and J.J. Francis. Acceptability of healthcare interventions: an overview of reviews and development of a theoretical framework. B*MC Health Serv Res* 2017;17(1): p. 88.
53. MRC. Process evaluation of complex interventions: Medical Research Council guidance. *BMJ* 2015;350.

For peer review only - http://bmjopen.bmj.com/site/about/guidelines.xhtml

54. Dennis, C.L. The breastfeeding self-efficacy scale: psychometric assessment of the short form. *J Obstet Gynecol Neonatal Nurs* 2003;32(6): p. 734-44.
55. Osborne, R., et al., The grounded psychometric development and initial validation of the Health Literacy Questionnaire (HLQ). *BMC Public Health* 2013;13(658).
56. Blevins, C.A., et al., The Posttraumatic Stress Disorder Checklist for DSM-5 (PCL-5): Development and Initial Psychometric Evaluation. *J Trauma Stress* 2015;28(6): p. 489-98.
57. Cox, J.L., J.M. Holden, and R. Sagovsky. Detection of postnatal depression. Development of the 10-item Edinburgh Postnatal Depression Scale. *Br J Psychiatry* 1987;150: p. 782-6.
58, Felitti, V.J., et al., Relationship of Childhood Abuse and Household Dysfunction to Many of the Leading Causes of Death in Adults. The Adverse Childhood Experiences (ACE) Study. *American Journal of Preventive Medicine* 1998; 14(4): p. 245-258.
59. Brockington, I.F., C. Fraser, and D. Wilson. The Postpartum Bonding Questionnaire: a validation. *Arch Womens Ment Health* 2006; 9(5): p. 233-42.
60. Ballot DE, Ramdin T, Rakotsoane D, et al. Use of the Bayley Scales of Infant and Toddler Development, Third Edition, to Assess Developmental Outcome in Infants and Young Children in an Urban Setting in South Africa. *Int Sch Res Notices* 2017;2017:1631760. doi: 10.1155/2017/1631760 [published Online First: 2017/08/25]

For peer review only

Figures

Figure 1. Participant flow chart

Figure 2. Intervention themes and assessments

Figure 3. PLAY Study logic model

For peer review only

For peer review only - http://bmjopen.bmj.com/site/about/guidelines.xhtml

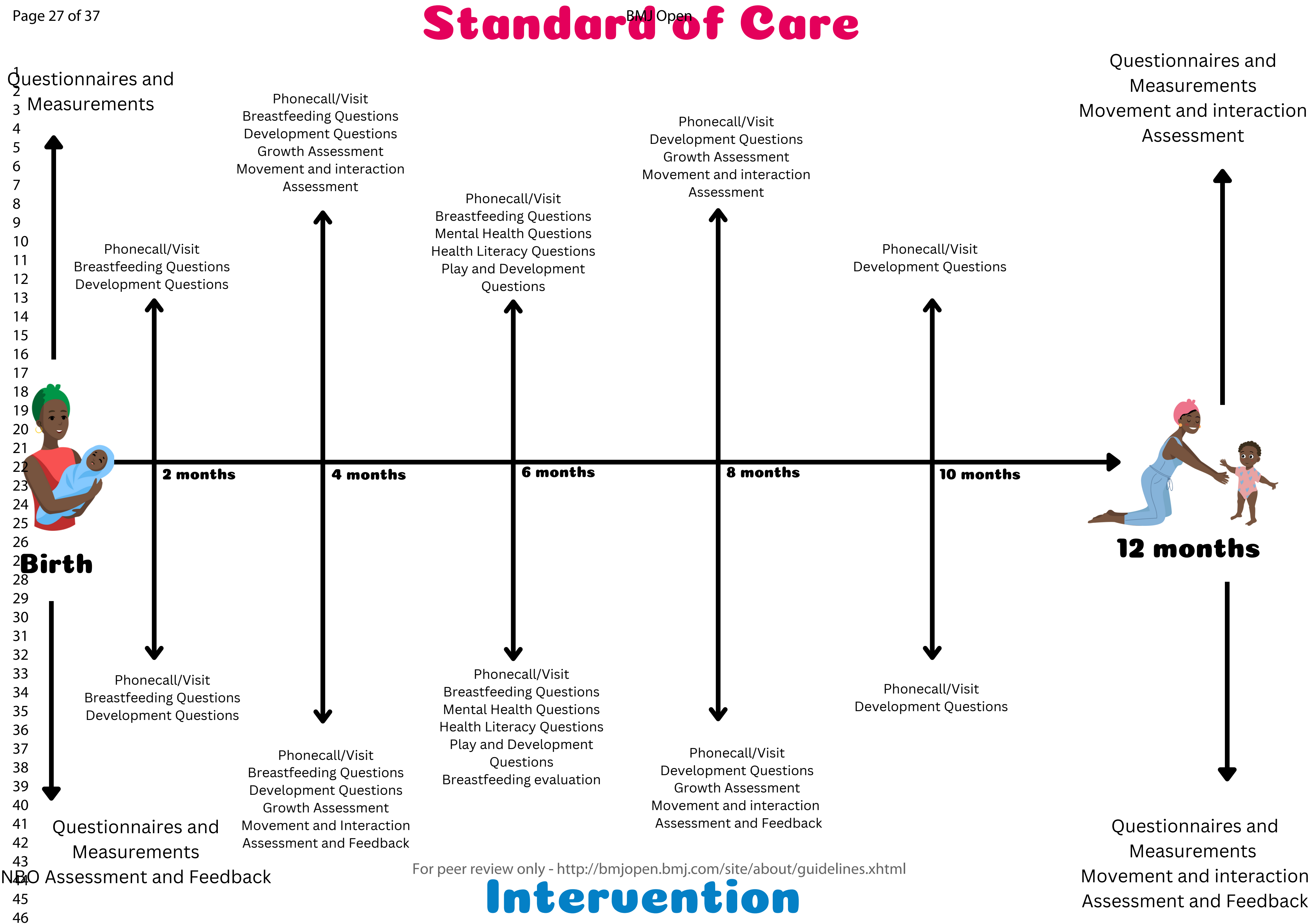
Standard of Care
Questionnaires and Measurements
Birth
Phonecall/Visit
Breastfeeding Questions
Development Questions
2 months
Phonecall/Visit
Breastfeeding Questions
Development Questions
Growth Assessment
Movement and interaction Assessment
4 months
Phonecall/Visit
Breastfeeding Questions
Mental Health Questions
Health Literacy Questions
Play and Development Questions
6 months
Phonecall/Visit
Development Questions
Growth Assessment
Movement and interaction Assessment
8 months
Phonecall/Visit
Development Questions
10 months
Questionnaires and Measurements
Movement and interaction Assessment
12 months
Questionnaires and Measurements
NBO Assessment and Feedback
Phonecall/Visit
Breastfeeding Questions
Development Questions
Phonecall/Visit
Breastfeeding Questions
Development Questions
Growth Assessment
Movement and Interaction Assessment and Feedback
Phonecall/Visit
Breastfeeding Questions
Mental Health Questions
Health Literacy Questions
Play and Development Questions
Breastfeeding evaluation
Phonecall/Visit
Development Questions
Growth Assessment
Movement and interaction Assessment and Feedback
Phonecall/Visit
Development Questions
Questionnaires and Measurements
Movement and interaction Assessment and Feedback
Interuention

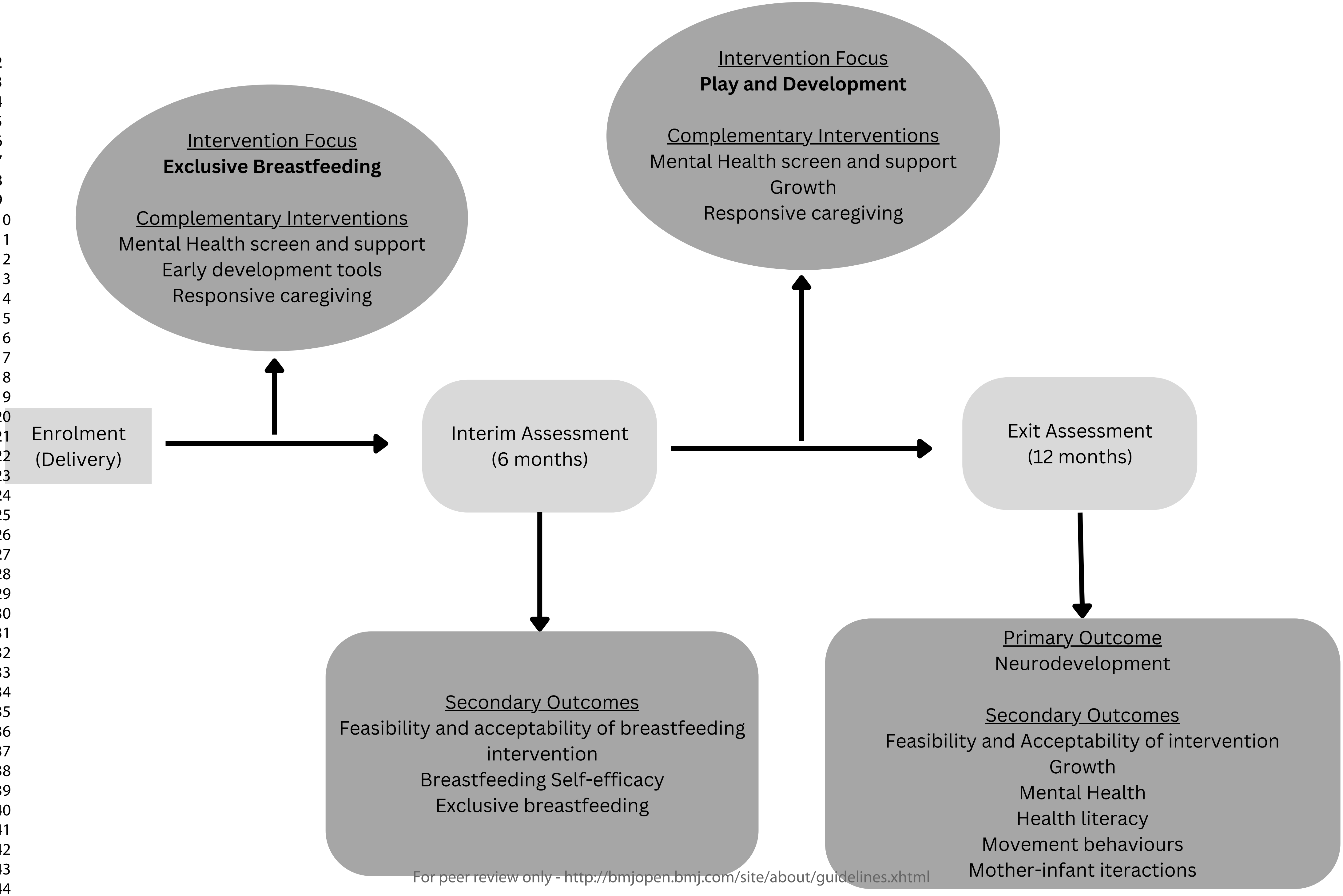

For peer review only - http://bmjopen.bmj.com/site/about/guidelines.xhtml

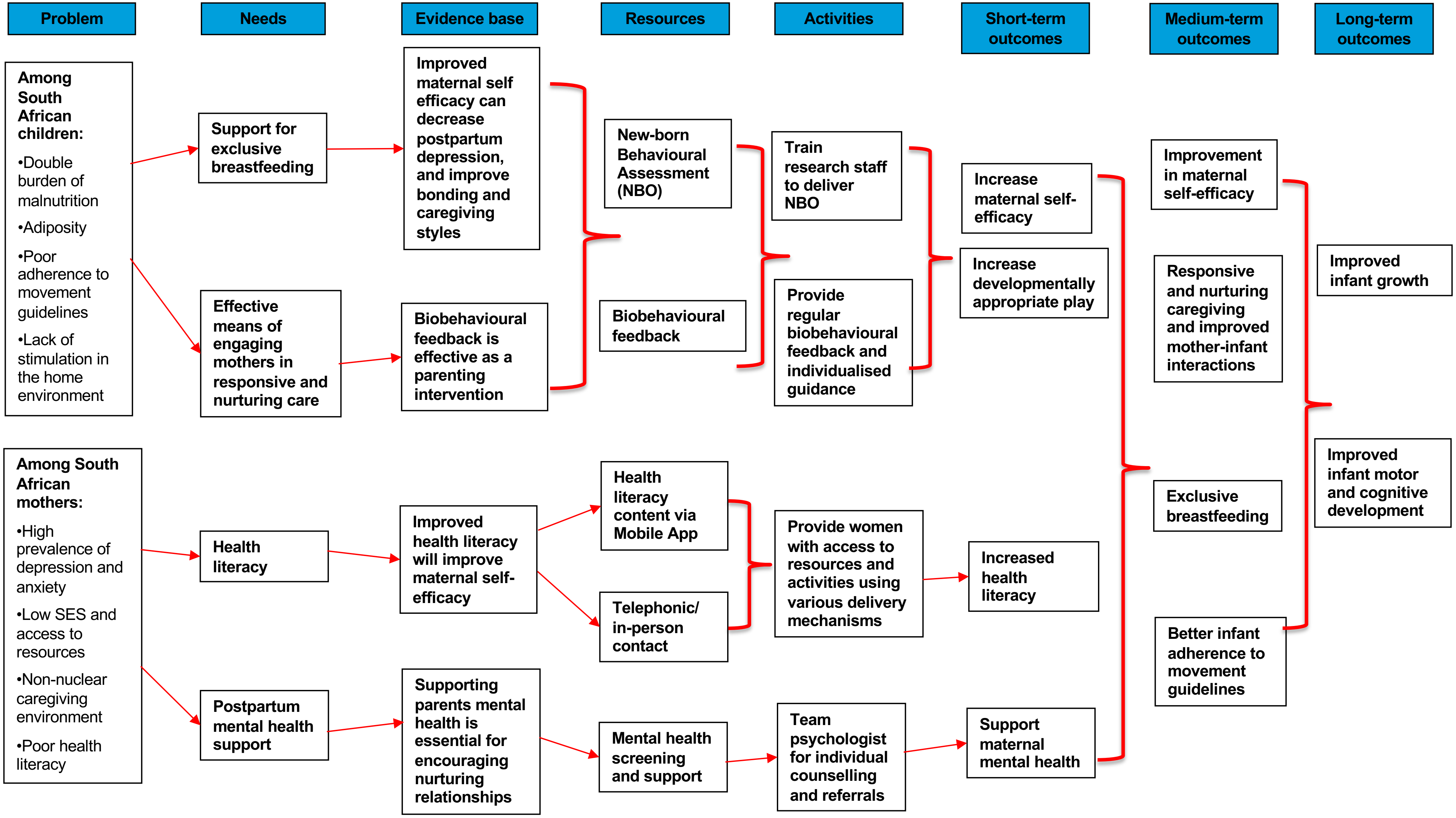
Problem
Needs
Evidence base
Resources
Activities
Short-term outcomes
Medium-term outcomes
Long-term outcomes
Among South African children:
•Double burden of malnutrition
•Adiposity
•Poor adherence to movement guidelines
•Lack of stimulation in the home environment
Support for exclusive breastfeeding
Effective means of engaging mothers in responsive and nurturing care
Improved maternal self efficacy can decrease postpartum depression, and improve bonding and caregiving styles
Biobehavioural feedback is effective as a parenting intervention
New-born Behavioural Assessment (NBO)
Biobehavioural feedback
Train research staff to deliver NBO
Provide regular biobehavioural feedback and individualised guidance
Increase maternal self-efficacy
Increase developmentally appropriate play
Improvement in maternal self-efficacy
Responsive and nurturing caregiving and improved mother-infant interactions
Improved infant growth
Among South African mothers:
•High prevalence of depression and anxiety
•Low SES and access to resources
•Non-nuclear caregiving environment
•Poor health literacy
Health literacy
Postpartum mental health support
Improved health literacy will improve maternal self-efficacy
Supporting parents mental health is essential for encouraging nurturing relationships
Health literacy content via Mobile App
Telephonic/ in-person contact
Mental health screening and support
Provide women with access to resources and activities using various delivery mechanisms
Team psychologist for individual counselling and referrals
Increased health literacy
Support maternal mental health
Exclusive breastfeeding
Better infant adherence to movement guidelines
Improved infant motor and cognitive development

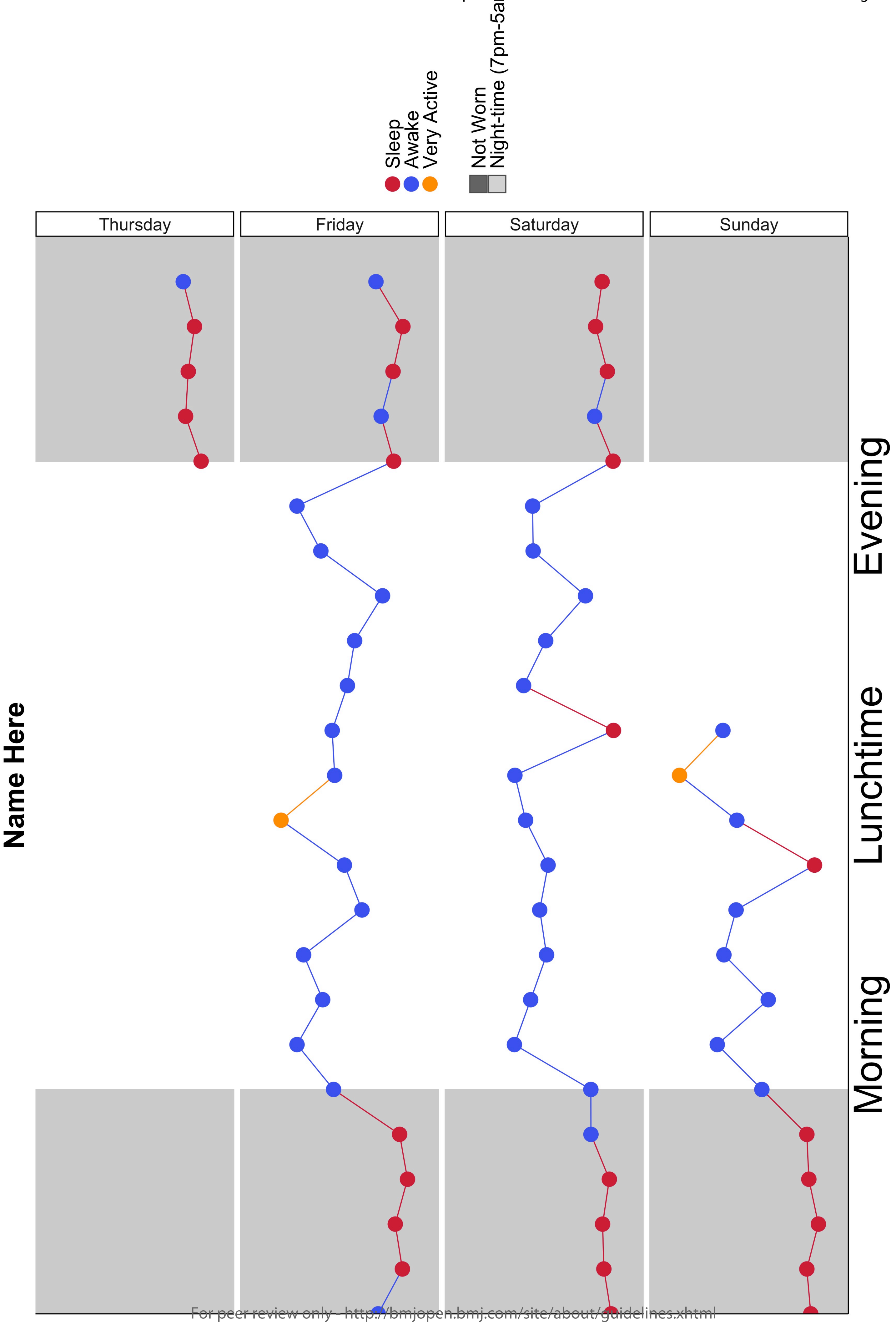

For peer review only - http://bmjopen.bmj.com/site/about/guidelines.xhtml

Supplementary Figure 2. Sleep, screen time, routines and daily activities

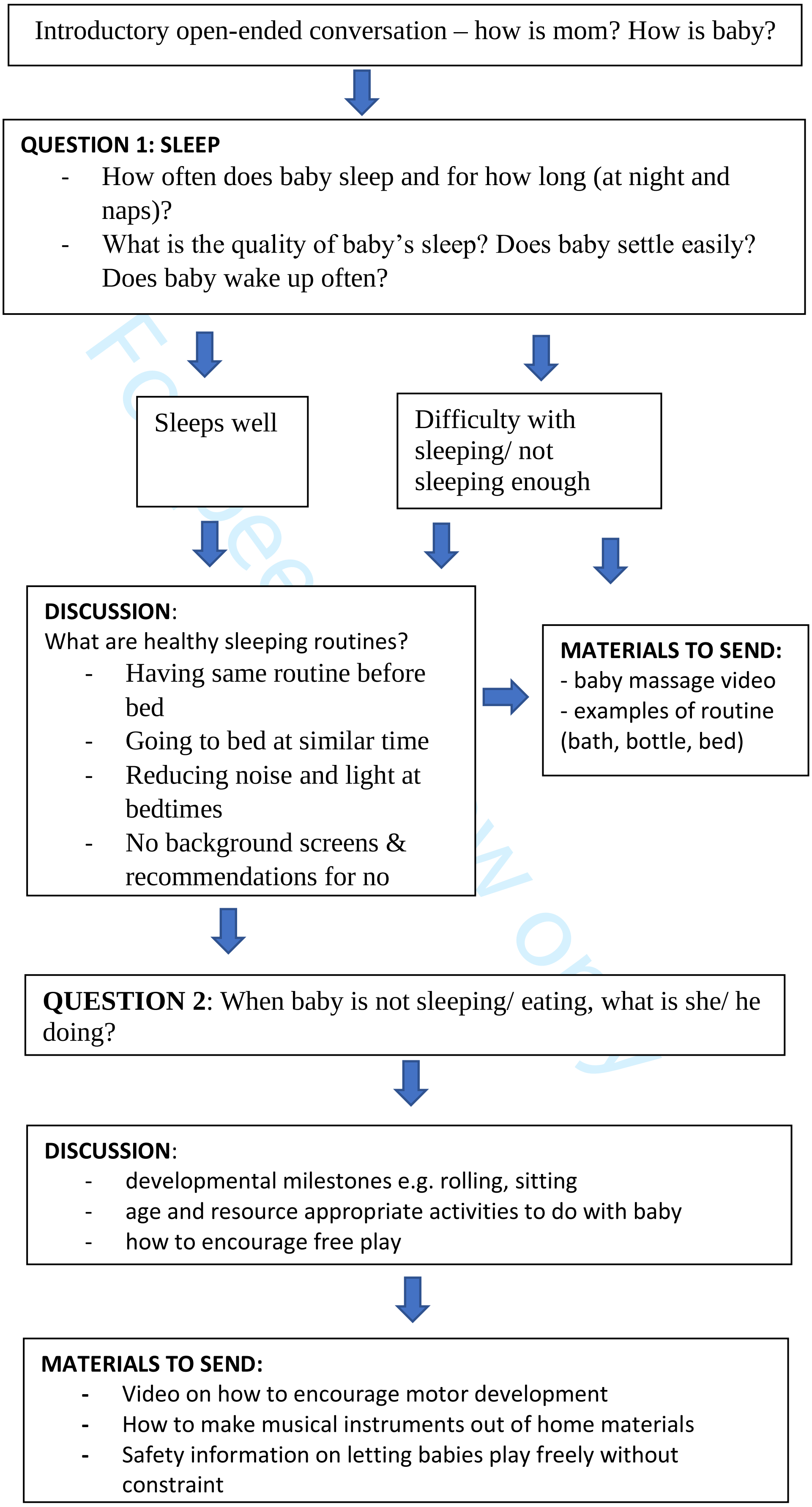

Professor and Head of School of Computer Science,

Electrical and [illegible]

University of [illegible]

SPIRIT

STANDARD PROTOCOL ITEMS: RECOMMENDATIONS FOR INTERVENTIONAL TRIALS

SPIRIT 2013 Checklist: Recommended items to address in a clinical trial protocol and related documents*

Section/item	Item No	Description	Page number
**Administrative information**			
Title	1	Descriptive title identifying the study design, population, interventions, and, if applicable, trial acronym	Title page
Trial registration	2a	Trial identifier and registry name. If not yet registered, name of intended registry	2
	2b	All items from the World Health Organization Trial Registration Data Set	
Protocol version	3	Date and version identifier	2
Funding	4	Sources and types of financial, material, and other support	14
Roles and responsibilities	5a	Names, affiliations, and roles of protocol contributors	1, 14
	5b	Name and contact information for the trial sponsor	
	5c	Role of study sponsor and funders, if any, in study design; collection, management, analysis, and interpretation of data; writing of the report; and the decision to submit the report for publication, including whether they will have ultimate authority over any of these activities	14

For peer review only

	5d	Composition, roles, and responsibilities of the coordinating centre, steering committee, endpoint adjudication committee, data management team, and other individuals or groups overseeing the trial, if applicable (see Item 21a for data monitoring committee)	
**Introduction**			
Background and rationale	6a	Description of research question and justification for undertaking the trial, including summary of relevant studies (published and unpublished) examining benefits and harms for each intervention	4-6
	6b	Explanation for choice of comparators	6-7
Objectives	7	Specific objectives or hypotheses	6-7
Trial design	8	Description of trial design including type of trial (eg, parallel group, crossover, factorial, single group), allocation ratio, and framework (eg, superiority, equivalence, noninferiority, exploratory)	7
**Methods: Participants, interventions, and outcomes**			
Study setting	9	Description of study settings (eg, community clinic, academic hospital) and list of countries where data will be collected. Reference to where list of study sites can be obtained	7
Eligibility criteria	10	Inclusion and exclusion criteria for participants. If applicable, eligibility criteria for study centres and individuals who will perform the interventions (eg, surgeons, psychotherapists)	7
Interventions	11a	Interventions for each group with sufficient detail to allow replication, including how and when they will be administered	7-10; Figure 1 and 2
	11b	Criteria for discontinuing or modifying allocated interventions for a given trial participant (eg, drug dose change in response to harms, participant request, or improving/worsening disease)	NA
	11c	Strategies to improve adherence to intervention protocols, and any procedures for monitoring adherence (eg, drug tablet return, laboratory tests)	11-12

For peer review

	11d	Relevant concomitant care and interventions that are permitted or prohibited during the trial	7-10
Outcomes	12	Primary, secondary, and other outcomes, including the specific measurement variable (eg, systolic blood pressure), analysis metric (eg, change from baseline, final value, time to event), method of aggregation (eg, median, proportion), and time point for each outcome. Explanation of the clinical relevance of chosen efficacy and harm outcomes is strongly recommended	10-12; Figure 2
Participant timeline	13	Time schedule of enrolment, interventions (including any run-ins and washouts), assessments, and visits for participants. A schematic diagram is highly recommended (see Figure)	Table 1; Figure 2
Sample size	14	Estimated number of participants needed to achieve study objectives and how it was determined, including clinical and statistical assumptions supporting any sample size calculations	13
Recruitment	15	Strategies for achieving adequate participant enrolment to reach target sample size	7; 11; 13
**Methods: Assignment of interventions (for controlled trials)**			
Allocation:			
Sequence generation	16a	Method of generating the allocation sequence (eg, computer-generated random numbers), and list of any factors for stratification. To reduce predictability of a random sequence, details of any planned restriction (eg, blocking) should be provided in a separate document that is unavailable to those who enrol participants or assign interventions	7
Allocation concealment mechanism	16b	Mechanism of implementing the allocation sequence (eg, central telephone; sequentially numbered, opaque, sealed envelopes), describing any steps to conceal the sequence until interventions are assigned	7
Implementation	16c	Who will generate the allocation sequence, who will enrol participants, and who will assign participants to interventions	7

Blinding (masking)	17a	Who will be blinded after assignment to interventions (eg, trial participants, care providers, outcome assessors, data analysts), and how	7
	17b	If blinded, circumstances under which unblinding is permissible, and procedure for revealing a participant's allocated intervention during the trial	NA
**Methods: Data collection, management, and analysis**			
Data collection methods	18a	Plans for assessment and collection of outcome, baseline, and other trial data, including any related processes to promote data quality (eg, duplicate measurements, training of assessors) and a description of study instruments (eg, questionnaires, laboratory tests) along with their reliability and validity, if known. Reference to where data collection forms can be found, if not in the protocol	10-13
	18b	Plans to promote participant retention and complete follow-up, including list of any outcome data to be collected for participants who discontinue or deviate from intervention protocols	7; 11; 13
Data management	19	Plans for data entry, coding, security, and storage, including any related processes to promote data quality (eg, double data entry; range checks for data values). Reference to where details of data management procedures can be found, if not in the protocol	13-14
Statistical methods	20a	Statistical methods for analysing primary and secondary outcomes. Reference to where other details of the statistical analysis plan can be found, if not in the protocol	13; Figure 2
	20b	Methods for any additional analyses (eg, subgroup and adjusted analyses)	Figure 2
	20c	Definition of analysis population relating to protocol non-adherence (eg, as randomised analysis), and any statistical methods to handle missing data (eg, multiple imputation)	13
**Methods: Monitoring**			

Data monitoring	21a	Composition of data monitoring committee (DMC); summary of its role and reporting structure; statement of whether it is independent from the sponsor and competing interests; and reference to where further details about its charter can be found, if not in the protocol. Alternatively, an explanation of why a DMC is not needed	
	21b	Description of any interim analyses and stopping guidelines, including who will have access to these interim results and make the final decision to terminate the trial	Figure 2
Harms	22	Plans for collecting, assessing, reporting, and managing solicited and spontaneously reported adverse events and other unintended effects of trial interventions or trial conduct	11-12
Auditing	23	Frequency and procedures for auditing trial conduct, if any, and whether the process will be independent from investigators and the sponsor	
**Ethics and dissemination**			
Research ethics approval	24	Plans for seeking research ethics committee/institutional review board (REC/IRB) approval	20-21
Protocol amendments	25	Plans for communicating important protocol modifications (eg, changes to eligibility criteria, outcomes, analyses) to relevant parties (eg, investigators, REC/IRBs, trial participants, trial registries, journals, regulators)	2; 15
Consent or assent	26a	Who will obtain informed consent or assent from potential trial participants or authorised surrogates, and how (see Item 32)	14-15
	26b	Additional consent provisions for collection and use of participant data and biological specimens in ancillary studies, if applicable	
Confidentiality	27	How personal information about potential and enrolled participants will be collected, shared, and maintained in order to protect confidentiality before, during, and after the trial	7; 13-14

For peer review only - http://bmjopen.bmj.com/site/about/guidelines.xhtml

Declaration of interests	28	Financial and other competing interests for principal investigators for the overall trial and each study site	14-15
Access to data	29	Statement of who will have access to the final trial dataset, and disclosure of contractual agreements that limit such access for investigators	14
Ancillary and post-trial care	30	Provisions, if any, for ancillary and post-trial care, and for compensation to those who suffer harm from trial participation	
Dissemination policy	31a	Plans for investigators and sponsor to communicate trial results to participants, healthcare professionals, the public, and other relevant groups (eg, via publication, reporting in results databases, or other data sharing arrangements), including any publication restrictions	
	31b	Authorship eligibility guidelines and any intended use of professional writers	14
	31c	Plans, if any, for granting public access to the full protocol, participant-level dataset, and statistical code	
**Appendices**			
Informed consent materials	32	Model consent form and other related documentation given to participants and authorised surrogates	Supplementary
Biological specimens	33	Plans for collection, laboratory evaluation, and storage of biological specimens for genetic or molecular analysis in the current trial and for future use in ancillary studies, if applicable	NA

*It is strongly recommended that this checklist be read in conjunction with the SPIRIT 2013 Explanation & Elaboration for important clarification on the items. Amendments to the protocol should be tracked and dated. The SPIRIT checklist is copyrighted by the SPIRIT Group under the Creative Commons "Attribution-NonCommercial-NoDerivs 3.0 Unported" license.

For peer review only - http://bmjopen.bmj.com/site/about/guidelines.xhtml

BMJ Open

Protocol for the PLAY study: a randomised controlled trial of an intervention to improve infant development by encouraging maternal self-efficacy using behavioural feedback

Journal:	*BMJ Open*
Manuscript ID	bmjopen-2022-064976.R2
Article Type:	Protocol
Date Submitted by the Author:	22-Feb-2023
Complete List of Authors:	Prioreschi, Alessandra; University of the Witwatersrand, SAMRC/Wits Developmental Pathways for Health Research Unit<br>Pearson, Rebecca; University of Bristol, Centre for Academic Mental Health, Addiction and Suicide Research, School of Social & Community Medicine; University of the Witwatersrand, SAMRC/Wits Developmental Pathways for Health Research Unit<br>Richter, Linda; University of the Witwatersrand, DSI-NRF Centre of Excellence in Child Development<br>Bennin, Fiona; University of the Witwatersrand, SAMRC/Wits Developmental Pathways for Health Research Unit<br>Theunissen, Helene; University of the Witwatersrand, SAMRC/Wits Developmental Pathways for Health Research Unit<br>Cantrell, Sarah J; University of the Witwatersrand, SAMRC/Wits Developmental Pathways for Health Research Unit<br>Maduna, Dumsile; University of the Witwatersrand, SAMRC/Wits Developmental Pathways for Health Research Unit<br>Lawlor, Deborah; Department of Social Medicine, University of Bristol, MRC Integrative Epidemiology Unit<br>Norris, Shane; University of the Witwatersrand Faculty of Health Sciences, SAMRC/Wits Developmental Pathways for Health Research Unit
**Primary Subject Heading**:	Public health
Secondary Subject Heading:	Global health, Mental health, Nutrition and metabolism, Paediatrics
Keywords:	Community child health < PAEDIATRICS, PUBLIC HEALTH, EPIDEMIOLOGY

SCHOLARONE™
Manuscripts

Protocol for the PLAY study: a randomised controlled trial of an intervention to improve infant development by encouraging maternal self-efficacy using behavioural feedback

Alessandra Prioreschi[1*], Rebecca Pearson[1,2], Linda Richter[3], Fiona Bennin[1], Helene Theunissen[1], Sarah J Cantrell[1], Dumsile Maduna[1], Deborah A Lawlor[4], Shane A Norris[1,5]

1. SAMRC/Wits Developmental Pathways for Health Research Unit, Department of Paediatrics and Child Health, Faculty of Health Sciences, University of the Witwatersrand, Johannesburg, South Africa
2. Centre for Academic Mental Health, School of Social and Community Medicine, University of Bristol, Bristol, UK.
3. DSI-NRF Centre of Excellence in Child Development, University of the Witwatersrand
4. MRC Integrative Epidemiology Unit at the University of Bristol, UK
5. School of Human Development and Health and NIHR Southampton Biomedical Research Centre, Global Health Research Institute, University of Southampton, Southampton, UK

* Correspondence to:
Alessandra Prioreschi
Alessandra.prioreschi@wits.ac.za
SAMRC/Wits Developmental Pathways for Health Research Unit, Department of Paediatrics and Child Health, Faculty of Health Sciences, University of the Witwatersrand, Johannesburg, South Africa

For peer review only - http://bmjopen.bmj.com/site/about/guidelines.xhtml

Abstract

Introduction: The early infant caregiving environment is crucial in the formation of parent-child relationships, neurobehaviourial development, and thus child outcomes. This protocol describes the PLAY study, a phase 1 trial of an intervention designed to promote infant development through encouraging maternal self-efficacy using behavioral feedback, and supportive interventions.

Methods and analysis: 210 mother-infant pairs will be recruited at delivery from community clinics in Soweto, South Africa, and individually randomised (1:1) into two groups. The trial will consist of a standard of care arm and an intervention arm. The intervention will start at birth end at 12 months, and outcome assessments will be made when the infants are 0, 6, and 12 months of age. The intervention will be delivered by community health helpers (HHs) using an app with resource material, telephone calls, in person visits, and behavioural feedback with individualized support. Every 4 months mothers in the intervention group will receive rapid feedback via the app and in person on their infant's movement behaviours and on their interaction styles with their infant. At recruitment, and again at 4 months, mothers will be screened for mental health risk and women who score in the high-risk category will receive an individual counselling session from a licensed psychologist, followed by referral and continued support as necessary. The primary outcome is efficacy of the intervention in improving maternal self-efficacy, and the secondary outcomes are infant development at 12 months, and feasibility and acceptability of each component of the intervention.

Ethics and dissemination: The PLAY study has ethical approval from the Human Research Ethics Committee of the University of the Witwatersrand (M220217). Participants will be provided with an information sheet and required to provide written consent prior to being enrolled. Study results will be shared via publication in peer-reviewed journals, conference presentation and media engagement.

Trial registration: Registered with the Pan African Clinical Trials Registry (https://pactr.samrc.ac.za) on 10/02/2022, identifier PACTR202202747620052..

Protocol version: 16/02/2022 (version #2.0).

Keywords: behavioural feedback, play, early child development, responsive caregiving

Strengths and limitations of this study

- The PLAY study intervention uses behavioural feedback linked with personalised guidance to improve and individualise intervention content and implementation.

For peer review only - http://bmjopen.bmj.com/site/about/guidelines.xhtml

- The study will be guided by an advisory group of community members, consisting of women with infants and their mothers/influential family matriarchs, and consultation with this advisory group will happen before and during the intervention to ensure we remain contextually aware in creating and delivering our study content.
- The study uses advanced and robust measurement tools, which in some cases are novel in South Africa and/or globally, and thus should be able to generate reliable evidence that can inform future research.
- The study relies to some extent on the availability of cell phone or computer access for the delivery of intervention content, which may be limiting in the Soweto setting.
- The COVID pandemic may interfere with our recruitment and intervention strategies, and we have made the study less reliant on face-to-face interactions to accommodate any further lockdown restrictions in South Africa.

For peer review only

For peer review only - http://bmjopen.bmj.com/site/about/guidelines.xhtml

Introduction

Over the last decade, emphasis globally is being placed on improving early childhood development (ECD) [1]. Estimates are that 250 million children (43%) under the age of five living in low-and-middle-income countries (LMICs) are at risk of not meeting their developmental potential [2]. Furthermore, a double burden of malnutrition persists in LMICs, with wasting present in 6.4% and overweight present in 6% of children under age five [3]. While optimising growth and development requires a life course approach, the first two years are key in optimising developmental potential [1 4]. This highly sensitive period (the first 1000 days of life) is an experience-dependent and –expectant process which is plastic and malleable [5]. The World Health Organisation (WHO) has recently published four key guidelines for improving ECD [6]: integration of nutrition and caregiving interventions, promotion of early learning, supporting maternal mental health, and responsive caregiving.

Integrating nutrition into caregiving interventions

In the first 6 months of an infant's life, exclusive breastfeeding is regarded as the optimal and most nutritionally complete form of infant nutrition [7 8]. Research indicates that infants who are breastfed for longer may have higher cognitive function, and lower infectious morbidity and mortality. Breastfeeding may also protect against diabetes, cardiometabolic disease and overweight later in life [7]. It is estimated that improving global breastfeeding rates alone could potentially, prevent more than 800,000-child deaths annually [7]. Despite the widely established benefits of breastfeeding, in the South African context, roughly 67% of infants initiated breastfeeding within the first hour of birth in 2016, yet only 32% of children under the age of 6 months remain exclusively breastfed. The mean duration of exclusive breastfeeding was found to be only 2.9 months [9], with widespread introduction of solid foods and non-breastmilk liquids before 3 months [10 11]. Breastfeeding self-efficacy has been found to directly influence breastfeeding practices, and high breastfeeding self-efficacy was found to play a critical role in exclusive breastfeeding duration in the South African setting [12].

Qualitative findings from urban South Africa reported that infant nutrition interventions in this context should focus on: fostering maternal self-efficacy around exclusive breast-feeding; challenging mixed feeding practices and encouraging more responsive feeding approaches; and engaging family members to promote supportive household and community structures around infant feeding [13].

Promotion of early learning through play

Infant movement behaviours have been identified as important for early childhood growth and development [14]. Three categories of movement behaviours are of key importance: physical activity, sleep, and sedentary behaviours [14]. These three behaviours are contingent on each other to make up a 24-hour day, yet have independent health implications and thus

For peer review only - http://bmjopen.bmj.com/site/about/guidelines.xhtml

independent recommendations, which should all be met for optimal health. Guidelines for these behaviours in the first few years of life encourage tummy time and active play, sufficient and regular sleep, and minimisation of activities that require infants to be restrained (eg: car seats, strollers, walkers) [14-16]. Play is defined as intrinsically motivated activities done for recreational purposes and enjoyment. Play is how children learn, explore, engage in physical activity and motor development, develop socially, emotionally and cognitively, and bond with caregivers [17 18]. Outdoor play is particularly encouraged due to its beneficial effects on both physical activity and development [19]. Additionally, interactive and stimulating play (such as reading, singing, and playing with other adults or children) is encouraged to promote development [20], and also supports early learning [6].

Formative research in South Africa has determined that higher intensity movement was associated with earlier attainment of developmental milestones [21], and with taller stature and lower abdominal adiposity [22]. However, the majority of infants in South Africa were not meeting recommended 24-hour movement guidelines [23]. When using synchronous 24-hour accelerometery worn by mothers and infants, we showed that increased physical activity among mothers while caring for their infants was associated with increased infant movement during the same period of time [24]. This highlights the important role of the mother in infant movement behaviours, and thus growth and development. Interactive play is particularly important for promotion of parent-child bonding, offering an ideal opportunity for parents to engage with their children through stimulating activities which encourage movement, and in turn promote cognitive, emotional and social development. Stimulation interventions in LMICs have been shown to improve home caregiving environments, mother-child interactions, and maternal health literacy around ECD [25].

Qualitative research examining maternal perceptions of the importance of play in infancy reported that mothers in South Africa perceive developmental attainment as a key child outcome, indicating that interventions in this context should focus on promoting child development [26]. Mothers also reported significant barriers to encouraging play; including financial constraints, safety concerns and lack of certainty about how to promote play. Importantly, they did not perceive infant movement behaviours as behaviours that could be, or needed to be changed [26]. Additionally, and similar to findings from national reports, mothers reported not reading to their children, and most children in this context did not attend creche, indicating the importance of intervening in the home environment to improve opportunities for early learning [26 27].

Supporting maternal mental health

In South Africa, up to 57% of mothers present with symptoms of postnatal depression [28]. Qualitative studies have shown that in Soweto, household centred issues impacted upon the amount of chronic anger, stress, depression and suicidal ideation as well as material and relational hardships experienced by women [29]. Relational stress is exacerbated by the fact that mothers and children in South Africa live in multiple-generational households (66%), while only 21% are said to live in

For peer review only - http://bmjopen.bmj.com/site/about/guidelines.xhtml

households that may be defined as nuclear [27]. Women in Soweto are also exposed to high unemployment rates, low salaries, rife domestic violence [28], and thus social vulnerability [30].

Infants of mothers with depressive symptoms have an increased chance of developmental delay in the first 12 months of life [31]. In South Africa, a significant increase in psychological challenges at two years of age was evident when maternal postpartum depression was present [31]. Additionally, within the context of high exposure to trauma in South Africa, post-traumatic stress disorder in mothers was significantly associated with poorer infant developmental outcomes in fine motor and adaptive behaviour, as well as emotional regulation [32 33]. Due to the high prevalence of poor postpartum mental health in South Africa, and lack of support available for these women, screening of mothers for postpartum depression, anxiety, and post-traumatic stress disorder is recommended [28].

Responsive caregiving

Responsive caregiving is essential in helping children to thrive. Responsive caregiving refers to the encouragement of caregivers to make eye contact, to smile, cuddle and praise infants. Responsive parenting urges caregivers to notice their children's cues of hunger, illness, emotional distress, and signs of pleasure; and to respond sensitively and appropriately [20], while also allowing the child to independently explore and self-regulate. Responsive caregiving is therefore important for ensuring better nutrition and feeding practices, health practices, and development. Responsive caregiving ultimately leads to improved bonding between mother and infant. In a well bonded relationship, the mother aims to meet her infant's needs and the infant responds to having its needs met. Bonding serves to generate the feeling of connectedness between the dyad [34], and is crucial for developing a secure infant attachment.

A recent systematic review and meta-analysis of parenting interventions to improve ECD in children under the age of three has shown that interventions with responsive caregiving components had greater effects on child cognitive development, as well as on parenting practices and parent-child interactions [35]. The authors recommended that future interventions should aim to directly support parent responsiveness and sensitivity.

Tools for supporting parents in promoting these behaviours

The early infant caregiving environment is thus crucial in the formation of parent-child relationships, mother-infant synchronicity and contingency, behaviour development, and therefore child outcomes [20 36 37]. Mothers who are supported in their transition to parenthood have higher self-efficacy, and are less likely to develop past-partum depression [35 38]. Infants

For peer review only - http://bmjopen.bmj.com/site/about/guidelines.xhtml

who have confident and healthy mothers are more likely to develop resilience and emotional and cognitive competencies, to have healthy growth trajectories, and to develop healthy behavioural patterns [39].

Maternal self-efficacy is a likely contributor to responsiveness and sensitivity, and is determined by a mother's belief in her ability to care for her child, her emotional and psychological health, her child's temperament and health, and various environmental factors. Strategies to improve self-efficacy in parenthood include improving health literacy, reminding mothers of their natural ability to care for their child, social support, and promotion of mental health. Interventions that are targeted and focus specifically on changing maternal behaviour are most effective [40]. Maternal mental health should be supported through integration into ECD interventions and programmes, yet a lack of studies on maternal mental health that report on child health and development outcomes, in addition to mental health outcomes, has been identified as a critical gap [6]. Other gaps include identifying the most effective responsive caregiving interventions in LMICs, combining nutrition and caregiving strategies [6], and identifying ways to improve parenting behaviours without undermining self-efficacy.

In the first 3-months postpartum, tools to sensitise mothers to infant's competencies and to promote positive and nurturing interactions between caregivers and infants are successful in reducing postpartum depression, and increasing self-efficacy in both parents and health care providers [16 41]. Following early supportive intervention and sensitisation to infant behavioural cues, mothers benefit from continued support around breastfeeding, and promotion of healthy growth and development [16]. This can include provision of content to improve health literacy, practical tools and activities that promote infant development, continued sensitisation to infant behaviours and mother-infant interaction styles, and mental health promotion.

Behavioural feedback is an important and effective component of parenting interventions and behaviour change interventions in general [42], which can help to enhance parenting skills and thus child outcomes [41 43]. Feedback consists of comparing individual behaviour to a standard, and is thought to change behaviour by changing the individual's locus of control and focusing attention on a particular behaviour thus regulating that behaviour [44-46]. Feedback on parenting behaviours can be directive and/or responsive to behaviours in real-time, or following review of video recorded interactions [43 47]; and encouraging parents to practice skills following feedback is one of the strongest and most consistent predictors of improved parenting outcomes in meta-analyses [40 41]. Additionally, tailoring behavioural feedback is particularly effective for behaviour change in general [48].

Aims

This protocol outlines a phase 1 trial with the following aims:

For peer review only - http://bmjopen.bmj.com/site/about/guidelines.xhtml

1. To test the efficacy, feasibility, and acceptability of an intervention designed to promote development through encouraging maternal self-efficacy using behavioral feedback, content provision and mental health support.
2. To test the efficacy, feasibility, and acceptability of providing content, support, and referrals to promote breastfeeding self-efficacy
3. To test the efficacy, feasibility, and acceptability of providing content, and increasing opportunities for early learning through play to promote health literacy

Hypotheses

1. Using behavioural feedback and content provision to support mothers in their ability to be responsive, promote early learning in the home, understand and engage with their infants' health behaviours (specifically movement behaviours interactions), in conjunction with supporting mothers' mental health, will result in improved maternal self-efficacy, and therefore better infant development in the first year of life.
2. Supporting mothers with breastfeeding content, guidance, and referrals to lactation specialists in the first 6 months postpartum will increase self-efficacy and thus improve breastfeeding exclusivity
3. Proving mothers with access to resources, content, and activities to support their infant's play and development will increase their health literacy and thus encourage play and promote development

Methods and analysis

The Play Love And You (PLAY) study was developed with the overriding theme that all an infant really needs are opportunities for movement and development (play), responsive and interactive caregiving (love), and the mother herself (you).

Study design

The PLAY study was designed following the Standard Protocol Items (Recommendations for Interventional Trials (SPIRIT) 2013 statement) [49]. Mother-infant pairs (n=210) will be recruited starting in the third quarter of 2022 until the second quarter of 2023, and individually randomised into two groups (total n after expected attrition =150, n=75 in each group) using simple 1:1 randomization generated by Stata V.17. The intervention will last from birth to 12 months, with main assessments made when the infants are 0-, 6-, and 12-months of age. The trial will consist of a standard of care arm and an intervention arm – see Figure 1. Data collection teams will be blinded to randomisation at baseline and during primary outcome assessments, and separate data collection teams will collect baseline compared to primary outcome data. Blinding will be maintained by the data manager and broken only for *a priori* defined analyses (Figure 2). Randomisation into either group will be generated by the data manager only after recruitment and baseline assessments are completed, whereafter participants will be assigned to the intervention delivery teams accordingly (intervention delivery teams will thus not be blinded). Intervention delivery

For peer review only - http://bmjopen.bmj.com/site/about/guidelines.xhtml

teams will work separately and independently of each other, and of data collection teams. The trial is due to be completed in the second quarter of 2024.

Participants

We will recruit 210 mother-infant pairs within 3-10 days following delivery at community clinics in Soweto. Soweto is an urban-poor area in the City of Johannesburg covering 200 km^2 with over 1.3 million people (6400/km^2). Mother-infant pairs will be eligible to participate if:

- Mothers agree to participate for the duration of the study
- They reside in Soweto and plan to remain in Soweto for the study duration
- The mother is aged >=18 years
- The mother is the primary caregiver

Trial interventions

Standard of care arm

Mothers assigned to the standard of care arm will receive the Road to Health booklet as well as the Side-by-Side services provided by the National Department of Health as routine postpartum care. This content is divided into 5 knowledge pillars (nutrition, love, protection, healthcare, and extra care), and functions to record child's growth and health interventions, provide information for caregivers, and encourage collaboration between healthcare workers and caregivers (https://sidebyside.co.za).

Additionally, they will be contacted telephonically or in person every 2 months to check in, and to provide referrals to health care facilities and community services if necessary; and will have anthropometric, development, movement and interaction measurements taken every 4 months.

The standard of care arm will therefore include:

- Road to Health booklet as well as the Side-by-Side services provided as routine care postpartum at delivery
- Telephonic or in person check in every 2 months, and referrals to health care facilities and community services if necessary
- Measurements taken every 4 months.

Intervention arm

Mothers assigned to the intervention arm will receive everything as per the standard of care arm. Additionally, they will receive various micro-interventions according to the following key intervention themes: 1) exclusive breastfeeding for 6-months, 2) interactive play and promotion of early learning, 3) responsive caregiving, and 4) maternal mental health. Each

For peer review only - http://bmjopen.bmj.com/site/about/guidelines.xhtml

intervention theme will be prioritized during different phases of the trial and supported by components from the other themes. Figure 2 details when each theme will be prioritized and which supporting interventions will be included. Interventions will be delivered telephonically or in person, and via a mobile App created for the purposes of this trial. Interventions are detailed below:

Behavioral feedback

At recruitment, mothers will have the Newborn Behavioural Assessment (NBO) [50] administered by trained research staff. The NBO consists of 18 neurobehavioral observations along four dimensions — autonomic, motor, state organization and attentional-interactional - ultimately yielding a profile of the infant's behavioural repertoire. Importantly, the NBO allows for provision of infant specific anticipatory guidance, thus engaging parents in discussions around nurturing care, mother-infant interaction, play and development, and breastfeeding. The NBO has been shown to improve maternal sensitivity, and to promote positive maternal-infant interactions [50].

Every 4-months mothers will receive rapid feedback via the PLAY Study App and in person on their infant's and their own movement behaviours and on their interaction styles with their infant. Feedback will be based on objective measurement of infant movement behaviours and *headcam* video footage of interactive play in the home environment. Feedback on movement behaviours will consist of a graphical representation of the infant's and the mother's movement behaviours over the previous week, with personalised information and advice about: sleep routines, play and tummy time, nap times, daily activity routines, peaks of high activity vs lulls in activity, and recommended daily movement guidelines. This feedback is designed to sensitise mothers to their infant's behaviour patterns and to encourage healthy infant movement and routines, thus improving their self-efficacy to promote healthy routines for development. An example of the graphical feedback presented is included in Supplementary Figure 1. Feedback on mother-infant interactions during play is guided by the VIPP-SD [51] and will consist of a video segment from a peak interaction moment with mother and infant perspectives presented side-by-side, with personalised information and guidance on: positive interactions at 4 months (baby smiling, eye contact, soothing, directing attention, reinforcing mothers strength, communication and bonding); perspective taking at 8 months (describing and narrating the interaction, seeing baby as an individual, understanding baby cues), and recognising child feedback at 12 months (sensitivity and responsiveness, following cues, adjusting behaviour, soothing styles, interaction chains). This feedback is designed to promote positive and nurturing interactions and improve maternal self-efficacy. The App will contain the graphical and video feedback, and will also allow mothers to observe changes in their infant's behaviour and development over the trial period.

Mental health screening and support

At recruitment, and at 4 months mothers will complete the Edinburgh Postnatal Depression Screening questionnaire (EPDS), the Post-traumatic stress syndrome Checklist 5 (PCL-5), the Adverse Childhood Experiences Scale (ACES), and the

For peer review only - http://bmjopen.bmj.com/site/about/guidelines.xhtml

Antenatal Stress questionnaire (ASQ). Women who score in the high-risk category on one or more of these questionnaires will receive a phone call to assess any immediate risk, and an individual counselling session from a licensed psychologist, followed by referral and continued support as necessary. Women who score at medium risk on one or more of these questionnaires will receive referrals from the licensed psychologist and continued support as necessary, and uptake of this support will be monitored. All participants will receive a flyer on mental health and available services and support upon completion of the questionnaires.

Health literacy content delivered via the PLAY Study mobile App

Every week, health theme specific literacy content will be delivered via the PLAY Study mobile App, which was designed for the purposes of this intervention. The App creates a password protected profile for each participant and houses a section to deliver the feedback when available, as well as the weekly (and historic) content. This content is loaded to the profile every Monday, and participants can choose to receive push notifications to alert them of new content. Participants can engage with the App at any time, for as long as they wish, and as many times as they wish during the intervention. The weekly content will appear in the format of quick and easy tips, short videos, infographics, or ideas for home-based activities. Mothers will have continuous access to the App with all relevant resources, activities, and tools to support their interactions with their infant.

Health literacy content delivered telephonically or in person

Every two months theme focused content will be delivered telephonically or in person. Telephonic or in person visits will also follow the key intervention themes schedule; but will allow mothers to guide the conversation by using open questions and providing anticipatory guidance. Referrals to lactation specialists or psychologists or other services will be provided as necessary.

Health literacy content themes will be scheduled as follows and according to Figure 2.

- From 0-6 months content will be designed to increase breastfeeding self-efficacy and therefore promote exclusive breastfeeding
- From 6-12 months content will be designed to increase health literacy about play and development by providing content and activities to support and therefore improve these outcomes
- From 0-12 months there will be additional content to support maternal mental health, responsive caregiving and bonding to increase self-efficacy and therefore improve infant development.

All content will be guided by a broad range of pre-existing content such as UNICEF resources (https://www.unicef.org), and WHO recommendations [6], and other evidence based online resources. Contextually relevant material or adaptations to existing content will also be co-developed with a community advisory group (CAG).

For peer review only - http://bmjopen.bmj.com/site/about/guidelines.xhtml

The intervention arm will thus include the standard of care activities and:

- NBO administered at recruitment with feedback and anticipatory guidance
- Rapid behavioral feedback with individualized guidance on infant's movement behaviours and mother-infant interaction styles
- Mental health screening with referrals and individual support as needed for high-risk mothers
- Health literacy content
    - Weekly theme focused content delivered via the App
    - Telephonic or in person conversations with discussions around key themes every two months and referrals as necessary

Intervention delivery

The intervention has been informed by the UK MRC Guidelines for Complex Interventions, and is grounded in behaviour change techniques developed by Abraham and Michie [42]. We used the TIDieR (Template for Intervention Description and Replication) Checklist. Figure 3 details the logic model to illustrate the pathways to impact that guided the intervention approach. The agents of change are community health helpers (HHs), who do not need to have any specific qualification or expertise in health promotion but will be trained by the study team in community health promotion, as well as relevant intervention methodology. The HHs are supported by a supervisor with access to a referral network.

The intervention will be delivered by a combination of phone calls, in person visits, app-based health literacy content, personalised behavioural feedback and guidance; as well as individual counselling sessions for high-risk mothers. The content delivery will be guided by a schedule following key themes according to infant age and developmental stage. Phone calls and in person visits will be guided by a conversation flow chart, which will start with HHs asking mothers how they are doing and gently introducing the theme for discussion using open-ended questions. The flow chart will be designed to allow HHs to either ask follow up questions, or to provide anticipatory guidance and direct them to relevant resources in the App based on mothers' individual responses (example provided in Supplementary Figure 2). This will allow mothers to guide the conversation, while still following key themes for discussion. HHs will refer mothers as necessary if any concerns are raised during these conversations.

Outcome measures

Data collection will be conducted by independent, trained research assistants. The primary outcome measure is efficacy of the intervention in improving maternal self-efficacy and infant development at 12 months. Secondary outcomes will estimate efficacy of the intervention in improving infant development at 12 months, and will assess feasibility and acceptability of the intervention at 6-, and 12-months, as well as interim outcomes to determine mechanisms of impact (Figure 2).

For peer review only - http://bmjopen.bmj.com/site/about/guidelines.xhtml

Primary outcome at 12 months

- Maternal self-efficacy at 12 months compared between intervention and control groups

Secondary outcomes at 6 months

- Breastfeeding self-efficacy from 0-6 months compared between intervention and control groups
- Exclusive breastfeeding from 0-6 months compared between intervention and control groups
- Feasibility and Acceptability of the breastfeeding intervention

Secondary outcomes at 12 months

- Infant development at 12 months compared between the intervention and control groups using the Ages and Stages Questionnaire (ASQ3)
- Feasibility and Acceptability of the health literacy and behavioural feedback intervention
- Change in infant weight to length ratio compared between intervention and control groups
- Infant fat mass index (assessed using DXA fat mass divided by length) compared between intervention and control groups
- Change in maternal health literacy from 6-12 months compared between intervention and control groups
- Change in maternal mental health from 0-12 months compared between the intervention and control groups
- Infant movement behaviours compared between intervention and control groups
- Mother-infant interaction styles compared between intervention and control groups

Data collection

Primary outcome measure

Maternal self-efficacy will be assessed using the perceived maternal parenting self-efficacy tool (PMP S-E) at 6 and 12 months. This questionnaire assesses four subscales of self-efficacy: caretaking procedures, evoking behaviour, reading or signalling behaviours, and situational beliefs. While this tool was originally designed for assessing mothers' perceptions of their ability to understand and care for their hospitalized preterm neonates as well as being sensitive to the various levels and tasks in parenting [52]; it has since been successfully utilised in older, healthy infants in low income settings [53]. It has also been piloted in a sample of mothers from Soweto, providing a Cronbach's Alpha of 0.95 (data not yet published).

Secondary outcome measures

Infant development will be assessed at 12 months (and at 2, 4, and 8 months) using the Ages and Stages Questionnaires (ASQ3). These questionnaires assess development in five domains: communication, gross motor, fine motor, problem-solving,

For peer review only - http://bmjopen.bmj.com/site/about/guidelines.xhtml

and social-personal. The ASQ3 has been successfully applied in community-based, low-resource settings, and has been found to be feasible in South Africa [54 55].

Acceptability of each phase of the intervention will be assessed based on the Theoretical Framework of Acceptability [56]. This framework includes the following criteria: Affective Attitude, Burden, Ethicality, Intervention coherence, Opportunity costs, Perceived Effectiveness, and Self-Efficacy. These criteria will be assessed by means of questionnaires specific to each intervention theme, followed by focus group discussions (FGDs) in a sub-sample of participants to expand upon core themes identified.

Feasibility of each phase of the intervention will be assessed by determining whether the developed intervention is implementable in this context, has potential to work, as well as how it could be improved for future use and in other settings. We will follow the MRC guidance on process evaluation of complex interventions [57]. Evaluation will focus on implementation, mechanisms of impact, and context.

- Compliance will be assessed by counts (e.g.: login frequency on app, delivery of messages, and monitoring of phone calls using a log), and by discussions with the HHs.
- Intervention dose will be monitored by recording the frequency and duration of contact as well as the topics of conversation between participants and HHs or feedback given to participants using the logs.
- To understand the mechanisms through which the intervention brings about change in specified outcomes, women's experiences of receiving the intervention, contact with HHs, and the App will be assessed using questionnaires and focus groups at the close of each intervention phase. We will also use the secondary outcomes (process data) to determine whether individual proposed pathways of impact are effective prior to analysing trial outcomes.
- In considering the context, identification of factors that might act as barriers or facilitators to intervention implementation or effects will be assessed. This will include local and national policy, local service configuration and provision, and socio-demographic and environmental factors of the participants. Our approach to assessing context will be through a combination of policy mapping, quantitative data collection, and longitudinal consultation with the community advisory group.

Maternal breastfeeding self-efficacy will be assessed every two months from 0-6 months using the breastfeeding self-efficacy short form (BSES-SF) [58]. This comprises of 14 questions, each with a five-point Likert scale response scale ranging from 1=not at all confident to 5=very confident. The higher the final aggregate score (which has a range of 14 to 70) the higher the level of breastfeeding self-efficacy.

For peer review only - http://bmjopen.bmj.com/site/about/guidelines.xhtml

Exclusive breastfeeding will be assessed every two months form 0-6 months by asking mothers whether they are currently breastfeeding and if not, at what infant age did they stop breastfeeding and why they stopped breastfeeding. They will also be asked if they are giving their infant anything other than breastmilk, and if so, why.

Weight and length will be measured by trained research staff at baseline and 12 months (and at 4- and 8 months). Weight:length ratio (WLR; kg/m) will then be calculated to estimate anthropometric changes in body composition. At 12-months, Dual X-Ray Absorptiometry (DXA) scans will be performed by a trained technician. Whole-body measurements of fat mass and fat-free mass will be extracted for use in analyses. Fat mass index (FMI; kg/m^3) will be calculated from these estimates to describe adiposity.

Health literacy will be assessed at 6- and 12-months using the parent version of the Health Literacy Questionnaire (HLQ) [59], which covers nine areas of health literacy including functional health literacy and has been validated in high- and low-to-middle-income countries.

Various aspects of maternal mental health will be assessed at baseline, 4- and 12 months. The Post-traumatic Stress Disorder Checklist (PCL-5) [60] will be administered whereby rating scale descriptors include: "Not at all," "A little bit," Moderately," "Quite a bit," and "Extremely." A total symptom severity score is calculated by adding up the item scores which range from 0–80. The cut-off for PTSD that will be used in this study was 31. The Edinburgh Postnatal Depression screen (EPDS) [61] will be used to detect postnatal depression. Scores for each item range from 0 to 3 on a Likert scale, and are added together to obtain an overall total. Scores of 7-13 indicate mild depression, 14-18 moderate and 19-30 severe depression. The Adverse Childhood Experience questionnaire (ACE) [62] will assess childhood abuse, neglect, and household challenges. A score from 0- 10 is calculated based on how many types of events were experienced before 18 years old. The Antenatal Stress Questionnaire (ASQ) will be administered to assess stressful life events. An additional nine items about social support will be included. The Postpartum Bonding Questionnaire (PBQ) [63] will be used to detect early mother–infant bonding. It consists of 4 subscales "impaired mother/infant bonding", "rejection and anger", "anxiety about care", and the "risk of abuse." These questions are rated on a 6-point Likert scale, ranging from 0 (always) to 5 (never).

Infant adherence to movement guidelines will be assessed every 4 months using accelerometery. Seven-day, 24-hour wrist worn accelerometery data (Axivity-AX3 worn on both mother and infant) will be collected, downloaded (omgui, Open Movement, UK), auto-calibrated, and processed. Data is summarised to generate average acceleration (mg) per 5-second

For peer review only - http://bmjopen.bmj.com/site/about/guidelines.xhtml

interval, as well as overall time (hourly and weekly) spent in varying intensities of activity, which will allow for determination of adherence to movement guidelines, and changes in activity levels over the intervention period.

Mother-infant interactions will be measured during play using first person observation and behavioural analysis every 4 months. Mother-infant interactions have primarily been measured using questionnaires or third party observation, yet these measures are limited by their subjectivity and intrusiveness. First-person observation using *headcams* – small cameras which are attached to headbands on both infants and mothers for a period of time while interacting at home – provide a first person view of the interaction from both infant and mother perspectives with more naturalistic and detailed measures. These *headcams* have been piloted in South Africa, and were found to be largely acceptable and feasible in this context [64]. *Headcam* measures will be collected during at least five, 3-5 minute play interactions over the course of a week. *Headcam* observations will be coded using Noldus Observer XT software. An event-based coding framework will be used to categorise second-by-second key maternal and infant behaviours, i.e.: touch, play, vocalisations, and gaze. This allows for determination of the probability of certain behaviour preceding or following infant play. By combining measures of movement behaviours and mother-infant interactions; intensity and frequency of movement during interactive play, as well as interaction styles during play will be determined for feedback to mothers.

Patient and public involvement

Prior to trial implementation, a community advisory group (CAG) (n=16) of mothers from Soweto with infants aged 0-3 months will be established to serve as experts on the lived experience of being a mother to an infant in Soweto. For about half of these women, family matriarchs will be recruited to form a separate CAG (n=8). Research from this context has indicated that family matriarchs play an important role in the choices that mothers make around infant feeding and nutrition, and as such it is pertinent they be included in interventions targeting breastfeeding and other child care practices.

Intervention content and activities, and behavioural feedback will be tested and/or co-developed with the CAG, using focus group discussions and piloting of intervention content and feedback. Thus, acceptability and feasibility of the intervention content will be tested by the CAG in order to understand any changes required and thus refine the intervention accordingly.

The CAG will continue to advise throughout the duration of the intervention, via regular (every 4 months) focus group discussions (FGDs) where the research team will raise queries about intervention content, delivery and uptake, retention strategies, and feedback mechanisms. Additionally, the CAG will provide important insight into their lived experience of

For peer review only - http://bmjopen.bmj.com/site/about/guidelines.xhtml

being a mother in Soweto over time and as their infant grows. This will provide useful insight to guide intervention conversations, and to better understand the context within which the intervention is being delivered.

Monitoring

Monitoring of intervention activities will be done throughout the trial. The delivery of each contact (duration, date, content) will be recorded using a monitoring log in order to be able to monitor intervention intensity and frequency. Engagement with the App content and behavioural feedback (i.e.: number of page visits per day, and duration of time spent on the App) will be monitored via the App.

Adherence will be assessed every 4 months, and strategies to improve adherence will be discussed with the CAG as necessary. Additionally, the research team will meet with HHs weekly to determine any concerns or issues with compliance or retention, and strategies will be discussed accordingly. Any adverse events will be recorded and reported as necessary according to standard operating procedures.

Sample size

Given expected attrition of 30%, recruiting 210 mother-infant pairs should yield a final sample size of 150 mother-infant pairs, which will be divided into the two groups (n=75 each). A sample size of 150 mother-infant pairs divided into two groups (n=75 each; control and trial group) will allow for 99% power to detect efficacy of the intervention based on maternal self-efficacy data assessed in the piloting phase of the behavioural feedback intervention (data not yet published).

Attrition will be minimised by maintaining regular contact with both groups, by recruiting participants who plan on remaining in Soweto, and by using a flexible and versatile delivery approach including in-person visits, app based content, telephonic sessions, mobile messaging, and site visits.

Data analysis plan

The efficacy of the intervention will be assessed using intention to treat analysis. Secondary outcome data will be compared between the intervention and standard of care arms using Student's unpaired t-tests. Focus will be placed on the primary efficacy outcome (self-efficacy) due to limited power for secondary outcomes, which will therefore be treated with caution and presented using point estimates and precision (95% CI). Sensitivity analyses will include comparing baseline characteristics between intervention and standard of care arms, examining the effect of intervention dose on outcome data, and stratifying analyses by sex.

For peer review only - http://bmjopen.bmj.com/site/about/guidelines.xhtml

The feasibility and acceptability of the intervention at each phase will be assessed using qualitative and quantitative methods. FGDs will be transcribed and coded line by line, and data will be grouped according to common themes. Themes will be sorted into sub-themes and continually reviewed to identify an overarching narrative to identify key findings from each FGD.

Data management

REDCap will be used for data management (https://www.project-redcap.org). Secure Sockets Layer (SSL) provides the needed security for the REDCap similar to what a VPN will provide. The Wits University server hosting REDCap is resilient and hardened against hacking and other forms of attacks. All data traffic is encrypted, and an installed network firewall provides secure network access. The REDCap system provides inherent application-level security including access controls and comprehensive audit trails; and all incoming requests get intentionally filtered, sanitized and escaped to prevent against attacks.

Data files and metadata will be uploaded and stored on REDCap by the project data manager, on a password protected Wits server for the duration of the project to enable team access. REDCap is used for data and metadata storage, as well as for data collection. Data will be inputted online with secure web authentication, data logging and SSL encryption. Accelerometery files and video files, as well as focus group discussion notes and transcripts will be stored on a secure and encrypted laptop, as well as on an external hard drive, and backed up as meta-data to REDCap. Regular back-up copies will be made by the project data manager on password protect file space. When the project is completed, the full database will be stored on both DPHRU and University servers. The items and metadata on the institutional repository are backed up on a daily basis and these backups are kept on tape. All data collection devices will be encrypted.

Data from this study will be stored for a minimum of 10 years from the completion of the study in order to allow for any verification of data. Long-term data storage will be in .csv files using a standard (simple) ASCII coding scheme in line with UK Data Service recommendations:

(https://www.ukdataservice.ac.uk/manage-data/format/recommended-formats).

Ethics and dissemination

Ethical approval has been granted for the establishment of the community advisory group (M210846), and for the trial (M220217) from the Human Ethics Research Committee of the University of the Witwatersrand, Johannesburg, South Africa. Additional approval has been granted by the Research Committee of Johannesburg Health District (GP_202202_021). Participants will be provided with an information sheet and required to provide written consent (Supplementary Document

For peer review only - http://bmjopen.bmj.com/site/about/guidelines.xhtml

1) prior to being enrolled. Study results will be shared via publication in peer-reviewed journals, conference presentation and media engagement.

Discussion

If found to be feasible, acceptable and successful, these findings could lead to implementation of the intervention on a larger scale. Particularly, implementing intervention content within primary health care services, and linkage with existing digital solutions (like MomConnect in South Africa), would allow for dissemination of the health literacy components of this intervention nationally. Furthermore, the PLAY Study App has the potential to be developed further and made into an accessible App for all mothers. All of this content aligns with the Nurturing Care Framework and WHO guidelines and is therefore relevant at a national policy level, as well as internationally. Additionally, engagement with the community and dissemination of findings to participants and other stakeholders provides opportunities for knowledge mobilisation and therefore greater impact. There is potential to transfer the intervention to other LMICs and HICs, however since this content was designed with the community to be contextually relevant, adjustments would need to be made to ensure feasibility and acceptability in other contexts. Certain aspects of the intervention, such as the accelerometry and headcam assessments and feedback are more complex and would require funding and training to implement on a larger scale, yet the concept of providing feedback using some of the other assessments such as development and growth can feasibly be incorporated into digital platforms or the PLAY Study App for scaling up.

The PLAY study relies to some extent on the availability of cell phone or computer access for the delivery of intervention content, which may be limiting in the Soweto setting. However, we will attempt to mitigate this by making the app data free, by making the app available on multiple platforms (web, mobile, android, Apple etc), and by continuously consulting with the CAG to ensure we come up with appropriate solutions if we encounter lack of access. Additionally, the intervention content and delivery was designed with the Soweto community to be feasible and acceptable in a South African context, and adaptations would need to be made to ensure the same in other settings. Findings from this study are relevant to a socioeconomically disadvantaged group of mothers, and thus cannot be extrapolated to higher income settings or different population groups. This study does not specifically include fathers due to the low prevalence of caregiving fathers in this context. Future studies directed at both parents or at other caregivers would need to be adjusted for those population groups.

Acknowledgements

For peer review only - http://bmjopen.bmj.com/site/about/guidelines.xhtml

The authors would like to thank the Wellcome Trust and the NIHR for their financial support which made this study possible.

Contributors

AP is the principal investigator for the PLAY study. AP, SAN, RP, LR and DAL conceptualised the study design. AP wrote the initial draft of the protocol. SAN, RP, LR, DAL, DM, FB, SJC, and HT read and contributed to the final version. All authors provided edits and critiqued the manuscript for intellectual content.

Funding

This work was supported by the National Institute for Health Research (NIHR) (using the UK's Official Development Assistance (ODA) Funding) and Wellcome [222007/Z/20/Z] under the NIHR-Wellcome Partnership for Global Health Research. The views expressed are those of the authors and not necessarily those of Wellcome, the NIHR or the Department of Health and Social Care.

Competing interests

The authors have no conflicts of interest to report.

Patient consent for publication

Not required.

Provenance and peer review

Not commissioned; peer-reviewed for ethical and funding approval prior to submission.

For peer review only

References

1. Black MM, Walker SP, Fernald LCH, et al. Early childhood development coming of age: science through the life course. *The Lancet* 2017;389(10064):77-90. doi: 10.1016/s0140-6736(16)31389-7
2. Lu C, Black MM, Richter LM. Risk of poor development in young children in low-income and middle-income countries: an estimation and analysis at the global, regional, and country level. *The Lancet Global Health* 2016;4(12):e916-e22. doi: 10.1016/s2214-109x(16)30266-2
3. Collaborators LBDDBoM. Mapping local patterns of childhood overweight and wasting in low- and middle-income countries between 2000 and 2017. *Nat Med* 2020;26(5):750-59. doi: 10.1038/s41591-020-0807-6 [published Online First: 2020/04/22]
4. Aboud FE, Yousafzai AK. Global health and development in early childhood. *Annu Rev Psychol* 2015;66:433-57. doi: 10.1146/annurev-psych-010814-015128 [published Online First: 2014/09/10]
5. Cantor P, Osher D, Berg J, et al. Malleability, plasticity, and individuality: How children learn and develop in context1. *Applied Developmental Science* 2018;23(4):307-37. doi: 10.1080/10888691.2017.1398649
6. World Health Organization. Improving early childhood development: WHO guideline, 2020.
7. Victora CG, Bahl R, Barros AJD, et al. Breastfeeding in the 21st century: epidemiology, mechanisms, and lifelong effect. *The Lancet* 2016;387(10017):475-90. doi: 10.1016/s0140-6736(15)01024-7
8. World Health Organisation. World Health Organization (2003) Global strategy for infant and young child feeding. In: Organisation WH, ed. WHO Geneva, 2003.
9. Nieuwoudt S, Manderson L, Norris SA. Infant feeding practices in Soweto, South Africa: Implications for healthcare providers. *S Afr Med J* 2018;108(9):756-62. doi: 10.7196/SAMJ.2018.v108i9.13358 [published Online First: 2018/09/06]
10. Mushaphi LF, Mahopo TC, Nesamvuni CN, et al. Recommendations for Infant Feeding Policy and Programs in Dzimauli Region, South Africa: Results From the MAL-ED Birth Cohort. *Food Nutr Bull* 2017;38(3):428-40. doi: 10.1177/0379572117696662 [published Online First: 2017/09/30]
11. Goosen C, McLachlan M, Schübl C. Infant feeding practices during the first 6 months of life in a low-income area of the Western Cape Province. *South African Journal of Child Health* 2014;8(2) doi: 10.7196/sajch.675
12. Jama NA, Wilford A, Masango Z, et al. Enablers and barriers to success among mothers planning to exclusively breastfeed for six months: a qualitative prospective cohort study in KwaZulu-Natal, South Africa. *Int Breastfeed J* 2017;12:43. doi: 10.1186/s13006-017-0135-8 [published Online First: 2017/10/14]
13. Wrottesley SV, Prioreschi A, Slemming W, et al. Maternal perspectives on infant feeding practices in Soweto, South Africa. *Public Health Nutr* 2020:1-13. doi: 10.1017/S1368980020002451 [published Online First: 2020/08/11]
14. World Health Organization. Guidelines on physical activity, sedentary behaviour and sleep for children under 5 years of age. *Geneva: Licence: CC BY-NC-SA 30 IGO* 2019

For peer review only

15. Draper CE, Tomaz SA, Biersteker L, et al. The South African 24-Hour Movement Guidelines for Birth to 5 Years: An Integration of Physical Activity, Sitting Behavior, Screen Time, and Sleep. *J Phys Act Health* 2020;17(1):109-19. doi: 10.1123/jpah.2019-0187 [published Online First: 2019/12/27]
16. Tremblay M, Chaput J, Adamo K, et al. Canadian 24-Hour Movement Guidelines for the Early Years (0–4 years): An Integration of Physical Activity, Sedentary Behaviour, and Sleep. *BMC Public Health* 2017;17 (Suppl 5):874. doi: 10.1186/s12889-017-4859-6
17. Ginsburg KR, American Academy of Pediatrics Committee on C, American Academy of Pediatrics Committee on Psychosocial Aspects of C, et al. The importance of play in promoting healthy child development and maintaining strong parent-child bonds. *Pediatrics* 2007;119(1):182-91. doi: 10.1542/peds.2006-2697 [published Online First: 2007/01/04]
18. Markova G. The Games Infants Play: Social Games During Early Mother-Infant Interactions and Their Relationship With Oxytocin. *Front Psychol* 2018;9:1041. doi: 10.3389/fpsyg.2018.01041 [published Online First: 2018/07/11]
19. Tremblay MS, Gray C, Babcock S, et al. Position Statement on Active Outdoor Play. *Int J Environ Res Public Health* 2015;12(6):6475-505. doi: 10.3390/ijerph120606475 [published Online First: 2015/06/11]
20. World Health Organization, United Nations Children's Fund, Group. WB. Nurturing care for early childhood development: a framework for helping children survive and thrive to transform health and human potential. Geneva: World Health Organization, 2018.
21. Prioreschi A, Brage S, Hesketh KD, et al. Describing objectively measured physical activity levels, patterns, and correlates in a cross sectional sample of infants and toddlers from South Africa. *Int J Behav Nutr Phys Act* 2017;14(1):176. doi: 10.1186/s12966-017-0633-5 [published Online First: 2017/12/24]
22. Prioreschi A, KK O, E DLR, et al. Associations between abdominal adiposity, body size and objectively measured physical activity in infants from Soweto, South Africa. *Maternal and Child Health* 2022;In press doi: DOI: 10.1007/s10995-022-03406-5
23. Prioreschi A, Micklesfield LK. Compliance With Physical Activity and Sedentary Behavior Guidelines and Associations With Abdominal Adiposity in a Sample of Infants and Toddlers From Soweto, South Africa. *J Phys Act Health* 2019;16(12):872-79. doi: 10.1123/jpah.2018-0182 [published Online First: 2019/07/19]
24. Prioreschi A, Brage S, Westgate K, et al. Describing the diurnal relationships between objectively measured mother and infant physical activity. *Int J Behav Nutr Phys Act* 2018;15(1):59. doi: 10.1186/s12966-018-0692-2 [published Online First: 2018/06/27]
25. Jeong J, Pitchik HO, Yousafzai AK. Stimulation Interventions and Parenting in Low- and Middle-Income Countries: A Meta-analysis. *Pediatrics* 2018;141(4) doi: 10.1542/peds.2017-3510 [published Online First: 2018/03/04]
26. Prioreschi A, Wrottesley SV, Slemming W, et al. A qualitative study reporting maternal perceptions of the importance of play for healthy growth and development in the first two years of life. *BMC Pediatr* 2020;20(1):428. doi: 10.1186/s12887-020-02321-4 [published Online First: 2020/09/11]
27. Hall K, Richter L, Mokomane Z, et al. South African Child Gauge 2018. *Cape Town: Children's Institute, University of Cape Town ISBN: 978-0-620-81934-3* 2018

For peer review only - http://bmjopen.bmj.com/site/about/guidelines.xhtml

28. Mokwena K, Masike I. The Need for Universal Screening for Postnatal Depression in South Africa: Confirmation from a Sub-District in Pretoria, South Africa. *Int J Environ Res Public Health* 2020;17(19) doi: 10.3390/ijerph17196980 [published Online First: 2020/09/30]
29. Cohen E, Ware L, Prioreschi A, et al. Material and relational difficulties: The Impact of the Household Environment on the Emotional Well-Being of Young Black Women Living in Soweto, South Africa. *Journal of Family Issues* 2019;41(8):1307-32.
30. Ware LJ, Kim AW, Prioreschi A, et al. Social vulnerability, parity and food insecurity in urban South African young women: the healthy life trajectories initiative (HeLTI) study. *J Public Health Policy* 2021;42(3):373-89. doi: 10.1057/s41271-021-00289-8 [published Online First: 2021/05/21]
31. Verkuijl NE, Richter L, Norris SA, et al. Postnatal depressive symptoms and child psychological development at 10 years: a prospective study of longitudinal data from the South African Birth to Twenty cohort. *The Lancet Psychiatry* 2014;1(6):454-60. doi: 10.1016/s2215-0366(14)70361-x
32. Koen N, Brittain K, Donald KA, et al. Maternal posttraumatic stress disorder and infant developmental outcomes in a South African birth cohort study. *Psychol Trauma* 2017;9(3):292-300. doi: 10.1037/tra0000234 [published Online First: 2017/05/02]
33. Bosquet Enlow M, Kitts RL, Blood E, et al. Maternal posttraumatic stress symptoms and infant emotional reactivity and emotion regulation. *Infant Behav Dev* 2011;34(4):487-503. doi: 10.1016/j.infbeh.2011.07.007 [published Online First: 2011/08/25]
34. Hill R, Flanagan J. The maternal-infant Bond: Clarifying the concept. *International Journal of Nursing Knowledge* 2020;31(1):14-18.
35. Jeong J, Franchett EE, Ramos de Oliveira CV, et al. Parenting interventions to promote early child development in the first three years of life: A global systematic review and meta-analysis. *PLoS Med* 2021;18(5):e1003602. doi: 10.1371/journal.pmed.1003602 [published Online First: 2021/05/11]
36. Richter L. The importance of caregiver-child interactions for the survival and healthy development of young children: a review. *World Health Organisation* 2004
37. Bernard K, Meade EB, Dozier M. Parental synchrony and nurturance as targets in an attachment based intervention: building upon Mary Ainsworth's insights about mother-infant interaction. *Attach Hum Dev* 2013;15(5-6):507-23. doi: 10.1080/14616734.2013.820920 [published Online First: 2013/12/05]
38. Brazeau N, Reisz S, Jacobvitz D, et al. Understanding the Connection between Attachment Trauma and Maternal Self-Efficacy in Depressed Mothers. *Infant Ment Health J* 2018;39(1):30-43. doi: 10.1002/imhj.21692 [published Online First: 2017/12/28]
39. Leerkes E, Crockenberg S. The Development of Maternal Self-Efficacy and Its Impact on Maternal Behavior. *Infancy* 2002;3(2):227-47.
40. Dozier M, Bernard K. Attachment and Biobehavioral Catch-up: Addressing the Needs of Infants and Toddlers Exposed to Inadequate or Problematic Caregiving. *Curr Opin Psychol* 2017;15:111-17. doi: 10.1016/j.copsyc.2017.03.003 [published Online First: 2017/06/27]
41. Kaminski JW, Valle LA, Filene JH, et al. A meta-analytic review of components associated with parent training program effectiveness. *J Abnorm Child Psychol*

For peer review only

2008;36(4):567-89. doi: 10.1007/s10802-007-9201-9 [published Online First: 2008/01/22]

42. Michie S, van Stralen MM, West R. The behaviour change wheel: a new method for characterising and designing behaviour change interventions. *Implement Sci* 2011;6:42. doi: 10.1186/1748-5908-6-42 [published Online First: 2011/04/26]
43. Caron EB, Bernard K, Dozier M. In Vivo Feedback Predicts Parent Behavior Change in the Attachment and Biobehavioral Catch-up Intervention. *J Clin Child Adolesc Psychol* 2018;47(sup1):S35-S46. doi: 10.1080/15374416.2016.1141359 [published Online First: 2016/04/05]
44. Carey RN, Connell LE, Johnston M, et al. Behavior Change Techniques and Their Mechanisms of Action: A Synthesis of Links Described in Published Intervention Literature. *Ann Behav Med* 2019;53(8):693-707. doi: 10.1093/abm/kay078 [published Online First: 2018/10/12]
45. Larson EL, Patel SJ, Evans D, et al. Feedback as a strategy to change behaviour: the devil is in the details. *J Eval Clin Pract* 2013;19(2):230-4. doi: 10.1111/j.1365-2753.2011.01801.x [published Online First: 2011/12/02]
46. Michie S, Wood CE, Johnston M, et al. Behaviour change techniques: the development and evaluation of a taxonomic method for reporting and describing behaviour change interventions (a suite of five studies involving consensus methods, randomised controlled trials and analysis of qualitative data). *Health Technol Assess* 2015;19(99):1-188. doi: 10.3310/hta19990 [published Online First: 2015/12/01]
47. Van Zeijl J, Mesman J, Van IMH, et al. Attachment-based intervention for enhancing sensitive discipline in mothers of 1- to 3-year-old children at risk for externalizing behavior problems: a randomized controlled trial. *J Consult Clin Psychol* 2006;74(6):994-1005. doi: 10.1037/0022-006X.74.6.994 [published Online First: 2006/12/13]
48. Richards K, Enderlin C, Beck C, et al. Tailored Biobehavioral Interventions: A Literature Review and Synthesis. *Research and Theory for Nursing Practice* 2007;21(4):271-85.
49. Chan AW, Tetzlaff JM, Gotzsche PC, et al. SPIRIT 2013 explanation and elaboration: guidance for protocols of clinical trials. *BMJ* 2013;346:e7586. doi: 10.1136/bmj.e7586 [published Online First: 2013/01/11]
50. Nugent JK, Bartlett JD, Von Ende A, et al. The Effects of the Newborn Behavioral Observations (NBO) System on Sensitivity in Mother–Infant Interactions. *Infants & Young Children* 2017;30(4):257-68. doi: 10.1097/iyc.0000000000000103
51. van IMH, Schuengel C, Wang Q, et al. Improving parenting, child attachment, and externalizing behaviors: Meta-analysis of the first 25 randomized controlled trials on the effects of Video-feedback Intervention to promote Positive Parenting and Sensitive Discipline. *Dev Psychopathol* 2022:1-16. doi: 10.1017/S0954579421001462 [published Online First: 2022/01/18]
52. Barnes C, Adamson-Macedo E. Perceived Maternal Parenting Self-Efficacy (PMP S-E) tool: development and validation with mothers of hospitalized preterm neonates. J Adv Nurs 2007;60(5):550-60. doi: doi: 10.1111/j.1365-2648.2007.04445.x
53. Abuhammad S. Predictors of maternal parenting self-efficacy for infants and toddlers: A Jordanian study. PLoS One 2020;15(11):e0241585. doi: 10.1371/journal.pone.0241585 [published Online First: 2020/11/19]

For peer review only - http://bmjopen.bmj.com/site/about/guidelines.xhtml

54. Hsiao C, Richter L, Makusha T, et al. Use of the ages and stages questionnaire adapted for South Africa and Zambia. Child Care Health Dev 2017;43(1):59-66. doi: 10.1111/cch.12413 [published Online First: 2016/10/07]
55. van Heerden A, Hsiao C, Matafwali B, et al. Support for the feasibility of the ages and stages questionnaire as a developmental screening tool: a cross-sectional study of South African and Zambian children aged 2-60 months. BMC Pediatr 2017;17(1):55. doi: 10.1186/s12887-017-0802-3 [published Online First: 2017/02/18]
56. Sekhon, M., M. Cartwright, and J.J. Francis. Acceptability of healthcare interventions: an overview of reviews and development of a theoretical framework. B*MC Health Serv Res* 2017;17(1): p. 88.
57. MRC. Process evaluation of complex interventions: Medical Research Council guidance. *BMJ* 2015;350.
58. Dennis, C.L. The breastfeeding self-efficacy scale: psychometric assessment of the short form. *J Obstet Gynecol Neonatal Nurs* 2003;32(6): p. 734-44.
59. Osborne, R., et al., The grounded psychometric development and initial validation of the Health Literacy Questionnaire (HLQ). *BMC Public Health* 2013;13(658).
60. Blevins, C.A., et al., The Posttraumatic Stress Disorder Checklist for DSM-5 (PCL-5): Development and Initial Psychometric Evaluation. *J Trauma Stress* 2015;28(6): p. 489-98.
61. Cox, J.L., J.M. Holden, and R. Sagovsky. Detection of postnatal depression. Development of the 10-item Edinburgh Postnatal Depression Scale. *Br J Psychiatry* 1987;150: p. 782-6.
62. Felitti, V.J., et al., Relationship of Childhood Abuse and Household Dysfunction to Many of the Leading Causes of Death in Adults. The Adverse Childhood Experiences (ACE) Study. *American Journal of Preventive Medicine* 1998; 14(4): p. 245-258.
63. Brockington, I.F., C. Fraser, and D. Wilson. The Postpartum Bonding Questionnaire: a validation. *Arch Womens Ment Health* 2006; 9(5): p. 233-42.
64. Wrottesley, S.V., R. Pearson, and A. Prioreschi, The headcam mother-infant interaction assessment tool: testing the feasibility and acceptability in Soweto, South Africa, using participatory engagement. *Pilot Feasibility Stud* 2021; 7(1): p. 140.

For peer review only

Figures

Figure 1. Participant flow chart

Figure 2. Intervention themes and assessments

Figure 3. PLAY Study logic model

For peer review only - http://bmjopen.bmj.com/site/about/guidelines.xhtml

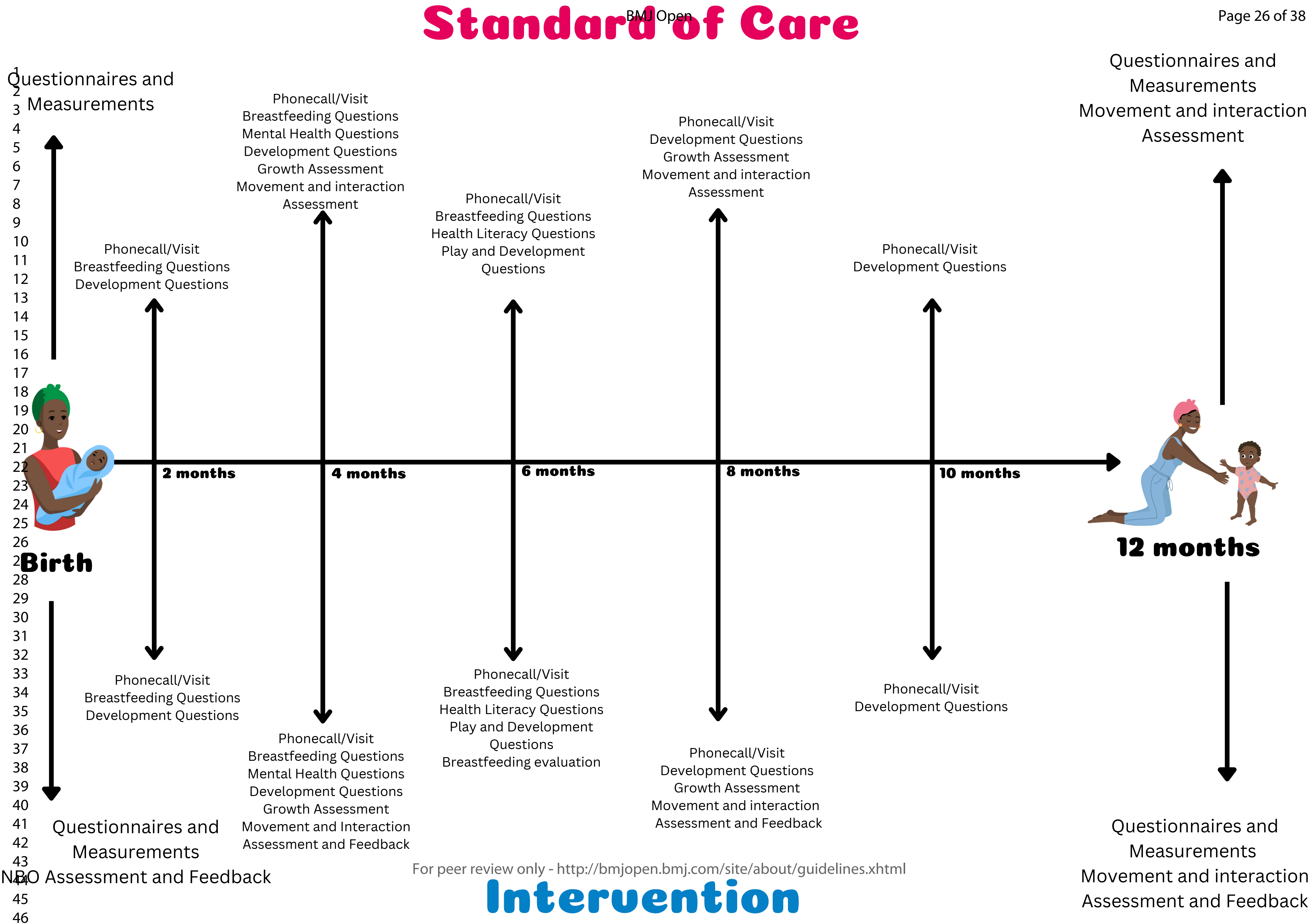
Standard of Care
Questionnaires and Measurements
Phonecall/Visit Breastfeeding Questions Development Questions
Phonecall/Visit Breastfeeding Questions Mental Health Questions Development Questions Growth Assessment Movement and interaction Assessment
Phonecall/Visit Breastfeeding Questions Health Literacy Questions Play and Development Questions
Phonecall/Visit Development Questions Growth Assessment Movement and interaction Assessment
Phonecall/Visit Development Questions
Questionnaires and Measurements Movement and interaction Assessment
Birth
2 months
4 months
6 months
8 months
10 months
12 months
Phonecall/Visit Breastfeeding Questions Development Questions
Phonecall/Visit Breastfeeding Questions Mental Health Questions Development Questions Growth Assessment Movement and Interaction Assessment and Feedback
Phonecall/Visit Breastfeeding Questions Health Literacy Questions Play and Development Questions Breastfeeding evaluation
Phonecall/Visit Development Questions Growth Assessment Movement and interaction Assessment and Feedback
Phonecall/Visit Development Questions
Questionnaires and Measurements NBO Assessment and Feedback
Questionnaires and Measurements Movement and interaction Assessment and Feedback
Intervention

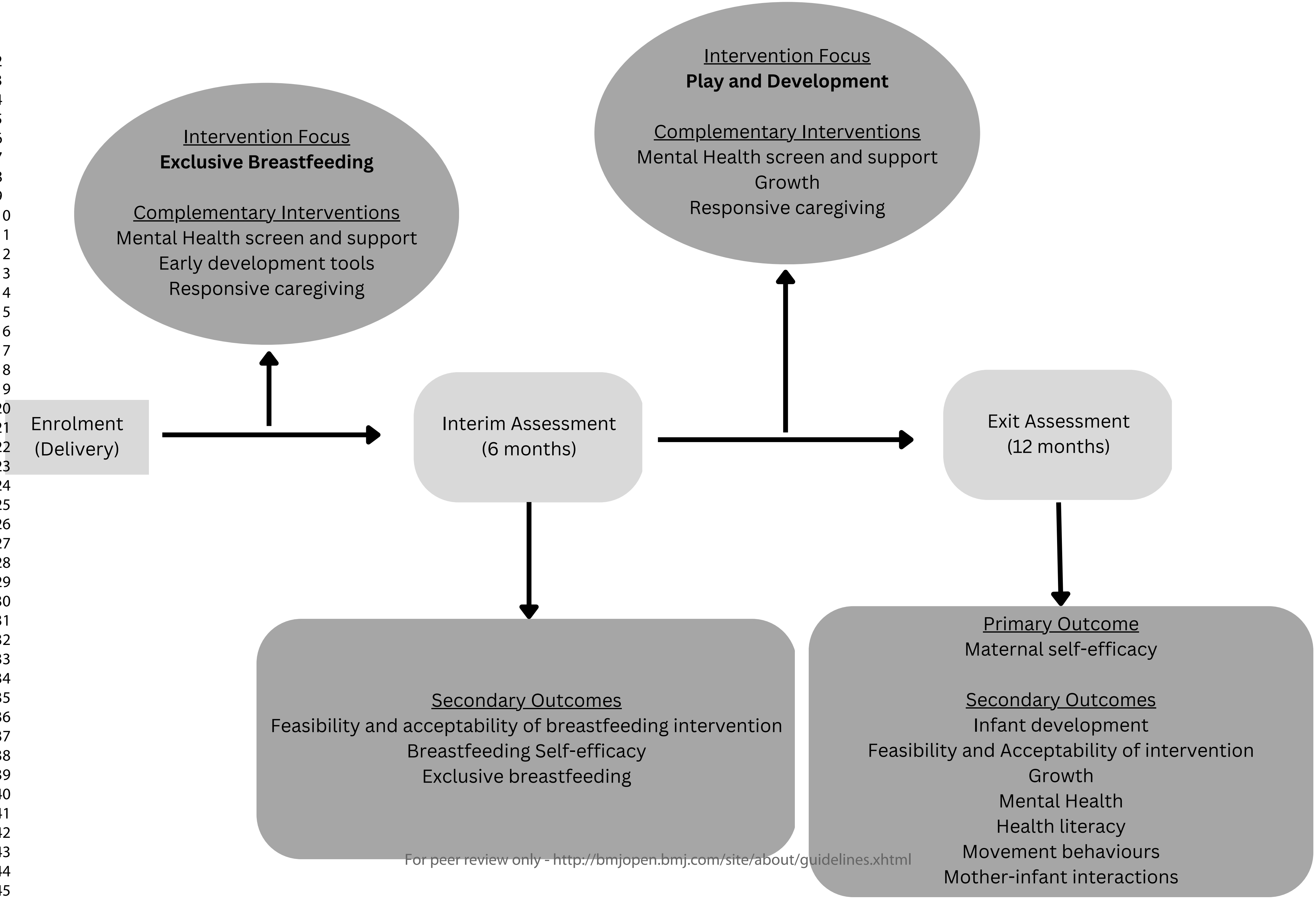

For peer review only - http://bmjopen.bmj.com/site/about/guidelines.xhtml

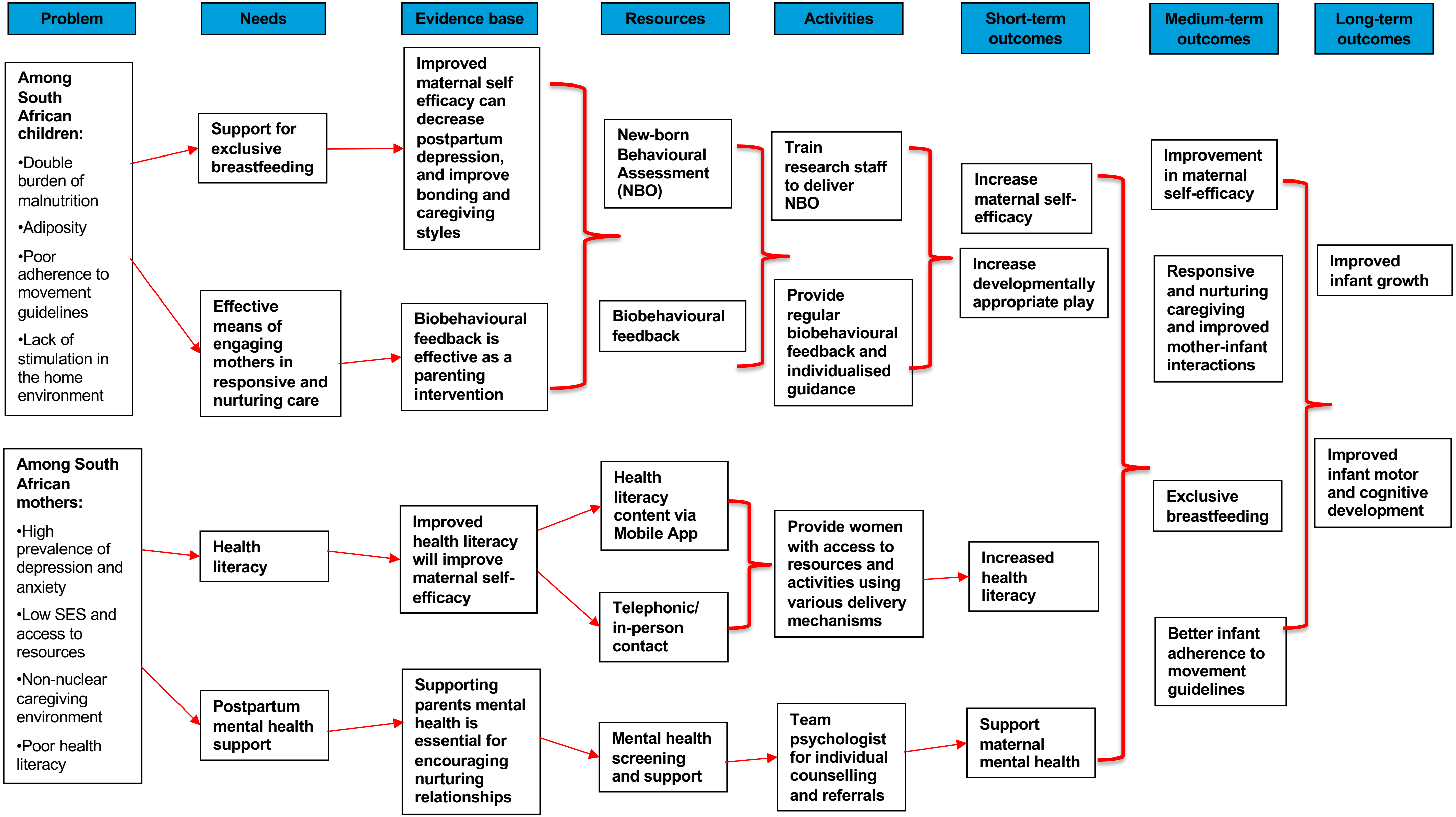
Problem
Needs
Evidence base
Resources
Activities
Short-term outcomes
Medium-term outcomes
Long-term outcomes
Among South African children:
•Double burden of malnutrition
•Adiposity
•Poor adherence to movement guidelines
•Lack of stimulation in the home environment
Support for exclusive breastfeeding
Improved maternal self efficacy can decrease postpartum depression, and improve bonding and caregiving styles
New-born Behavioural Assessment (NBO)
Train research staff to deliver NBO
Increase maternal self-efficacy
Improvement in maternal self-efficacy
Effective means of engaging mothers in responsive and nurturing care
Biobehavioural feedback is effective as a parenting intervention
Biobehavioural feedback
Provide regular biobehavioural feedback and individualised guidance
Increase developmentally appropriate play
Responsive and nurturing caregiving and improved mother-infant interactions
Improved infant growth
Among South African mothers:
•High prevalence of depression and anxiety
•Low SES and access to resources
•Non-nuclear caregiving environment
•Poor health literacy
Health literacy
Improved health literacy will improve maternal self-efficacy
Health literacy content via Mobile App
Telephonic/ in-person contact
Provide women with access to resources and activities using various delivery mechanisms
Increased health literacy
Exclusive breastfeeding
Improved infant motor and cognitive development
Better infant adherence to movement guidelines
Postpartum mental health support
Supporting parents mental health is essential for encouraging nurturing relationships
Mental health screening and support
Team psychologist for individual counselling and referrals
Support maternal mental health

V2.1_20_10_2022

Play Love And You (PLAY) Study: Consent form

1. I have been given a Participant Information Sheet which explains the nature and processes involved in this study, which is attached hereto;
2. I was given time to read it, or had it read to me, in the language I best understand;
3. I was given time to ask any questions I wanted to and found any answers given to me to be reasonable and satisfactory;
4. I believe I fully understand why the study is being conducted and what the intended outcomes will be;
5. I understand that there will be no immediate benefit to me, should I agree to participate, nor will I receive any payment; conversely, participation will not cost me anything but my time;
6. I understand that, even if I initially consent to take part in the study, I may subsequently withdraw at any time and would not be required to give any reasons; if that happened, any data collected about me for the purposes of the study would immediately be destroyed, unless I give consent for it to be retained
7. I have been given a range of contact details, listed below. If I require further information or become concerned about any aspect of this study, I am free to speak to any of these contacts.

Contact details:

- Dr Alessandra Prioreschi, Principal Investigator, telephone no. 083 989 0070, or by e-mail at Alessandra.Prioreschi@wits.ac.za,
- Professor CB Penny, Chairperson of the Human Research Ethics Committee (Medical) at the University of Witwatersrand, on telephone no. 011 717 2301, or by e-mail at Clement.Penny@wits.ac.za.
- Ms. Z Ndlovu or Mr Rhulani Mkansi, Committee Secretariat, telephone nos.: 011 717 2700 or 1234, or by e-mail at: Zanele.Ndlovu@wits.ac.za or Rhulani.Mkansi@wits.ac.za
- Mrs Sarah Cantrell, Psychologist, Telephone no 0824649381 or by e-mail at sarahcantrellphd@gmail.com

Name of Participant: ____
Date: ____
Place: ____
Signature or mark ____

Witnessed by:
Name of Witness: ____
Signature: ____
Date: ____

For peer review only

V2.1_20_10_2022

CONSENT FORM FOR VISUAL RECORDING OF STUDY PARTICIPATION

I have read and understood the project information sheet, and I understand that it is up to me whether or not my videos are viewed by the research team. I understand that if I do not wish for my videos to be seen, it will not in any way affect how the interviewer treats me. I understand that my data will be shared with researchers in the United Kingdom. I understand that I can ask the research team to delete my videos, or any part of my videos at any time. I understand that the information that I give will be treated in the strictest of confidence, and that my name will not be used when the videos are analysed.

I hereby consent to visual recording of my interactions with my baby using the Headcam devices.

I understand that:

- The recording will be stored in a secure location (a locked cupboard or password protected computer) with restricted access by the researcher and the research supervisor.
- The recording will be transcribed and any information that could identify me will be removed from the transcription.
- My face and voice, and my baby's face and voice will be visible and audible in the recordings.
- The recordings will normally be erased within either (a) two (2) years of the publication of the research findings, or (b) six (6) years, if no publications arise from this research, or five years after the study.
- The film, with all identifying information directly linked to me removed, will be stored permanently and may be used for future research.
- Anyone wishing to access this information in the future will first have to obtain the approval of the Human Research Ethics Committee (Medical) of the University of the Witwatersrand, Johannesburg.

Name of Participant: ____________________
Date: ____________________
Place: ____________________
Signature or mark ____________________

Witnessed by:
Name of Witness: ____________________
Signature: ____________________
Date: ____________________

For peer review only

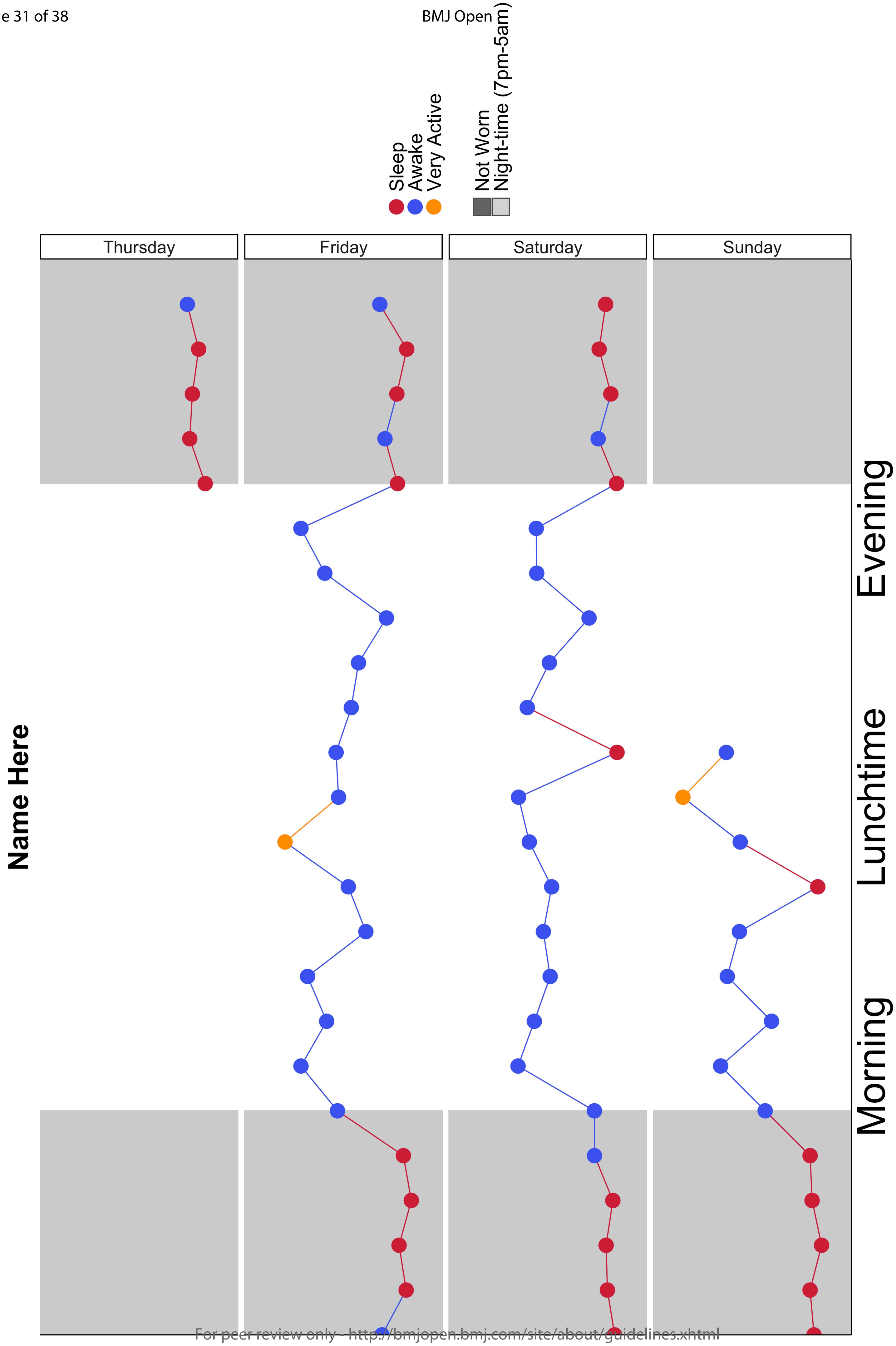

For peer review only - http://bmjopen.bmj.com/site/about/guidelines.xhtml

Supplementary Figure 2. Sleep, screen time, routines and daily activities

Introductory open-ended conversation – how is mom? How is baby?

↓

QUESTION 1: SLEEP

- How often does baby sleep and for how long (at night and naps)?
- What is the quality of baby's sleep? Does baby settle easily? Does baby wake up often?

↓

Sleeps well

Difficulty with sleeping/ not sleeping enough

↓

DISCUSSION:

What are healthy sleeping routines?

- Having same routine before bed
- Going to bed at similar time
- Reducing noise and light at bedtimes
- No background screens & recommendations for no

→

MATERIALS TO SEND:

- baby massage video
- examples of routine (bath, bottle, bed)

↓

QUESTION 2: When baby is not sleeping/ eating, what is she/ he doing?

↓

DISCUSSION:

- developmental milestones e.g. rolling, sitting
- age and resource appropriate activities to do with baby
- how to encourage free play

↓

MATERIALS TO SEND:

- Video on how to encourage motor development
- How to make musical instruments out of home materials
- Safety information on letting babies play freely without constraint

For peer review only - http://bmjopen.bmj.com/site/about/guidelines.xhtml

Professor and Head of School of Computer Science,

Electrical and

University of [illegible]

SPIRIT

STANDARD PROTOCOL ITEMS: RECOMMENDATIONS FOR INTERVENTIONAL TRIALS

SPIRIT 2013 Checklist: Recommended items to address in a clinical trial protocol and related documents*

Section/item	Item No	Description	Page number
**Administrative information**			
Title	1	Descriptive title identifying the study design, population, interventions, and, if applicable, trial acronym	Title page
Trial registration	2a	Trial identifier and registry name. If not yet registered, name of intended registry	2
	2b	All items from the World Health Organization Trial Registration Data Set	
Protocol version	3	Date and version identifier	2
Funding	4	Sources and types of financial, material, and other support	14
Roles and responsibilities	5a	Names, affiliations, and roles of protocol contributors	1, 14
	5b	Name and contact information for the trial sponsor	
	5c	Role of study sponsor and funders, if any, in study design; collection, management, analysis, and interpretation of data; writing of the report; and the decision to submit the report for publication, including whether they will have ultimate authority over any of these activities	14

For peer review only

	5d	Composition, roles, and responsibilities of the coordinating centre, steering committee, endpoint adjudication committee, data management team, and other individuals or groups overseeing the trial, if applicable (see Item 21a for data monitoring committee)	
**Introduction**			
Background and rationale	6a	Description of research question and justification for undertaking the trial, including summary of relevant studies (published and unpublished) examining benefits and harms for each intervention	4-6
	6b	Explanation for choice of comparators	6-7
Objectives	7	Specific objectives or hypotheses	6-7
Trial design	8	Description of trial design including type of trial (eg, parallel group, crossover, factorial, single group), allocation ratio, and framework (eg, superiority, equivalence, noninferiority, exploratory)	7
**Methods: Participants, interventions, and outcomes**			
Study setting	9	Description of study settings (eg, community clinic, academic hospital) and list of countries where data will be collected. Reference to where list of study sites can be obtained	7
Eligibility criteria	10	Inclusion and exclusion criteria for participants. If applicable, eligibility criteria for study centres and individuals who will perform the interventions (eg, surgeons, psychotherapists)	7
Interventions	11a	Interventions for each group with sufficient detail to allow replication, including how and when they will be administered	7-10; Figure 1 and 2
	11b	Criteria for discontinuing or modifying allocated interventions for a given trial participant (eg, drug dose change in response to harms, participant request, or improving/worsening disease)	NA
	11c	Strategies to improve adherence to intervention protocols, and any procedures for monitoring adherence (eg, drug tablet return, laboratory tests)	11-12

For peer review only

	11d	Relevant concomitant care and interventions that are permitted or prohibited during the trial	7-10
Outcomes	12	Primary, secondary, and other outcomes, including the specific measurement variable (eg, systolic blood pressure), analysis metric (eg, change from baseline, final value, time to event), method of aggregation (eg, median, proportion), and time point for each outcome. Explanation of the clinical relevance of chosen efficacy and harm outcomes is strongly recommended	10-12; Figure 2
Participant timeline	13	Time schedule of enrolment, interventions (including any run-ins and washouts), assessments, and visits for participants. A schematic diagram is highly recommended (see Figure)	Table 1; Figure 2
Sample size	14	Estimated number of participants needed to achieve study objectives and how it was determined, including clinical and statistical assumptions supporting any sample size calculations	13
Recruitment	15	Strategies for achieving adequate participant enrolment to reach target sample size	7; 11; 13
**Methods: Assignment of interventions (for controlled trials)**			
Allocation:			
Sequence generation	16a	Method of generating the allocation sequence (eg, computer-generated random numbers), and list of any factors for stratification. To reduce predictability of a random sequence, details of any planned restriction (eg, blocking) should be provided in a separate document that is unavailable to those who enrol participants or assign interventions	7
Allocation concealment mechanism	16b	Mechanism of implementing the allocation sequence (eg, central telephone; sequentially numbered, opaque, sealed envelopes), describing any steps to conceal the sequence until interventions are assigned	7
Implementation	16c	Who will generate the allocation sequence, who will enrol participants, and who will assign participants to interventions	7

For peer review only - http://bmjopen.bmj.com/site/about/guidelines.xhtml

Blinding (masking)	17a	Who will be blinded after assignment to interventions (eg, trial participants, care providers, outcome assessors, data analysts), and how	7
	17b	If blinded, circumstances under which unblinding is permissible, and procedure for revealing a participant's allocated intervention during the trial	NA
**Methods: Data collection, management, and analysis**			
Data collection methods	18a	Plans for assessment and collection of outcome, baseline, and other trial data, including any related processes to promote data quality (eg, duplicate measurements, training of assessors) and a description of study instruments (eg, questionnaires, laboratory tests) along with their reliability and validity, if known. Reference to where data collection forms can be found, if not in the protocol	10-13
	18b	Plans to promote participant retention and complete follow-up, including list of any outcome data to be collected for participants who discontinue or deviate from intervention protocols	7; 11; 13
Data management	19	Plans for data entry, coding, security, and storage, including any related processes to promote data quality (eg, double data entry; range checks for data values). Reference to where details of data management procedures can be found, if not in the protocol	13-14
Statistical methods	20a	Statistical methods for analysing primary and secondary outcomes. Reference to where other details of the statistical analysis plan can be found, if not in the protocol	13; Figure 2
	20b	Methods for any additional analyses (eg, subgroup and adjusted analyses)	Figure 2
	20c	Definition of analysis population relating to protocol non-adherence (eg, as randomised analysis), and any statistical methods to handle missing data (eg, multiple imputation)	13
**Methods: Monitoring**			

Data monitoring	21a	Composition of data monitoring committee (DMC); summary of its role and reporting structure; statement of whether it is independent from the sponsor and competing interests; and reference to where further details about its charter can be found, if not in the protocol. Alternatively, an explanation of why a DMC is not needed	
	21b	Description of any interim analyses and stopping guidelines, including who will have access to these interim results and make the final decision to terminate the trial	Figure 2
Harms	22	Plans for collecting, assessing, reporting, and managing solicited and spontaneously reported adverse events and other unintended effects of trial interventions or trial conduct	11-12
Auditing	23	Frequency and procedures for auditing trial conduct, if any, and whether the process will be independent from investigators and the sponsor	
**Ethics and dissemination**			
Research ethics approval	24	Plans for seeking research ethics committee/institutional review board (REC/IRB) approval	20-21
Protocol amendments	25	Plans for communicating important protocol modifications (eg, changes to eligibility criteria, outcomes, analyses) to relevant parties (eg, investigators, REC/IRBs, trial participants, trial registries, journals, regulators)	2; 15
Consent or assent	26a	Who will obtain informed consent or assent from potential trial participants or authorised surrogates, and how (see Item 32)	14-15
	26b	Additional consent provisions for collection and use of participant data and biological specimens in ancillary studies, if applicable	
Confidentiality	27	How personal information about potential and enrolled participants will be collected, shared, and maintained in order to protect confidentiality before, during, and after the trial	7; 13-14

For peer review only - http://bmjopen.bmj.com/site/about/guidelines.xhtml

Declaration of interests	28	Financial and other competing interests for principal investigators for the overall trial and each study site	14-15
Access to data	29	Statement of who will have access to the final trial dataset, and disclosure of contractual agreements that limit such access for investigators	14
Ancillary and post-trial care	30	Provisions, if any, for ancillary and post-trial care, and for compensation to those who suffer harm from trial participation	
Dissemination policy	31a	Plans for investigators and sponsor to communicate trial results to participants, healthcare professionals, the public, and other relevant groups (eg, via publication, reporting in results databases, or other data sharing arrangements), including any publication restrictions	
	31b	Authorship eligibility guidelines and any intended use of professional writers	14
	31c	Plans, if any, for granting public access to the full protocol, participant-level dataset, and statistical code	
**Appendices**			
Informed consent materials	32	Model consent form and other related documentation given to participants and authorised surrogates	Supplementary
Biological specimens	33	Plans for collection, laboratory evaluation, and storage of biological specimens for genetic or molecular analysis in the current trial and for future use in ancillary studies, if applicable	NA

*It is strongly recommended that this checklist be read in conjunction with the SPIRIT 2013 Explanation & Elaboration for important clarification on the items. Amendments to the protocol should be tracked and dated. The SPIRIT checklist is copyrighted by the SPIRIT Group under the Creative Commons "Attribution-NonCommercial-NoDerivs 3.0 Unported" license.

For peer review only
